# Phosphorylation at tyrosine 317 and 508 are crucial for PIK3CA/p110α to promote CRC tumorigenesis

**DOI:** 10.1186/s13578-023-01102-7

**Published:** 2023-09-09

**Authors:** Ting Wang, Longci Sun, Chengkun Chen, Yingchao Zhang, Baoyu He, Yanhua Zhang, Zhenghe Wang, Hanbing Xue, Yujun Hao

**Affiliations:** 1 State Key Laboratory of Oncogenes and Related Genes, Shanghai Cancer Institute, Renji Hospital, Shanghai Jiao Tong University School of Medicine, Shanghai, 200032 China; 2 Department of Gastrointestinal Surgery, Renji Hospital, School of Medicine, Shanghai Jiao Tong University, Shanghai, 200127 China; 3 Department of Laboratory Medicine, Affiliated Hospital of Jining Medical University, Jining Medical University, Jining, 272029 Shandong China; 4 Department of Genetics and Genome Sciences, Case Western Reserve University, 10900 Euclid Avenue, Cleveland, OH 44106 USA; 5 Case Comprehensive Cancer Center, School of Medicine, Case Western Reserve University, 10900 Euclid Avenue, Cleveland, OH 44106 USA; 6 Division of Gastroenterology and Hepatology, Key Laboratory of Gastroenterology and Hepatology, Renji Hospital, School of Medicine, Ministry of Health, Shanghai Jiao Tong University, Shanghai, 200127 China

**Keywords:** PI3K, p110α, Tyrosine phosphorylation, Src, Colorectal cancer

## Abstract

**Background:**

PI3K/AKT signaling pathway plays important role in tumorigenesis of human cancer. Protein phosphorylation is crucial for signaling transduction of this pathway. PIK3CA, encoding the catalytic subunit p110α of PI3K complex, is one of the most frequently mutated oncogenes in human cancers. However, phosphorylation sites of PIK3CA/p110α and their underlying mechanism in tumorigenesis are largely unknown.

**Methods:**

Tyrosine phosphorylation sites of PIK3CA/p110α are identified with Mass-Spectrum. Crispr/CAS9 strategy is applied to generate Y317F and Y508F mutant knock-in cell clones. The growth and metastasis abilities of cells are evaluated in vitro and in vivo. Phospho-proteomics analysis and Western blots are used to demonstrate downstream signaling pathways of PIK3CA/p110α tyrosine phosphorylation. In vitro kinase assay is applied to identify the kinase of PIK3CA/p110α tyrosine phosphorylation.

**Results:**

Tyrosine phosphorylation of PIK3CA/p110α is stimulated by growth factors such as EGF, HGF and PDGF. Two tyrosine residues, Y317 and Y508, are identified on PIK3CA/p110α. Either Y317 or Y508 phosphorylation is essential for tumorigenesis of CRC. Mutation at Y317 of p110α reduces the proliferation, migration, and invasion of cancer cells through Src-MLC2 pathway, while mutation at Y508 of p110α impairs AKT signaling. Moreover, Src interacts with and phosphorylates p110α.

**Conclusions:**

PIK3CA/p110α phosphorylation at Y317 and Y508 play important role in tumorigenesis of colorectal cancer through two independent pathways.

**Supplementary Information:**

The online version contains supplementary material available at 10.1186/s13578-023-01102-7.

## Introduction

PI3K/AKT signaling pathway plays important role in tumorigenesis of human cancers [1]. Normally, upon the stimulations by growth factors (GFs), the receptor tyrosine kinases (RTKs) are activated, and then recruit PI3K protein complex on the membrane to generate phosphatidylinositol-3,4,5-triphosphate (PIP3) from phosphatidylinositol-4,5-biophosphate (PIP2) [2]. The second messenger PIP3 recruits and activates 3-phosphoinositide dependent protein kinase-1 (PDK1) and AKT serine/threonine kinase on the membrane [2]. AKT then phosphorylates various substrate proteins such as mTOR, GSK3β, Foxo, NF-κB to regulate proliferation, survival, and motility of cancer cells [1–3].

Post-translational modifications (PTMs) are crucial for regulating PI3K/AKT signaling pathway, notably phosphorylation. Tyrosine phosphorylation of RTKs, or adaptor proteins such as IRS-1 facilitate their binding with p85 regulatory subunits of PI3K, and then bring PI3K complex on membrane [2]. Activation of AKT proteins is subjected to successive phosphorylation through Thr308 and Ser473 residues [4, 5]. AKTs are serine/threonine kinases that phosphorylate numbers of substrate proteins, for example GSK3β at S9 [6], Foxo1 at T24, S256, S319 and other Foxo proteins at corresponding serine/threonine residues [7–9], BAD at S136 [10], etc. In addition, PI3K/AKT/TSC/Rheb signaling-mediated mTOR phosphorylation at S1261 enhances mTORC1 kinase activity to promote cell growth [11]. PIP3 phosphatase PTEN is inactivated upon phosphorylation in its C-terminal region by several serine/threonine kinases [12]. Therefore, investigating PTMs of PI3K signaling components would give us a better understanding how to regulate this pathway and discover more therapeutic targets for cancer.

PTMs of several PI3K complex isoforms have been demonstrated. PI3K complex consists of catalytic subunit p110s and regulatory subunit p85s. p110s perform lipid kinase activity, and p85s stabilize and regulate p110s. Phosphorylation at tyrosine 688 relieves p85 inhibitory activity on p110s [13]. Phosphorylation of p85β on Tyr 655 inhibits p85β binding to F-box protein FBXL2 to prevent p110-free p85β degradation [14]. cSH2 domain S690 phosphorylation decreases p85 binding affinity to tyrosine-phosphorylated proteins resulting in less PI3K membrane localization [15]. Other than that, phosphorylation on S83, T86, Y368, Y580, and Y607 of p85α have also been identified to regulate insulin-PI3K signaling [16–18]. There are fewer reports of post-translational modifications on PI3K catalytic subunit p110s. Class III PI3K VPS34 (PIK3C3) is a critical regulator of autophagy [19]. Phosphorylation of T159, T163 and S165 on VPS34 control the class III PtdIns3 kinase activity in cell-cycle progression, development, autophagy, and human disease such as cancers [20, 21]. Moreover, SUMOylation of class IA p110β on lysine 952 stabilize p110β and activate AKT signaling [22]. However, whether PIK3CA/p110α contains PTMs and whether these PTMs play important role in biological processes have not been elucidated.

In this study, we identified two tyrosine phosphorylation sites Y317 and Y508 in PIK3CA /p110α. Y317 and Y508 are essential for PI3Kα-mediated oncogenic function. Src is a tyrosine kinase of PIK3CA/p110α.

## Results

### p110α is phosphorylated at Y317 and Y508 in human colorectal cancer cells

**Fig. 1:**
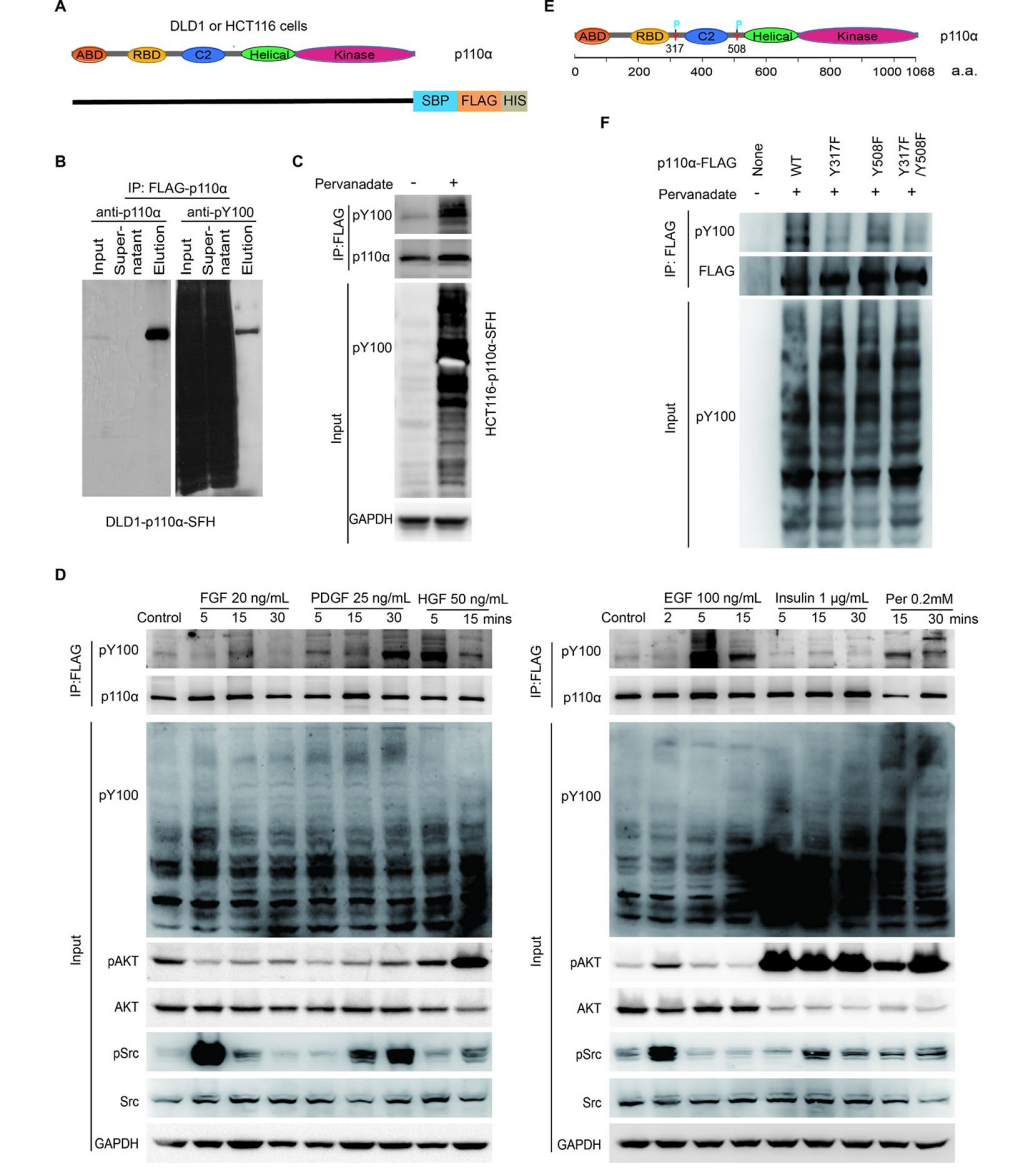
p110α protein undergoes tyrosine phosphorylation. **A** Schematic of isogenic cell lines in which endogenous p110α was tagged with SBP-3×FLAG-6×HIS. ABD: adaptor-binding domain; RBD: Ras-binding domain; C2: C2 domain; helical: helical domain; kinase: kinase domain; SBP: SBP tag; FLAG: 3×FLAG tag; HIS: 6×HIS tag. **B-C** DLD1-p110α-SFH cells (**B**) or HCT116-p110α-SFH cells (**C**) were serum-starved overnight and then treated with or without pervanadate for 30 min. Cell lysates were immunoprecipitated with FLAG agarose beads and immunocomplex were blotted with indicated antibodies. DLD1-p110α-SFH: DLD1 isogenic cells in which endogenous p110α was tagged with SBP-3×FLAG-6×HIS; HCT116-p110α-SFH: HCT116 isogenic cells in which endogenous p110α was tagged with SBP-3×FLAG-6×HIS. **D** HCT116-p110α-SFH cells were serum-starved overnight and then treated with growth factors, insulin, or pervanadate for indicated time. Cell lysates were immunoprecipitated with anti-FLAG agarose beads and immunocomplex were blotted with the indicated antibodies. FGF: fibroblast growth factor; PDGF: platelet derived growth factor BB; HGF: hepatocyte growth factor; EGF: epidermal growth factor; Per: pervanadate. **E** Schematic of p110α tyrosine phosphorylation sites. Tyrosine 317 and 508 located in the linker regions RBD-C2 and C2- Helical of p110α respectively. **F** Mutation at Y317 and/or Y508 reduced tyrosine phosphorylation of p110α. Wild-type or mutant p110α constructs were transfected into 293T cells. Cells were serum-starved overnight and then treated with or without pervanadate for 30 min. Cell lysates were immunoprecipitated with FLAG agarose beads and immunocomplex were blotted with indicated antibodies

To identify PTMs on PIK3CA/p110α, we firstly endogenously knocked in SBP-3×FLAG-6×HIS tag at the C-terminal of p110α in DLD1 cells (named as DLD1-p110α-SFH cells) and HCT116 cells (HCT116-p110α-SFH cells) by recombinant adeno-associated virus (rAAV)-mediated homologous recombination (Fig. 1A, S1A and S1B). DLD1-p110α-SFH cells were treated with pervanadate (tyrosine phosphatase inhibitor) and PhosSTOP (phosphatase inhibitors) for thirty minutes, then p110α were pulled down with anti-FLAG agarose beads. Interestingly, p110α exhibited obvious tyrosine phosphorylation when the immunoprecipitates were blotted with a phospho-tyrosine monoclonal antibody (pY-100) (Fig. 1B). Similar results were obtained from HCT116- p110α-SFH cells (Fig. 1C). We then determined whether p110α underwent tyrosine phosphorylation under growth factors stimulation. HCT116-p110α-SFH cells were treated with fibroblast growth factor (FGF), platelet derived growth factor (PDGF), hepatocyte growth factor (HGF), epidermal growth factor (EGF), or insulin for various times. Cells were lysed under native conditions and p110α were immunoprecipitated with anti-FLAG agarose beads. As shown in Fig. 1D, PDGF, HGF, and EGF significantly increased tyrosine phosphorylation levels of p110α, although at different time points. Under EGF and HGF stimulation, tyrosine phosphorylation of p110α were observed with highest level after 5 min treatment, and dramatically decreased at 15 min (Fig. 1D). Under PDGF stimulation, tyrosine phosphorylation on p110α were not detected until 30 min (Fig. 1D). In addition, FGF moderately stimulated p110α phosphorylation (Fig. 1D). However, insulin treatment had no impact on p110α tyrosine phosphorylation (Fig. 1D). As a positive control, pervanadate consistently increased tyrosine phosphorylation of p110α (Fig. 1D). p110α was purified under denaturing conditions with HIS-tag in HCT116-p110α-SFH cells. Tyrosine phosphorylation sites on p110α were then examined by Mass Spectrometry analysis. Two tyrosine (Y) sites, Y317 and Y508, on p110α were identified to be phosphorylated (Table S1). Analysis of the amino acid sequence revealed that Y317 and Y508 were located at RBD-C2 linker region and C2-Helical linker region of p110α respectively (Fig. 1E). Y317F and/or Y508F mutations significantly decreased p110α tyrosine phosphorylation (Fig. 1F). These data suggest that p110α occurs tyrosine phosphorylation at Y317 and Y508.

### Y317 and Y508 are critical for p110α to promote CRC tumorigenesis

PIK3CA/p110α is one of the most frequently mutated oncogenes and a key therapeutic target in colorectal cancer. To assess whether phosphorylation of p110α affects the CRC progression, we endogenously mutated Y317 or Y508 to F (phenylalanine) on PIK3CA locus using CRISPR/Cas9 mediated genome editing strategy in colon cancer cells (HCT116, DLD1, SW480, LoVo) (Fig. S1C and S1D). Homozygous mutation of Y317F knock-in (KI) cell clones and heterozygous mutation of Y508F KI cell clones were successfully generated (Fig. S1C and S1D). The mRNA and protein levels of PIK3CA/p110α in Y317F or Y508F KI mutant clones were evaluated. Compared with their parental cells, Y317F or Y508F KI mutant clones had similar PIK3CA/p110α expression levels, suggesting that abolishing p110α phosphorylation had no impact on their transcription or translation processes (Fig. S1E and S1F).

**Fig. 2:**
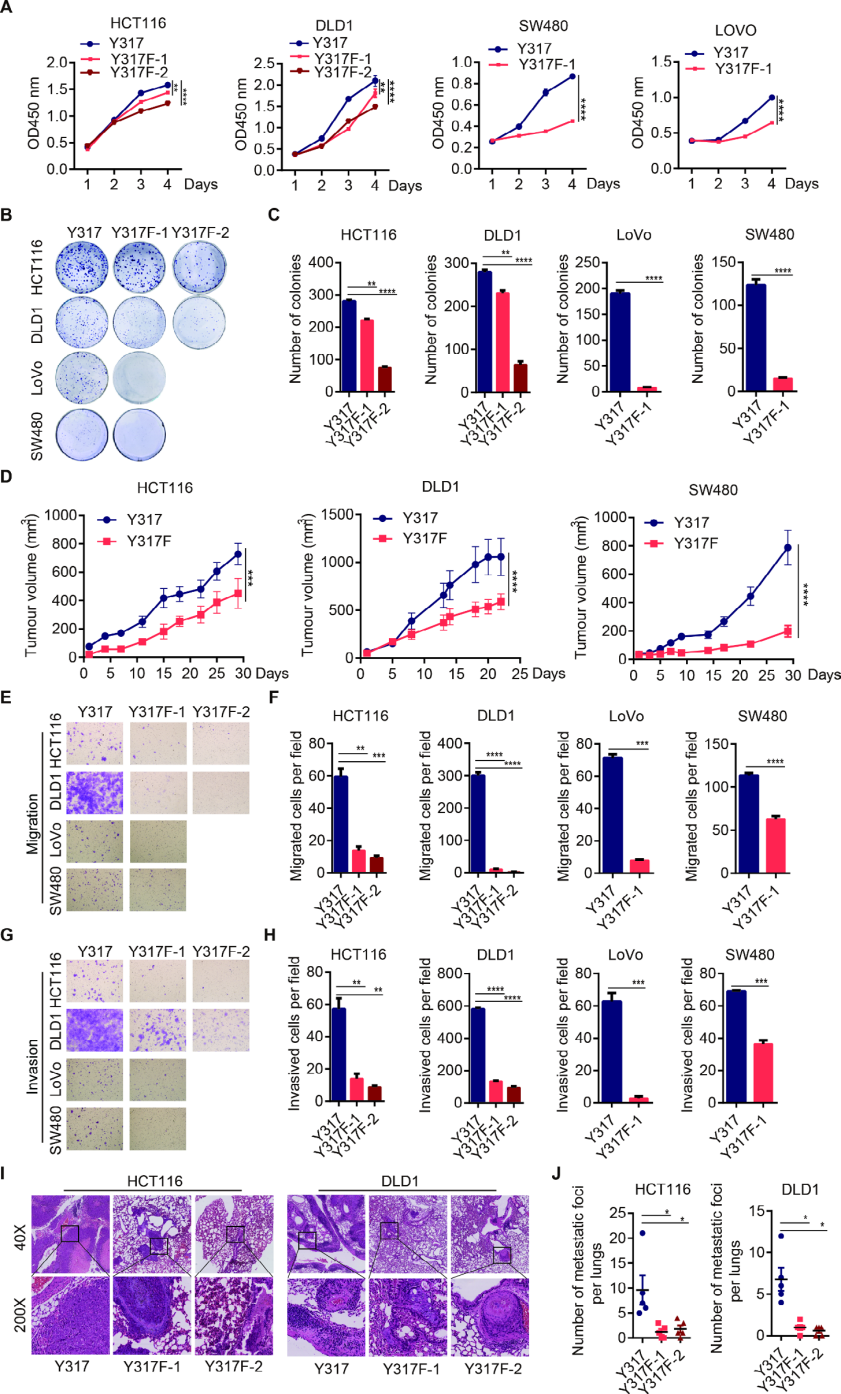
p110α Y317F mutation impairs the proliferation, migration, and invasion of CRC cells. **A-D** p110α Y317F mutation impaired the growth of CRC cells. Cell lines with indicated genotype were analyzed for cell proliferation (**A**), colony formation (**B**, **C**), and xenograft tumor growth (**D**). Y317 indicates parental cells; Y317F indicates p110α Y317F KI mutant cells. **E-J** p110α Y317F mutation impaired CRC metastasis. Cell lines with indicated genotype were analyzed for cell migration (**E, F**) and invasion (**G**, **H**) by transwell assays. The indicated cells were injected into nude mice through tail veins. Two months later, mice were sacrificed. The H&E staining images (**I**) and numbers of lung metastatic nodules (**J**) were present. Two-tailed unpaired *t* test (C, F, H, J) and two-way ANOVA (A, D), *p < 0.05, **p < 0.01, ***p < 0.001, ****p < 0.0001

We then examined the function of p110α Y317 phosphorylation in CRC tumorigenesis. Compared with their parental cells, Y317F mutation moderately reduced the proliferation and colony formation abilities of CRC cells including HCT116, DLD1, LoVo, and SW480 (Fig. 2A-2C). Y317F mutation in HCT116, DLD1 and SW480 cells also significantly reduced the growth of subcutaneous xenograft tumors in vivo (Fig. 2D, S2A and S2B). More importantly, Y317F mutation dramatically decreased the migration and invasion abilities of these four cell lines in vitro (Fig. 2E-2H, S2C and S2D). With tail-vein injection experiments, we evaluated the metastatic ability of parental cells and their corresponding Y317F KI mutant cells in vivo. The data showed that mutation of Y317 on p110α significantly reduced the sizes and numbers of metastatic nodules in the lungs of nude mice (Fig. 2I, 2J and S2E).

**Fig. 3:**
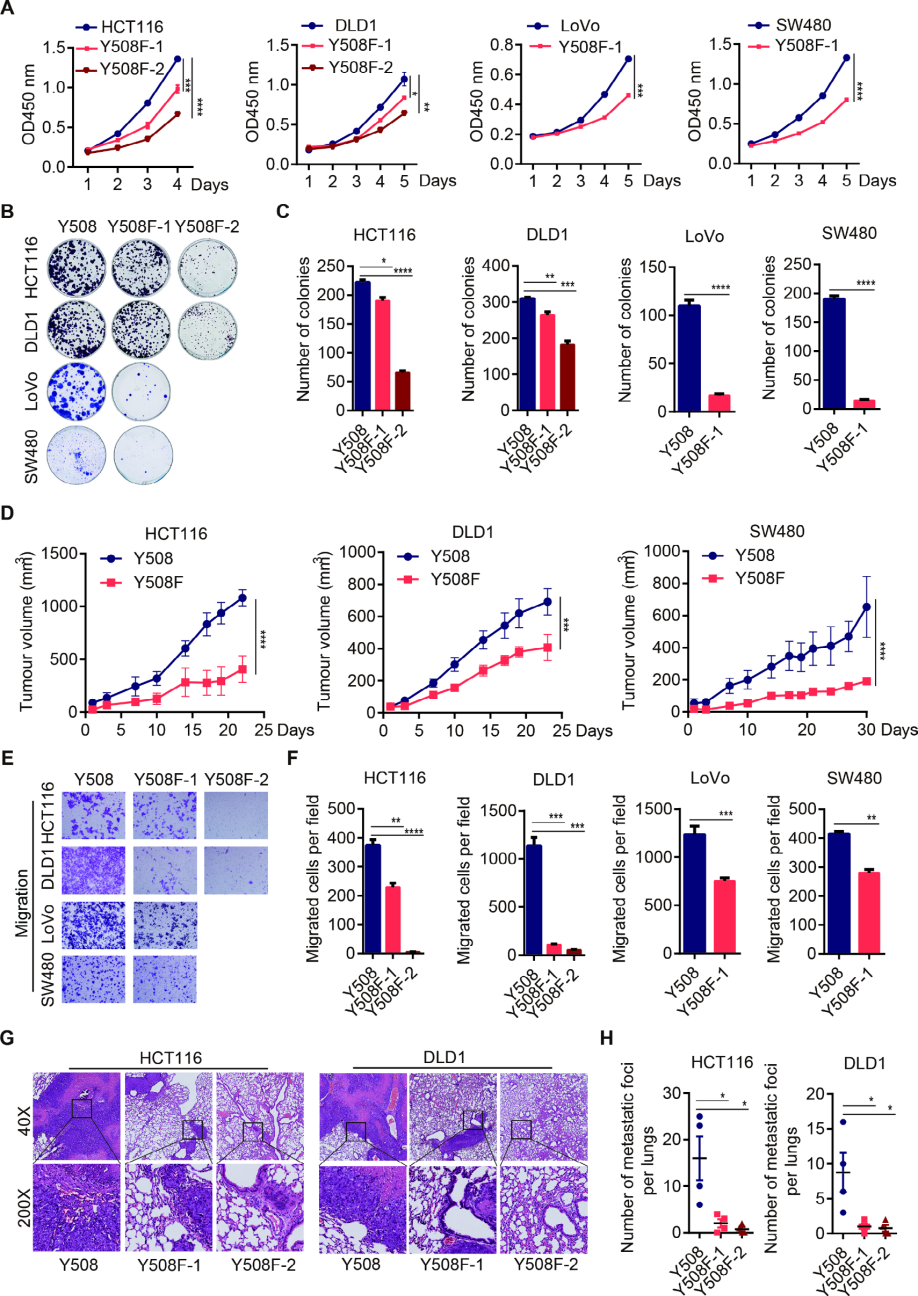
p110α Y508F heterozygous mutation impairs the proliferation, migration, and invasion of CRC cells. **A-D** p110α Y508F heterozygous mutation impaired the growth of CRC cells. Parental cells and isogenic p110α Y508F KI mutant cells were analyzed for proliferation (**A**), colony formation (**B, C**), and xenograft tumor growth (**D**). **E-H** p110α Y508F heterozygous mutation attenuated metastasis of CRC cells. The migration ability of parental cells and isogenic p110α Y508F KI mutant cells were analyzed in vitro by transwell assays (**E, F**). The indicated cells were injected into nude mice through tail veins. Two months later, mice were sacrificed. The H&E staining images (**G**) and numbers of lung metastatic nodules (**H**) were present. Two-tailed unpaired *t* test (C, F, H) and two-way ANOVA (A, D), *p < 0.05, **p < 0.01, ***p < 0.001, ****p < 0.0001

Next, we assessed the function of p110α Y508 on tumorigenesis. Although the isogenic cell colonies with homozygous Y508F mutations on PIK3CA locus could not be able to generate, heterozygous mutation of p110α Y508 already inhibited the proliferation, colony formation abilities of CRC cells in vitro and the growth of xenograft tumors in vivo (Fig. 3A-3D and S3A-S3E). Moreover, Y508F heterozygous mutation on p110α impaired the abilities of cell migration in vitro (Fig. 3E, 3F and S3F), and reduced the sizes and numbers of lung metastasis nodules in vivo (Fig. 3G, 3H, and S3G).

Taken together, these results suggest that two tyrosine phosphorylation sites Y317 and Y508 are essential for p110α to promote CRC tumorigenesis.

### Tyrosine phosphorylation of p110α Y317 mediates the activation of Src-MLC2 signaling

To determine how tyrosine phosphorylation of p110α Y317 affects tumorigenesis, we first detected the classic PI3K/AKT signaling including phosphorylation levels of AKT and its downstream substrates in parental cells and p110α Y317F KI mutant cells. It was observed that p110α Y317F mutation had no effect on phosphorylation of AKT and its downstream substrates, such as GSK3β, mTOR, p70S6K (Fig. S4A). p110α Y317F mutation did not perturb AKT activation upon insulin or EGF stimulation (Fig. S4B). As p110α Y317F mutation dramatically affect the motility of colorectal cancer cells, the protein levels of E-cadherin and β-catenin (markers of Epithelial-Mesenchymal Transition, EMT), were also evaluated in HCT116 and HCT116 p110α Y317F KI mutant cells. The results indicated that E-cadherin and β-catenin expression levels were similar in HCT116 and HCT116 p110α Y317F KI mutant cells, suggesting that the p110α Y317F mutation didn’t attenuate metastasis through EMT pathway (Fig. S4C).

**Fig. 4:**
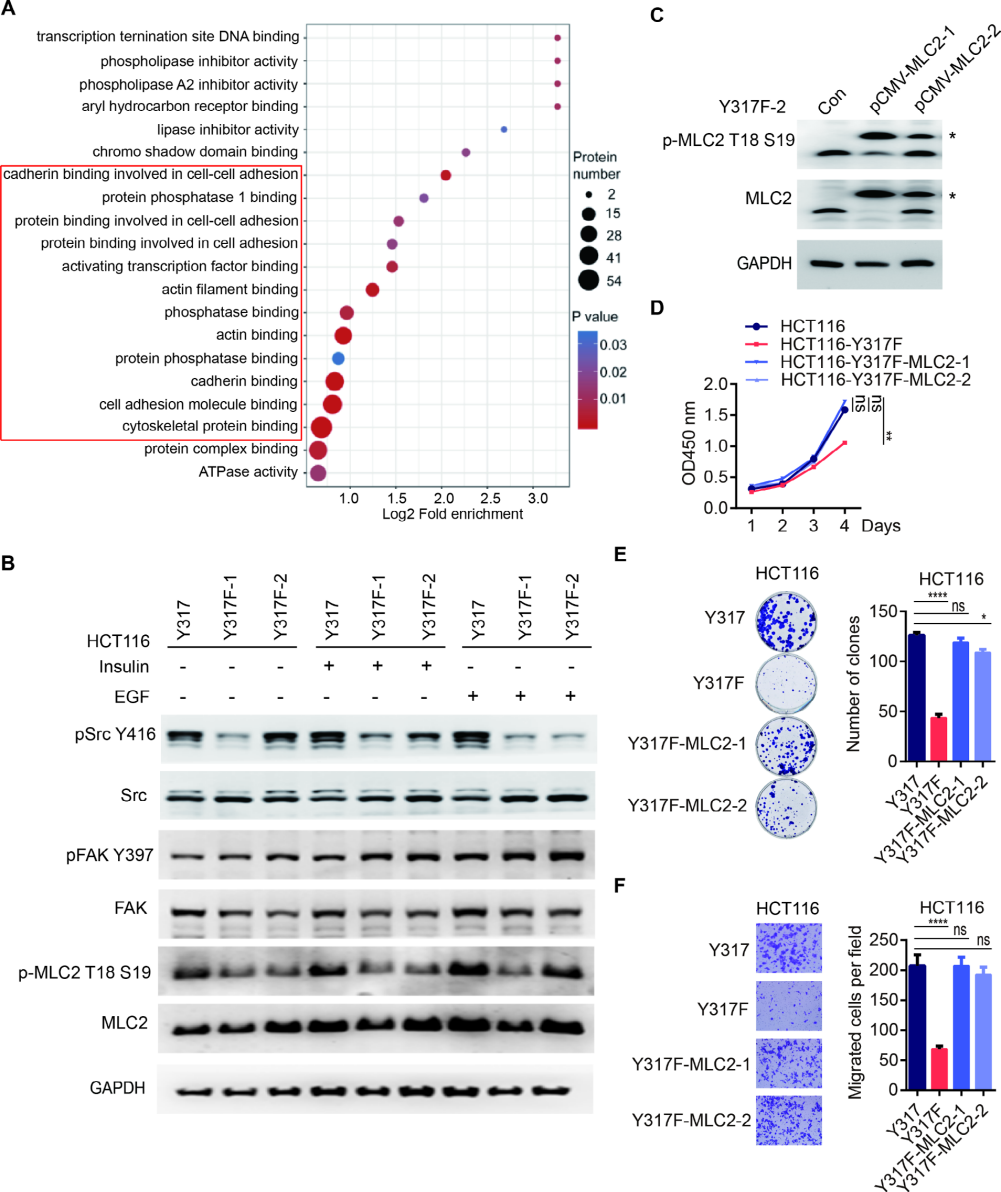
p110α Y317F mutation attenuates Src-MLC2 signaling pathway. **A** The bubble diagram of GO analysis with differentially phosphorylated proteins between HCT116 parental cells and HCT116 Y317F KI mutant cells. **B** HCT116 parental cells and HCT116 Y317F KI mutant cells were serum-starved overnight and then treated with EGF or insulin for 15 min. Cell lysates were collected and blotted with indicated antibodies. **C-F** MLC2 reconstitution rescues the proliferation and migration defects caused by p110α Y317F mutation. MLC2 was overexpressed in HCT116 p110α Y317F KI mutant cells. p-MLC2 and total MLC2 protein levels were evaluated by Western blots (**C**). The asterisk indicates exogenously overexpressed MLC2. Cell lines including HCT116, HCT116 Y317F KI mutant, HCT116 Y317F KI mutant reconstituted with MLC2 were analyzed with for proliferation (**D**); colony formation (**E**); and migration (**F**). Two-tailed unpaired *t* test (E, F) and two-way ANOVA (D), *p < 0.05, **p < 0.01, ****p < 0.0001, ns, not significant

To further identify the downstream signaling mediated by tyrosine phosphorylation of p110α Y317, we performed phospho-proteomics analysis (Table S2). The differentially phosphorylated proteins (DPPs) in HCT116 and HCT116 Y317F KI mutant cells were plotted in the bubble diagram of Gene Ontology (GO) enrichment analysis (Fig. 4A). Gene ontology (GO) and Kyoto Encyclopedia of Genes and Genomes (KEGG) pathway enrichment analyses revealed that many DPPs were involved in cell-cell adhesion, actin binding, and cytoskeletal protein binding, etc. (Fig. 4A and S4D). Among those pathways, Src-(FAK)-RhoA-(MLCP)-MLC-Actin signaling in focal adhesion caught our attention (Fig. S4D). The phosphorylation levels of Src, FAK, and MLC2 in this pathway were examined (Fig. 4B and S4E). It was found that phosphorylation levels of Src Y416 and MLC2 T18/S19 were obviously reduced in p110α Y317F KI mutant cells compared to those in parental cells, especially upon EGF stimulation.

To confirm whether oncogenic function of p110α Y317 phosphorylation in CRC was mediated by MLC2, MLC2 was overexpressed in HCT116 p110α Y317F KI mutant cells (Fig. 4C). The results showed that overexpression of MLC2 significantly rescued the proliferation and migration of p110α Y317F KI mutant cells (Fig. 4D-4F). Taken together, these data suggest that p110α Y317 phosphorylation affects CRC progression through Src-MLC2 signaling pathway.

### p110α Y508 phosphorylation activates AKT signaling

**Fig. 5:**
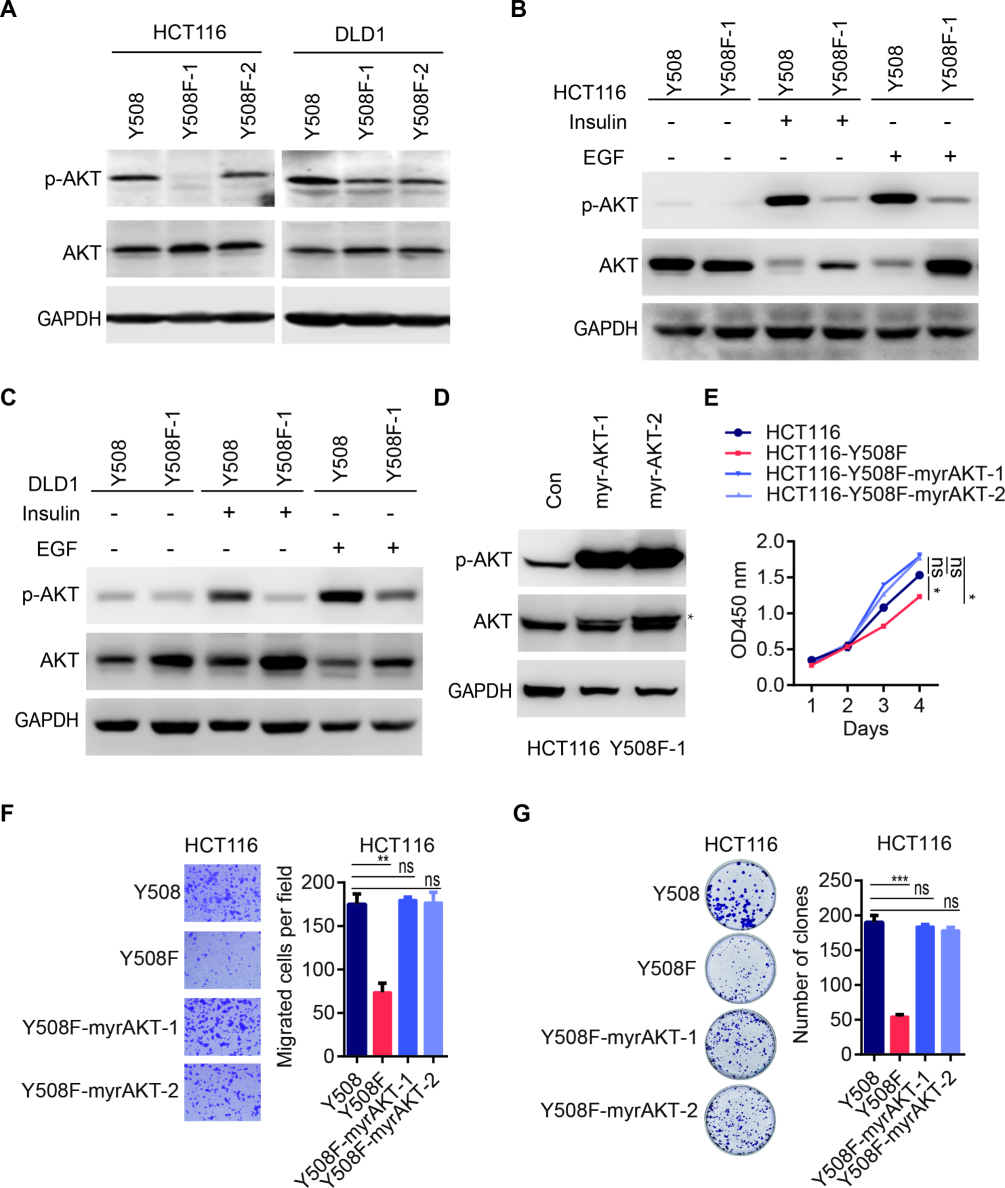
p110α Y508F heterozygous mutation impairs AKT phosphorylation. **A-C** p110α Y508F mutation affects AKT phosphorylation. Parental cells and their corresponding p110α Y508F KI mutant cells were serum-starved overnight (**A**) and then treated with EGF or insulin for 15 min (**B, C**). Cell lysates were blotted with indicated antibodies. **D-G** AKT reconstitution rescues the proliferation and migration defects caused by p110α Y508F heterozygous mutation. Myristoylated-AKT1 (myr-AKT) was overexpressed in HCT116 p110α Y508F KI mutant cells. p-AKT and total AKT protein levels were evaluated by Western blots (**D**). Indicated cells were analyzed with for proliferation (**E**); migration (**F**); and colony formation (**G**). Two-tailed unpaired *t* test (F, G) and two-way ANOVA (E), *p < 0.05, **p < 0.01, ***p < 0.001, ns, not significant

To determine the mechanism of how p110α Y508 phosphorylation influences CRC progression, we also examined the AKT phosphorylation levels in parental cells and p110α Y508F KI mutant cells. Unlike tyrosine 317, mutation of Y508 on p110α decreased phosphorylation levels of AKT (Fig. 5A). Furthermore, comparing with HCT116 and DLD1 parental cells, p110α Y508F KI mutant cells had much less phosphorylated AKT levels upon insulin or EGF stimulation (Fig. 5B and 5C). Of note, p110α Y508F mutation did not influence Src phosphorylation regardless of whether under serum starvation conditions or when stimulated by insulin or EGF (Fig. S5A). Moreover, analysis of the protein levels of E-cadherin and β-catenin revealed that Y508 phosphorylation of p110α did not affect the EMT pathway (Fig. S5B). To confirm whether oncogenic function of p110α Y508 phosphorylation was mediated by AKT, we overexpressed a constitutively activated AKT1 (Myristoylated-AKT1, myr-AKT) in HCT116 p110α Y508F KI mutant cells (Fig. 5D). As shown in Fig. 5D-5G, overexpression of myr-AKT could rescue the proliferation and migration of p110α Y508F KI mutant cells. These data suggest that p110α Y508 phosphorylation affects CRC progression through AKT signaling.

### SRC interacts with and phosphorylates p110α

**Fig. 6:**
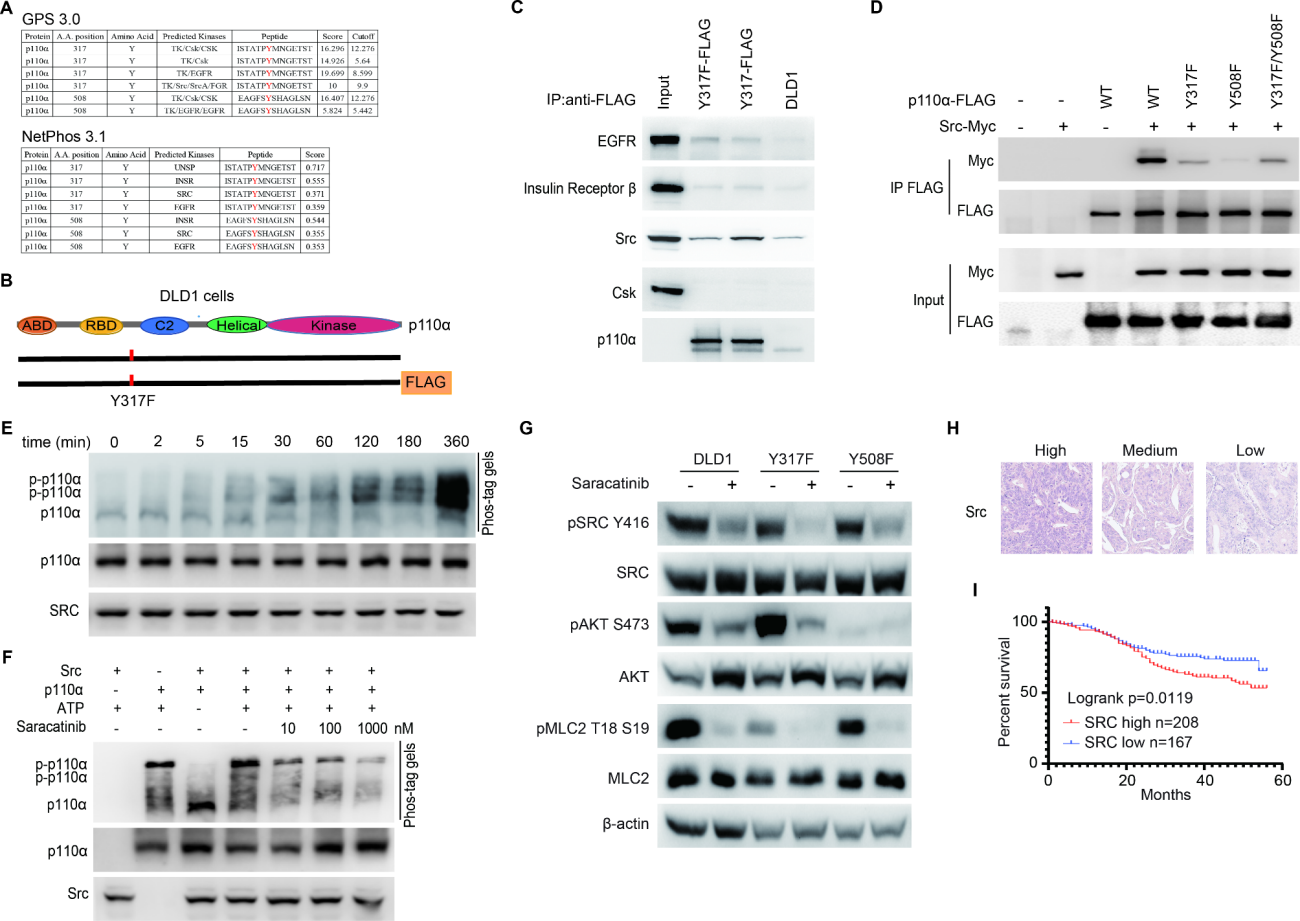
Src is a tyrosine kinase of p110α. **A** The tyrosine kinases of p110α Y317 and Y508 were predicted by GPS 3.0 and NetPhos 3.1. **B** Schematic of isogenic DLD1 p110α Y317F FLAG tagged cell line. DLD1 p110α 3×FLAG tagged cell line [23] were endogenously knocked in Y317F mutation on PIK3CA locus. **C** p110α interacts with Src. DLD1 cells, DLD1 p110α FLAG tagged cells, or DLD1 p110α Y317F FLAG tagged cells were lysed and immunoprecipitated with anti-FLAG agarose beads. The immunocomplex were blotted with indicated antibodies. **D** Y317 or Y508 mutation block the interaction between p110α and Src. HEK293T cells were transfected with indicated plasmids. Cells lysates were immunoprecipitated with anti-FLAG agarose beads, followed by Western blot analysis with indicated antibodies. The FLAG-tagged p110α constructs included wild-type p110α (WT); p110α Y317F mutant protein (Y317F); p110α Y508F mutant protein (Y508F); and p110α Y317F Y508F double mutant protein (Y317F/Y508F). **E** In vitro tyrosine kinase assay by Phospho-tag SDS-PAGE strategy. p110α/p85α protein complex were purified with Bac-to-Bac baculovirus expression system. Src proteins were purified with bacterial protein expression system. p110α and Src proteins were incubated at 37℃ in tyrosine kinase buffer for indicated time and reactions were stopped by boiling samples. Samples were dissolved with phospho-tag SDS-PAGE to detect p110α phosphorylation, or with SDS-PAGE gel to quantify the loading amounts of proteins. **F** Src inhibitor suppresses phosphorylation of p110α. p110α/p85α protein complex and Src recombinant proteins were incubated with or without Src inhibitor Saracatinib. p110α phosphorylation were detected with Phospho-tag SDS-PAGE strategy. **G** Src inhibition suppresses phosphorylation of MLC2 and AKT. Indicated cell lines were treated with Saracatinib for 36 h and then cell lysates were blotted with indicated antibodies. **H-I** Src IHC staining were performed with CRC tissue microarray (375 CRC patients samples from Renji Hospital). Src staining were showed in (**H**) and overall survival were calculated with Kaplan-Meier survival analysis (**I**)

GPS 3.0 and NetPhos 3.1 were applied to predict the tyrosine kinases which might phosphorylate p110α at Y317 or Y508 (Fig. 6A). The results indicated that CSK, EGFR, insulin receptor (INSR), and Src were potential candidates. Then we determined the interaction between p110α and these kinase candidates. A p110α FLAG tagged DLD1 cell clone [23] was endogenously knocked in p110α Y317F mutations on PIK3CA locus (Fig. 6B). FLAG-tagged p110α or FLAG-tagged p110α Y317F mutant proteins was pulled down in these two isogenic cell lines with anti-FLAG agarose beads, and then blotted with candidate kinases. The results showed that Src, but not CSK, EGFR, or INSR, interacted with p110α (Fig. 6C). Other Src family kinases were also tested for their interaction ability with p110α, and the results showed Src, but not Fyn or Lyn, associated with p110α (Fig. S6A-S6C). Since Src could interact p85, we deleted p85 binding domain (ABD) in p110α protein to verify whether Src-p110α interaction is through p85 (Fig. S6D). As shown in Fig. S6E, p110α delABD protein which didn’t interact with p85 still associated with Src, suggesting that the interaction between p110α and Src is direct. The p110α-Src interaction was interrupted by p110α Y317F mutation (Fig. 6C and 6D). Moreover, Y508F mutation or Y317F/Y508F double mutations on p110α disrupt the interaction between Src and p110α (Fig. 6D). Furthermore, to evaluate the potential role of Src as tyrosine kinase of p110α, p110α and Src recombinant proteins were purified for in vitro kinase assay (Fig. 6E and 6F). Phospho-tag SDS-PAGE is a phosphate-affinity electrophoresis technique that separates phosphorylated and non-phosphorylated proteins [24]. As shown in Fig. 6E, Src phosphorylated p110α in a time-dependent manner (Fig. 6E). Saracatinib, a Src inhibitor, dramatically reduced p110α phosphorylation by Src proteins (Fig. 6F). Saracatinib treatment further reduced phosphorylation of MLC2 (the downstream of p-p110α Y317) in p110α Y508F KI clones and phosphorylation of AKT (the downstream of p-p110α Y508) in p110α Y317F KI clones (Fig. 6G), suggesting that Src might influence both Y317 and Y508 phosphorylation. Moreover, CRC patients with high Src levels showed worse overall survival (Fig. 6H and 6I), suggesting that potential correlations between p-p110α and clinical characteristics.

## Discussion

Although it is well-known that post-translational modifications especially protein phosphorylation are essential for signaling transduction of PI3K pathway, whether protein phosphorylation occurs on PI3K catalytic subunit PIK3CA/p110α, one of the most frequently mutated oncogenes in human cancers, is still an open question. In this study, we identified two tyrosine sites on PIK3CA/p110α and demonstrated their function and underlying mechanisms in CRC.

We clarified that PIK3CA/p110α underwent tyrosine phosphorylation. PI3Kα plays important role in tumorigenesis. Several tyrosine phosphorylation sites have been characterized in the regulatory subunits of PI3Kα, such as p85α and p85β. However, post-translational modifications on p110α are not well documented. In our data, p110α tyrosine phosphorylation was detected by a phospho-tyrosine monoclonal antibody pY-100. Moreover, p110α tyrosine phosphorylation could be stimulated by growth factors, such as EGF, HGF, and PDGF, suggesting their physiological relevance. With targeted phosphoproteomics approach, two tyrosine phosphorylation sites of p110α, Y317 and Y508, were identified. Indeed, several large-scale non-targeted phosphoproteomics data confirmed the existence of tyrosine phosphorylation of p110α. For examples, p110α Y508 phosphorylation has been found in HeLa cells and sarcoma cell lines after immunoaffinity enrichment of Tyr phosphorylated peptides [25, 26]. p110α Y317 phosphorylation was identified in neuroblastoma cell lines and T cells [27, 28]. Arneja et al. discovered that cytokines IL2 and IL15 stimulated p110α phosphorylation at Y317 [28], suggesting that p110α phosphorylation might transduce signaling in different cells or under different physiological conditions.

We demonstrated that phosphorylation of p110α Y317 and Y508 were critical for CRC tumorigenesis. Although phosphoproteomics data uncovered that p110α had tyrosine phosphorylation, the function of p110α phosphorylation have not been studied yet. It is well-known that PIK3CA/p110α play oncogenic role in human cancer. Thus, we generated tyrosine sites mutation knocked-in isogenic cell clones to evaluate their function in tumorigenesis. The data showed that phosphorylation at either Y317 or Y508 was crucial for CRC progression. Interestingly, p110α Y317F mutation showed moderate or even inconsistent effect on proliferation, but significant and uniform inhibitory effect on tumor metastasis, suggesting that phosphorylation at p110α Y317 has a greater impact on tumor metastasis processes. This phenomenon might be explained by p110α Y317 phosphorylation-mediated downstream signaling, Src-MLC2 pathway. Src is a non-receptor tyrosine kinase that influences the migration and invasion of cancer cells by activating several signaling cascades on cytoskeletal recombination and cell adhesion [29]. p-MLC2 (Myosin light chain-2) regulates actomyosin contractility and cell polarity, which are important for tumor metastasis by influencing immunomodulatory secretome and stiffness of metastatic niches [30–32]. MLC2 was phosphorylated by both ROCK and MLC kinase (MLCK) and dephosphorylated by MLC phosphatase (MLCP) [30]. Src affects MLC2 phosphorylation through regulating Rho activity or MLCK phosphorylation [33, 34]. It was shown that p110α Y317F mutation influenced phosphorylation of Src and MLC2. However, we didn’t find that Y317F mutation affected MLCK phosphorylation (data not shown), suggesting that Src affect MLC2 phosphorylation through an unknown mechanism in CRC. Unlike p110α Y317, p110α Y508F mutation abolished AKT phosphorylation, suggesting that p110α Y508 phosphorylation might play important role in regulating classic PI3K activity.

Src phosphorylates PI3K regulatory subunit p85 at tyrosine 688 [13] and class III PI3K VPS34 at Y231 and Y310 [35]. However, whether Src phosphorylates p110α is unknown. In this study, we had some preliminary data to prove that Src phosphorylated p110α. Firstly, Src, but not other Src family proteins, directly interacted with p110α independent of p85. Secondly, mutation on Y317 or Y508 of p110α dramatically abolished p110α-Src interaction, as well as tyrosine phosphorylation of p110α. Thirdly, phospho-tag SDS-PAGE showed that p110α was phosphorylated by Src in a time-dependent manner, and Src inhibitor significantly blocked this phosphorylation process. Surprisingly, p110α showed autophosphorylation activity (Fig. 6F). It has been reported that PI3K had serine kinase activity. Vanhaesebroeck et al. showed that p110δ autophosphorylated at C-terminus of p110δ Ser1039 [36]. Autophosphorylation sites were mapped to C-terminal serine residues of p110β S1070 and p110γ S1101 [37]. It is possible that p110α autophosphorylates its serine sites and this autophosphorylation has been detected by our phospho-tag methods. Notably, Y508F mutation showed more effect on p110α-Src interaction but less effect on p110α tyrosine phosphorylation compared with Y317F mutation. Thus, at this stage, it is still unclear whether Src phosphorylates both Y317 and Y508, or utilizes one tyrosine residue as a docking site and then phosphorylates another one. Phosphorylation site-specific antibodies will help us to address this question in the future investigation.

Unfortunately, couple commercial p-p110α Y317 antibodies and our customized p-p110α Y317 or p-p110α Y508 antibodies were failed to specifically recognize p-p110α protein. High-quality phospho-p110α antibodies will not only help us to understand the signaling transduction pathway of phospho-p110α but also have potentially clinical application.

## Conclusions

**Fig. 7:**
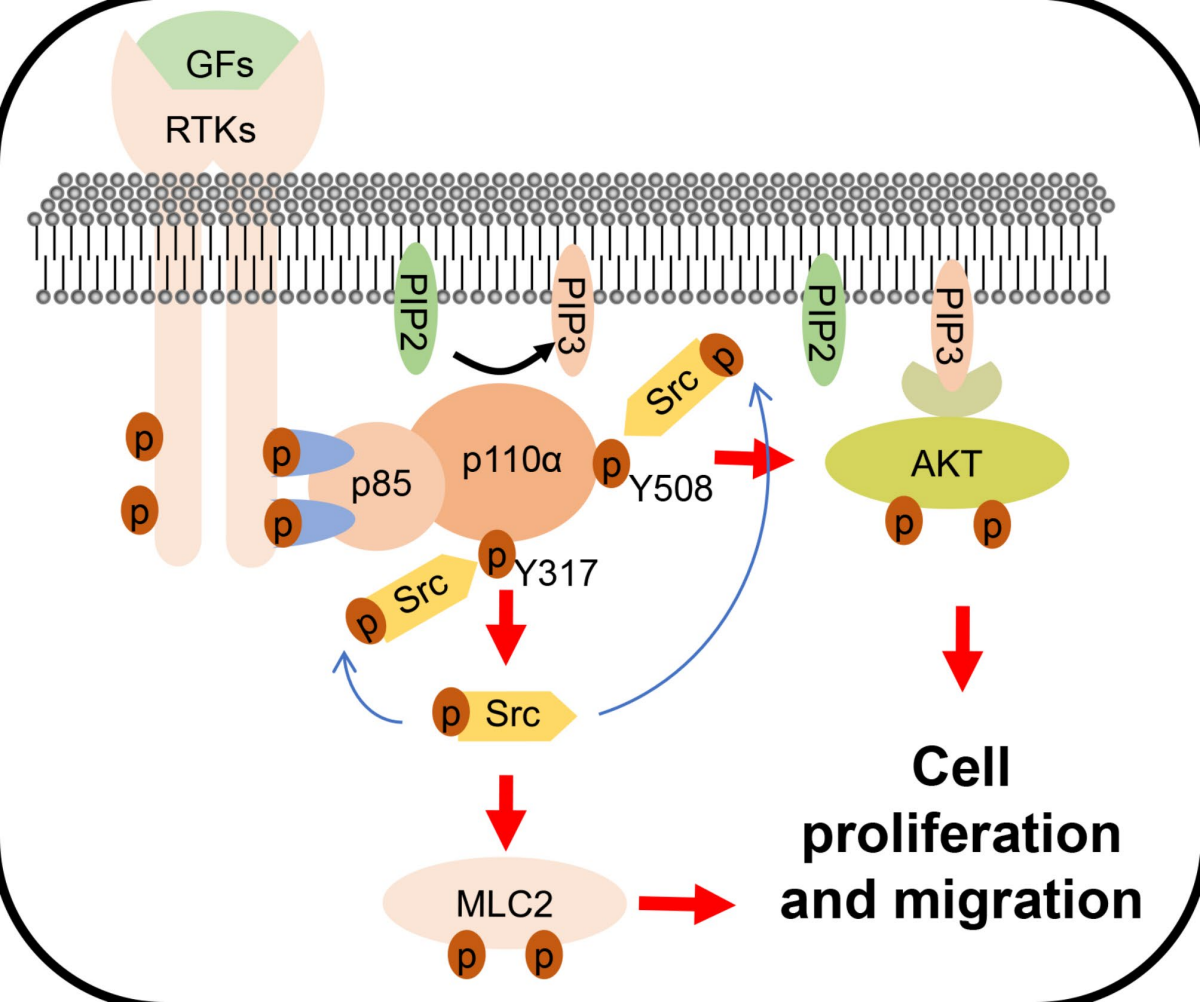
Schematic diagram of phosphorylation at tyrosine 317 and 508 of p110α promoting the growth and metastasis of CRC cells by activating different signaling

In this study, we identify two tyrosine residues on PIK3CA/p110α that play important role in tumorigenesis of CRC. p110α Y317 phosphorylation influences Src-MLC2 pathway, whereas p110α Y508 phosphorylation regulates AKT signaling. Furthermore, Src is a potential tyrosine kinase for p110α. This study could broaden our understanding of the mechanisms underlying PIK3CA/p110α for CRC progression (Fig. 7).

## Materials and methods

### Cell culture

Colorectal cancer cell lines (DLD1, HCT116, SW480, LoVo, RKO, and genetically engineered CRC cell lines) were maintained in McCoy’s 5 A medium (Gibco, cat#16,600,082). Human embryonic kidney HEK 293T cells were cultured in Dulbecco’s modified Eagle’s medium (DMEM, Sigma, cat#D0819). The medium was supplied with 10% of fetal bovine serum (FBS, Gibco, cat#10,099,141 C) and 100 U/mL penicillin plus 100 μg/mL streptomycin (Pen Strep, Gibco, cat# 15,140). Cells were incubated at 37℃ in a humidified atmosphere with 5% CO_2_. All cell lines were authenticated by Shanghai Biowing Biotech (Shanghai, China) using STR profiling and tested routinely to avoid Mycoplasma contamination (cat # 40601ES20, Yeason, Shanghai, China).

### CRISPR/CAS9 genome editing

DLD1 cells and HCT116 cells were endogenously tagged with SBP-3×FLAG-6×HIS at C-terminal of PIK3CA locus as described previously [23]. Briefly, the homology arms were PCR out and cloned into pAAV-loxP-Neo vector. Adeno-associated virus were generated as described previously [38]. DLD1 cells and HCT116 cells were infected with rAAV virus. Stable G418-resistent clones were then selected for PCR screening. Targeted clones were genotyped by RT-PCR and sequenced when necessary. Primers for targeting vector were listed in Table S3.

For CRISPR/CAS9-mediated PIK3CA/p110α Y317F or Y508F endogenous point mutation, guiding RNA in introns surrounding mutation sites were designed with IDT design tool (https://sg.idtdna.com/pages) and cloned individually into pX330 vector. Homologous arms of PIK3CA genome sequence containing Y317F or Y508F point mutations were cloned into the pAAV-loxP-Neo vector. Targeting vectors were co-transfected with gRNA vectors into indicated cell lines. G418-resistant clones were screened by genomic PCR and verified by cDNA sequencing. The primers for vector construction and PCR are listed in Table S3.

### Reagents, DNA constructs and mutagenesis

EGF (cat# 8916) and FGF (cat# 61,977) were purchased from cell signaling technology (MA, USA). PDGF-BB (cat# 100-14B) was purchased from Peprotech (NJ, USA). HGF (cat# HZ-1084) was purchased from Proteintech (IL, USA). Insulin (cat# I9278) and Sodium orthovanadate (cat# 450,243) were obtained from Sigma (Merck, Germany). Src inhibitor Saracatinib (cat# S1006) was purchased from Selleck (TX, USA). pCMV backbones (Invitrogen) were used for gene expression in mammalian cells. PIK3CA/p110α was cloned into pCMV-3×FLAG vector. Src, Fyn, and Lyn were cloned into pCMV-3×Myc vector. Point mutations of Y317F, Y508F, or Y317F/Y508F on PIK3CA/p110α were generated with QuikChange Site-Directed Mutagenesis Kit (Agilent, cat #200,518). MLC2 and myr-AKT constructs were purchased from GeneCopoeia (Guangzhou, China). The primers using for constructs cloning were listed in Table S3.

### RNA extraction and qRT-PCR

Total RNA was extracted using the TRIzol kit (Invitrogen, cat#15,596,026). 1 μg of total RNA was reverse-transcribed into cDNA using Prime-Script RT kit (Takara, cat#RR037B, Japan) according to the manufacturer’s instructions. Y317F or Y508F KI mutant colons were sequenced with primers surrounding Y317 or Y508 sites. The gene expression levels were measured by qRT-PCR with SYBR Premix EX Tag (Takara, cat#RR820A, Japan). Each experiment was performed in triplicate. Objective CT values were normalized to internal reference gene β-actin and 2^−ΔCt^ was used to calculate relative mRNA levels. Primers were listed in Table S3.

### Immunoblotting and immunoprecipitation

Immunoprecipitation and immunoblotting were performed as described previously [39]. Briefly, cells were lysed in RIPA lysis buffer (Invitrogen, cat#89,900) supplemented with complete proteinase inhibitor Cocktail (Roche, cat#11,836,170,001, Switzerland) and phosphatase inhibitor PhosSTOP (Roche, cat#4,906,845,001). Cell lysates were boiled and dissolved in SDS-PAGE gels, then blotted with antibodies. The antibodies used in this study were listed in Table S4.

For transfection-based immunoprecipitation assays, cells were transfected with indicated vectors and lysed in 1 mL of RIPA lysis buffer supplemented with complete protease inhibitor, PhosSTOP, and tyrosine phosphatase inhibitors (1mM Na_3_VO_4_, 50mM NaF). Cell lysates were immunoprecipitated with indicated agarose beads at 4℃ for 3 h. The beads were washed three times and boiled with SDS loading buffer. The antibodies-conjugated agarose beads were listed in Table S4.

### In vitro kinase assay and Phos-tag SDS/PAGE

6×His tagged PIK3CA/p110α and p85α were cloned into baculoviral vector pFastBac (Invitrogen). Baculovirus expressing 6×His tagged PIK3CA/p110α or p85α were prepared according to Bac-to-Bac baculovirus expression system (Invitrogen, USA). Sf-9 cell were infected with baculovirus of both 6×His tagged PIK3CA/p110α and p85α, and recombinant p110α were purified with Ni-NTA agarose as described previously [23]. Src was cloned into pET-28a vector, and then transformed into BL21(DE3) competent E. coli cells. Recombinant Src proteins were purified with Ni-NTA agarose according to Qiagen handbook. Purified recombinant proteins were dialyzed with slide-A-lyzer dialysis cassettes (ThermoFisher, USA).

For in vitro kinase assay, 50 ng of purified 6×His-p110α/p85α complex proteins, and purified 6×His-Src proteins were incubated in 25 μl of 1×tyrosine kinase buffer (Sigma, cat# PTK101) supplemented with 0.32 mM ATP at 37℃. After indicated times, the reactions were terminated by adding SDS loading buffer and boiling at 100℃ for 5 min. Phos-tag SDS/PAGE was made with 5 mM Phos-tag (FUJIFILM Wako Chemicals, cat# AAL-107, VA, USA), 10 mM MnCl_2,_ and 7.5% normal SDS/PAGE solution (avoiding light). The samples were then dissolved in Phos-tag SDS/PAGE and electrophoresed at 30 mA for 3.5 h. Proteins were electrophoretically transferred to PVDF membrane at 15 mA in 4 degree overnight, then followed by regular immunoblotting.

### Cell proliferation assay

In vitro proliferation of CRC cells was measured using Cell Counting Kit-8 (Dojindo, Japan). Briefly, 2000 cells were seeded in 96-well plates and cultured for 5 days in 100 μl McCoy’s 5 A medium supplemented with 10% FBS. According to the manufacturer’s instructions, 10 μl per well of CCK-8 solution was added, and the absorbance values at 450 nm were measured for 5 consecutive days.

### Cell migration and invasion assay

Cell migration assays were conducted by 24-well plates and transwell filter chambers (BD Biosciences, USA). 5 × 10^4^ CRC cells in 200 μl McCoy’s 5 A serum-free medium were seeded in the upper chamber, and 800 μl McCoy’s 5 A medium supplemented with 10% FBS was added into the lower chamber of the 24-well plate. After 48 h incubation, cells migrated through the membrane were fixed and stained by crystal violet. Average colony numbers were counted with randomized 3 ~ 5 images under microscope.

For Cell invasion assays, a matrigel chamber (BD Biosciences) were applied. 8 × 10^4^ CRC cells in 200 μl serum-free McCoy’s 5 A were seeded in the upper chamber. The following steps were similar as described above.

### Wound healing assay

Cells were grown in 12-well plates at 95% confluency. The linear wounds were scratched via a 200 μl sterile pipette tip. After washing with PBS to remove cell debris, adherent cells were incubated in medium with 10% FBS. The migrating distance were photographed every 3 h for 24 h.

### Xenograft models

All animal experiments were performed in accordance with protocols approved by the IACUC committee at Shanghai Medical Experimental Animal Care Commission. Subcutaneous xenograft tumor models were established as described previously [40]. Briefly, two million cells were injected subcutaneously and bilaterally into athymic nude mice. Tumor volumes were measured twice a week and calculated with the formula of (length × width^2^)/2.

For in vivo tumor metastasis experiment, three million cells in 100 μL of serum-free McCoy’s 5 A were injected into nude mice through tail vein. Two to three months later, mice were sacrificed and dissected. The metastatic nodules in the lungs were counted and then stained with hematoxylin and eosin.

### Statistical analysis

All statical analyses were done using GraphPad Prism 5.0 software. Results were presented as mean ± standard deviation (SD, n = 3). The student’s *t* test (paired/unpaired) were used for statistical differences between groups, and Two-way analysis of variance (ANOVA) was used for comparing significant difference of multiple groups. P < 0.05 was considered statistically different (*P < 0.05, **P < 0.01, ***P < 0.001, ****P < 0.0001), otherwise not significant (n.s.).

### Electronic supplementary material

Below is the link to the electronic supplementary material.

Supplementary Material 1

## Data Availability

Data sets used and/or analyzed during the current study are available from the corresponding author upon reasonable request.
