# Supplementary material for: Phosphorylation at tyrosine 317 and 508 are crucial for PIK3CA/p110α to promote CRC tumorigenesis
Source: Cell Biosci. 2023 Sep 9;13:164. doi: 10.1186/s13578-023-01102-7 (PMC10493024; doi:10.1186/s13578-023-01102-7)
Supplement: Supplementary file 1 — Supplementary Material 1 [file 13578_2023_1102_MOESM1_ESM.docx]

**Phosphorylation at tyrosine 317 and 508 are crucial for PIK3CA/p110α to promote CRC tumorigenesis**

**Table of Contents**

**Supplementary Figures**

Figure S1 Genetically engineered PIK3CA/p110α Y317F or Y508F knock-in mutant isogenic cell lines.

Figure S2 p110α Y317F mutation impairs the proliferation, migration, and invasion of CRC cells.

Figure S3 p110α Y508F heterozygous mutation impairs the proliferation, migration, and invasion of CRC cells.

Figure S4 p110α Y317F mutation attenuates SRC-MLC2 signaling pathway. **A-B** p110α Y317F mutation didn’t alter AKT signaling pathway.

Figure S5 p110α Y508F heterozygous mutation didn’t alter Src signaling and EMT pathway.

Figure S6 Src directly interacts with p110α.

**Supplementary tables**

Supplementary Table S1. PTMs of PIK3CA/p110α and its associated proteins by mass spectrometry.

Supplementary Table S2. Phospho-proteomics analysis.

Supplementary Table S3. List of primers.

Supplementary Table S4. List of antibodies.

**
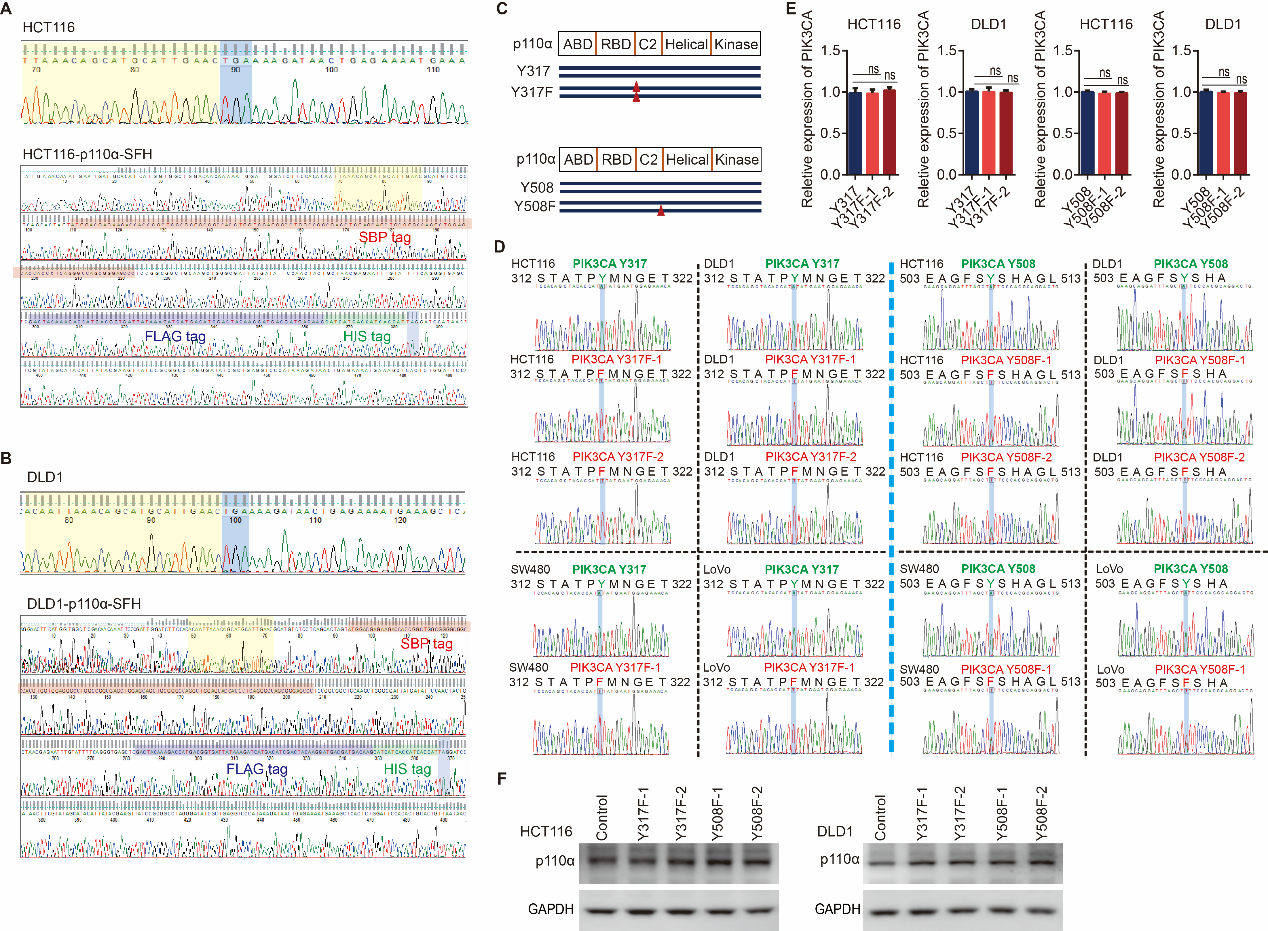
Fig. S1** Genetically engineered PIK3CA/p110α Y317F or Y508F knock-in mutant isogenic cell lines. **A-B** Sequencing of SBP-FLAG-HIS tag in HCT116-p110α-SFH knock-in cell clone (**A**) and DLD1-p110α-SFH knock-in cell clone (**B**). **C** Schematics of p110α Y317F or Y508F knock-in (KI) mutant cell lines. Indicated cells were endogenously knocked in Y317 to F mutation (exon 4) or Y508 to F mutation (exon 8) on PIK3CA locus. The p110α Y317F isogenic KI mutant cell clones were homozygous and the p110α Y508F isogenic KI mutant cell clones were heterozygous. Homozygous of p110α Y508F mutant clones were not able to generate. **D** Sequencing of p110α Y317F or Y508 KI mutations. RNA of parental cells and isogenic mutation KI cell clones was extracted and reverse transcribed into cDNA. The Sequencing of parental cells and individual p110α Y317F or Y508F KI mutant clones were present. Blue boxes indicated the mutant nucleotides. Mutant amino acids and their wild-type counterparts were labeled in the red and green respectively. **E-F** p110α Y317F or Y508F mutations didn’t affect PIK3CA/p110α expression. The mRNA (**E**) and protein (**F**) levels of PIK3CA/p110α in indicated cell lines were quantified with qRT-PCR and Western blots respectively. ns, not significant, two-tailed unpaired *t* test.

**
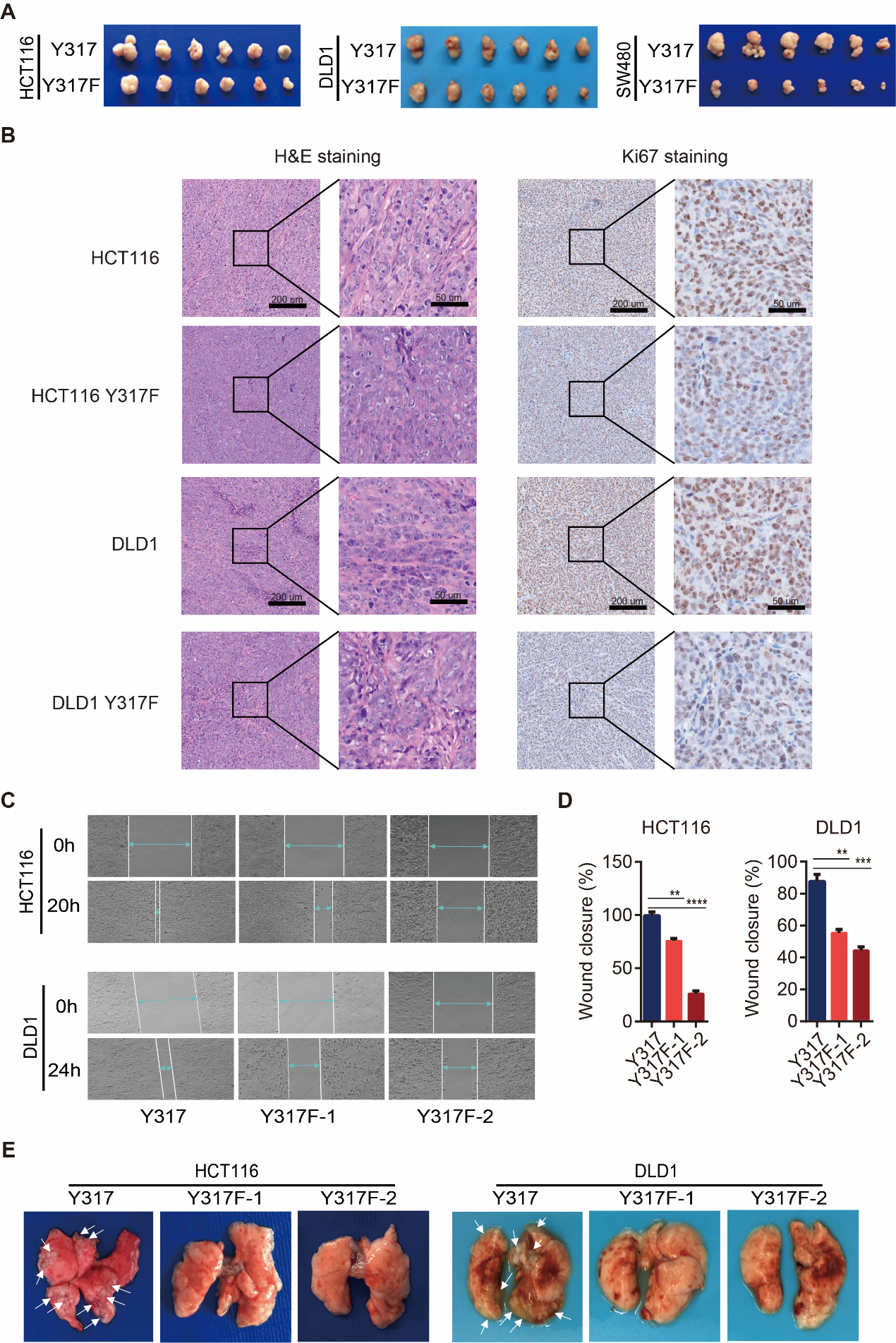
Fig. S2** p110α Y317F mutation impairs the proliferation, migration, and invasion of CRC cells. **A** The images of xenograft tumors (related to Fig. 2D). **B** The images of H&E staining and Ki67 IHC staining of parental cells and Y317F KI mutant clones. **C-D** p110α Y317F mutation inhibited cell migration by wound healing assay. Two-tailed unpaired *t* test, **P < 0.01, ***P < 0.001, ****P < 0.0001. **E** Representative images of lung tissues from the nude mice injected with indicated cell lines. White arrows indicated metastatic nodules on the lungs.

**
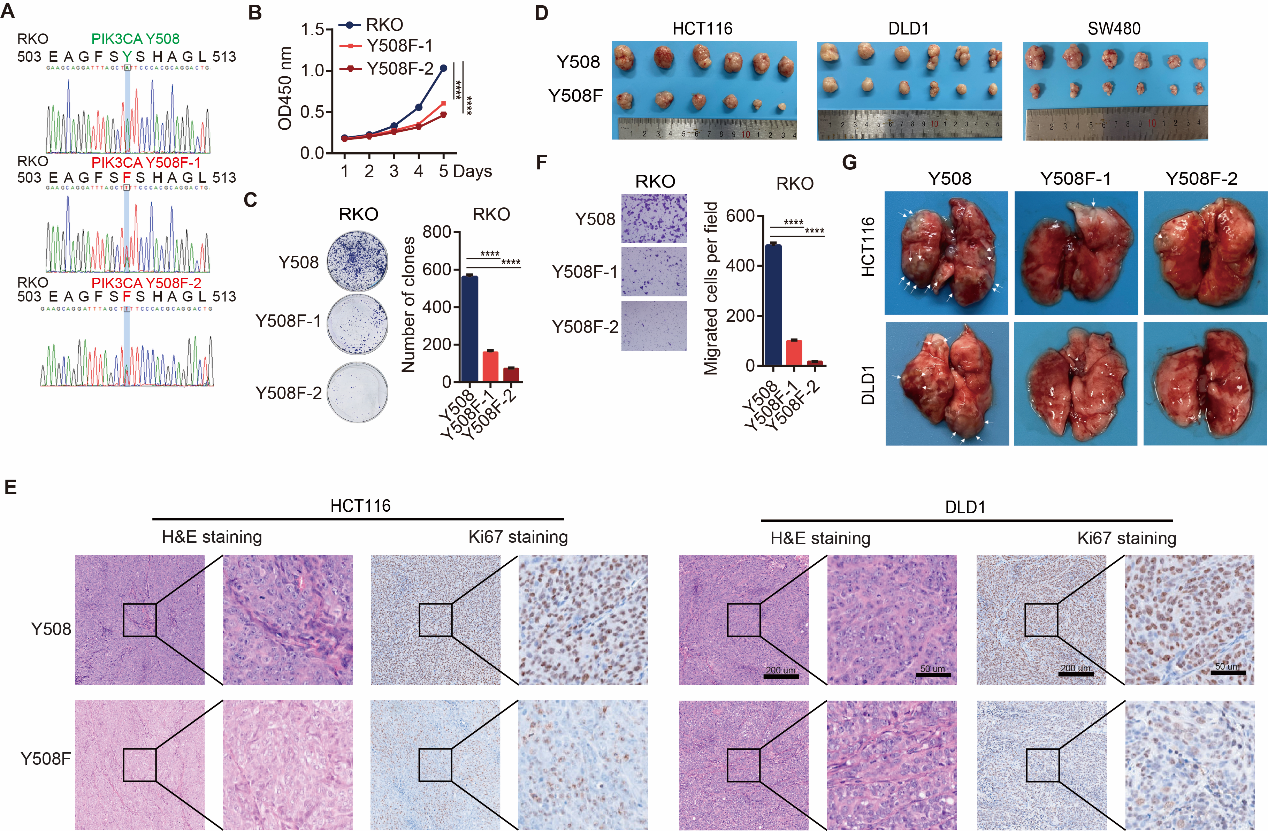
Fig. S3** p110α Y508F heterozygous mutation impairs the proliferation, migration, and invasion of CRC cells. **A** Sequencing of RKO parental cells and isogenic p110α Y508F KI mutant cells. **B-C** RKO parental cells and isogenic p110α Y508F KI mutant cells were analyzed for cell proliferation (**B**) and colony formation (**C**). **D** The images of xenograft tumors from indicated cell lines (related to Fig. 3D). **E** The images of H&E staining and Ki67 IHC staining of parental cells and Y508F KI mutant clones. **F** The migration abilities of RKO parental cells and isogenic p110α Y508F KI mutant cells were analyzed by transwell assays. **G** Representative images of lung tissues from nude mice injected with indicated cell lines. White arrows indicated metastatic foci of the lungs.


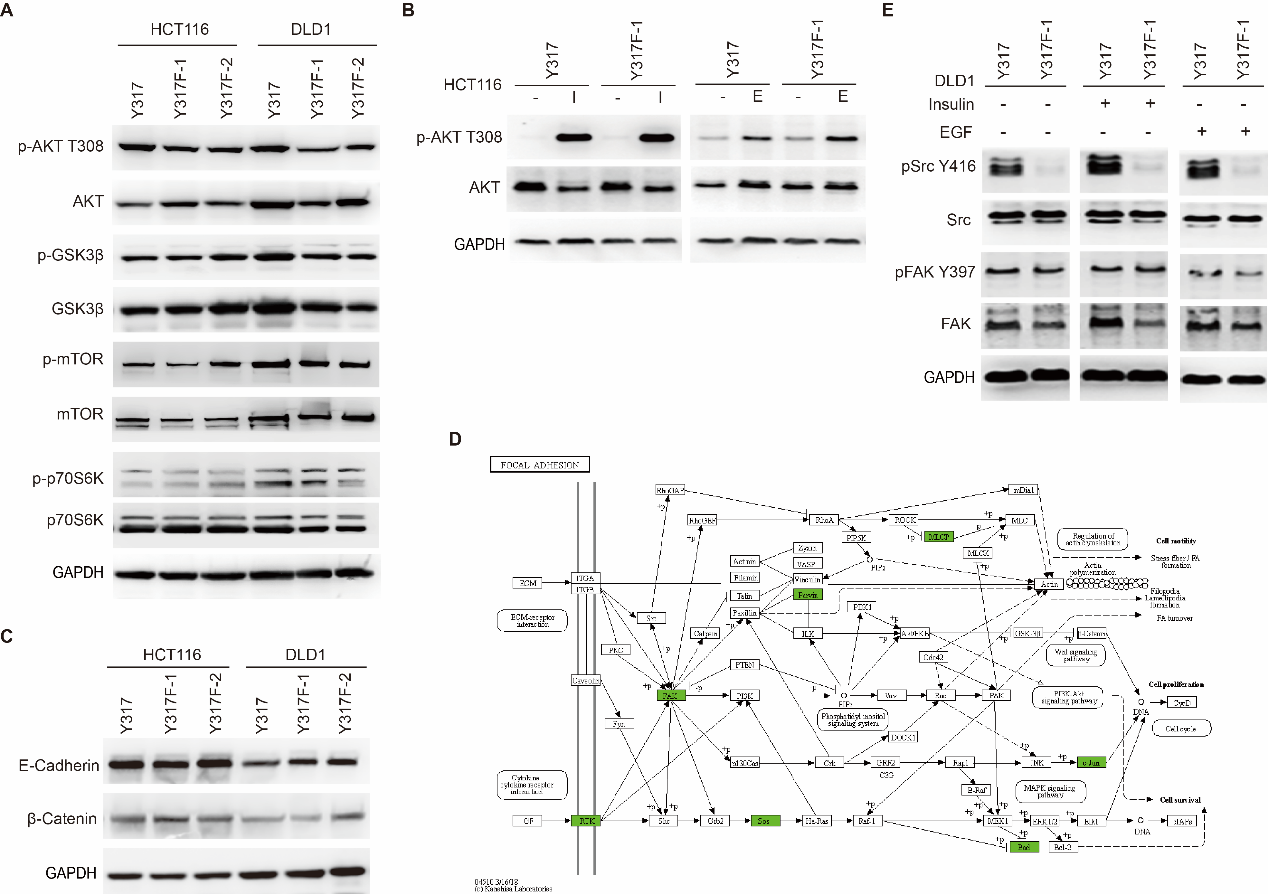
**Fig. S4** p110α Y317F mutation attenuates SRC-MLC2 signaling pathway. **A-B** p110α Y317F mutation didn’t alter AKT signaling pathway. Parental cells and their corresponding p110α Y317F KI mutant cells were serum-starved overnight (**A**) or followed with insulin (I) or EGF (E) treatment (**B**). Cell lysates were collected and analyzed by Western blots with indicated antibodies. **C** p110α Y317F mutation didn’t affect EMT pathway. Parental cells and their corresponding p110α Y317F KI mutant cells were lysed and blotted with indicated antibodies. **D** Schematic diagram of focal adhesion pathway by KEGG enrichment analysis with differentially phosphorylated proteins between HCT116 cells and HCT116 p110α Y317F KI mutant cells. The proteins which were less phosphorylated in HCT116 p110α Y317F KI mutant cells compared with HCT116 cells were indicated with green boxes. **E** p110α Y317F mutation impairs Src signaling. DLD1 cells and DLD1 p110α Y317F KI mutant cells were serum-starved overnight and treated with EGF or insulin. Cell lysates were collected and blotted with indicated antibodies.


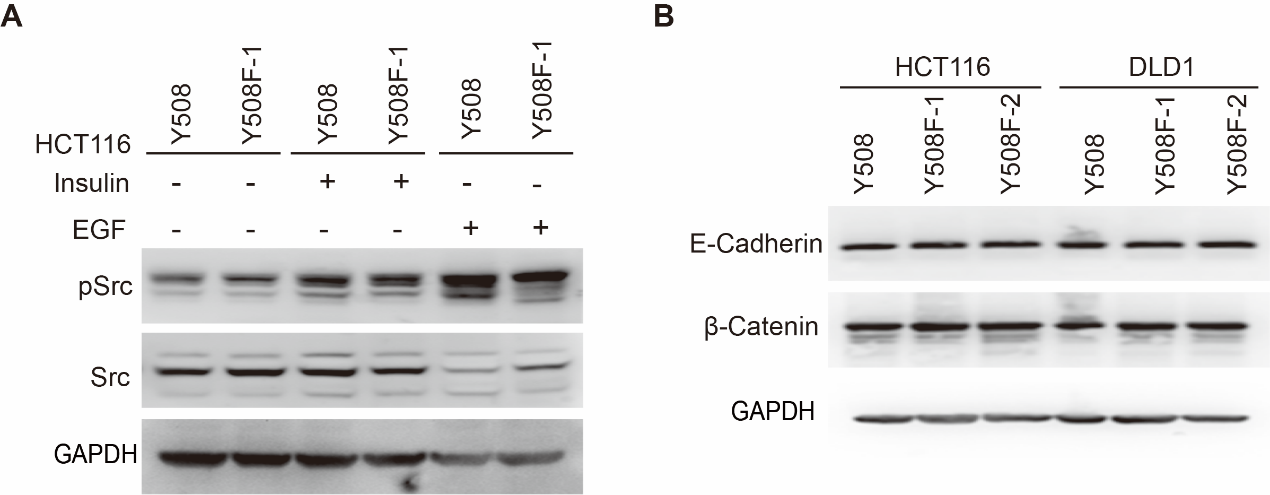
**Fig. S5** p110α Y508F heterozygous mutation didn’t alter Src signaling and EMT pathway. **A** p110α Y508F mutation didn’t affect Src phosphorylation. HCT116 cells and HCT116 p110α Y508F KI mutant cells were serum-starved overnight and then treated with insulin or EGF for 15 mins. Cell lysates were blotted with indicated antibodies. **B** p110α Y508F mutation didn’t impair EMT pathway. Parental cells and their corresponding p110α Y508F KI mutant cells were blotted with indicated antibodies.


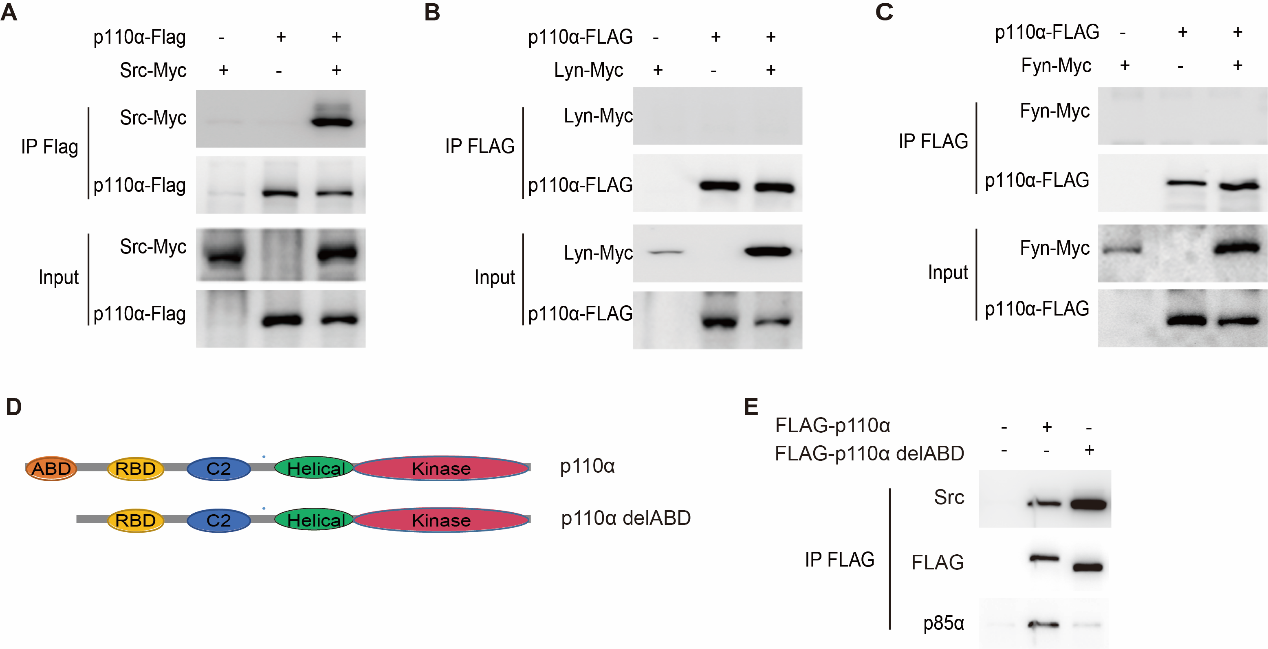
**Fig. S6** Src directly interacts with p110α. **A-C** Flag-tagged p110α was co-transfected with Myc-tagged Src (**A**), Lyn (**B**) or Fyn (**C**) in HEK293T cells. p110α were pulled down with anti-FLAG agarose beads, and immunocomplex were blotted with indicated antibodies. **D** Schematic of p110α delABD construct in which p85 binding domain (ABD domain) was deleted from full-length p110α. **E** FLAG-tagged p110α or p110α delABD was transfected into HEK293T cells. p110α or p110α delABD was pulled down with anti-FLAG agarose beads, then followed by Western blot analysis with indicated antibodies.

| **Table S1. PTMs of PIK3CA/p110α and its associated proteins by mass spectrometry** | | | | | | |
| --- | --- | --- | --- | --- | --- | --- |
| Protein | | Identified PTM-containing Peptides | | | | |
| Gene annotations | SC (%) | position | Error (ppm) | Sequnce | PTM | Ions score |
| PIK3CA_HUMAN | 76 | 311-325 | -2 | R.ISTATP**Y**MNGETSTK.S | Phos(Y) | 42 |
|  |  | 503-516 | -4 | R.EAGFS**Y**SHAGLSNR.L | Phos(Y) | 26 |
| p85β_HUMAN | 70 | 257-286 | -2 | R.APPPPS**S**PPPGGAPDGSEPSPDFPALLVEK.L | Phos(ST) | 94 |
| IRS1_HUMAN | 61 | 268-285 | -3 | R.SK**S**QSSSNCSNPISVPLR.R | Phos(ST) | 38 |
|  |  | 270-285 | -3 | K.**S**QSSSNCSNPISVPLR.R | Carb.(C) | 46 |
|  |  | 303-325 | -2 | R.SRTESITATSPASMVGGKPG**S**FR.V | Phos(ST) | 74 |
|  |  | 305-325 | -3 | R.TE**S**ITATSPASMVGGKPGSFR.V | Phos(ST) | 37 |
|  |  | 326-342 | -2 | R.VRA**S**SDGEGTMSRPASV.D | Phos(ST) | 46 |
|  |  | 326-353 | -3 | R.VRASSDGEGTM**S**RPA**S**VDGSPVSPSTNR.T | 2Phos(ST) | 32 |
|  |  | 328-342 | -3 | R.AS**S**DGEGTMSRPASV.D | Phos(ST) | 74 |
|  |  | 328-353 | -2 | R.A**S**SDGEGTMSRPASVDGSPVSPS**T**NR.T | Oxid.(M), 2 Phos(ST) | 79 |
|  |  | 339-353 | -3 | R.PASVDGSPV**S**PSTNR.T | Phos(ST) | 77 |
|  |  | 339-353 | -3 | R.PA**S**VDGSPV**S**PSTNR.T | 2 Phos(ST) | 37 |
|  |  | 382-410 | -3 | R.CSPSATSPVSL**S**SSSTSGHGSTSDCLFPR.R | Phos(ST) | 41 |
|  |  | 382-410 | -1 | R.CSPSAT**S**PVSLSSSSTSGHGSTSDCLFPR.R | Phos(ST) | 78 |
|  |  | 382-410 | -2 | R.CSPSAT**S**PV**S**LSSSSTSGHGSTSDCLFPR.R | 2 Phos(ST) | 43 |
|  |  | 444-457 | -3 | R.SVTPDSLGH**T**PPAR.G | Phos(ST) | 58 |
|  |  | 444-457 | -4 | R.SV**T**PDSLGH**T**PPAR.G | 2 Phos(ST) | 33 |
|  |  | 458-471 | -4 | R.GEEELSN**Y**ICMGGK.G | Phos(Y) | 111 |
|  |  | 596-626 | -3 | R.GGHHRPDSSTLHTDDG**Y**MPM**S**PGVAPVPSGR.K | Phos(Y), Phos(ST) | 36 |
|  |  | 627-638 | -4 | R.KG**S**GDYMPM**S**PK.S | 2 Phos(ST) | 37 |
|  |  | 627-638 | -3 | R.KG**S**GD**Y**MPM**S**PK.S | 2 Phos(ST), Phos(Y) | 36 |
|  |  | 1141-1161 | -2 | R.HS**S**ASFENVWLRPGELGGAPK.E | Phos(ST) | 45 |
|  |  | 1166-1189 | -3 | K.LCGAAGGLENGLN**Y**IDLDLVKDFK.Q | Phos(Y) | 43 |
| ERBB3_HUMAN | 15 | 684-695 | -4 | R.GE**S**IEPLDPSEK.A | Phos(ST) | 36 |
| IRS2_HUMAN | 6 | 182-195 | -2 | E.DS**Y**GLVAPATAAYR.E | Phos(Y) | 56 |
|  |  | 363-381 | -3 | R.TA**S**EGDGGAAAGAAAAGAR.P | Phos(ST) | 111 |
|  |  | 1184-1205 | -4 | R.K**S**SEGGVGVGPGGGDEPPT**S**PR.Q | 2 Phos(ST) | 56 |
| P55G_HUMAN | 17 | 34-72 | -11 | D.PPALPP**K**PP**K**PMTSAVPNGMKDSSVSLQDAEWYWGDISR.E | Ac(K), Me(K) | 51 |
| SC(%): sequence coverage | | | | | | |

**Table S2. Phospho-proteomics analysis**

| Protein | Abbreviated name | Phosphorylation site | Peptide Sequence | m/z | Charge | MS/MS Count | HCT116_1 | HCT116_2 | H_Y317F1 | H_Y317F2 | H_Y317F/HCT116 Ratio | Regulated Type | KOG category | KOG classify |
| --- | --- | --- | --- | --- | --- | --- | --- | --- | --- | --- | --- | --- | --- | --- |
| [F-actin]-monooxygenase MICAL1 | MICAL1 | s617 | SMAHsPGPVSQASPGTSSAVLFLSK | 841.7365 | 3 | 9 | 1.401 | 1.488 | 1.083 | 1.078 | 0.7485 | Down | TZ | [T] Signal transduction mechanisms [Z] Cytoskeleton |
| 5'-3' exoribonuclease 2 | XRN2 | s471 | MQNNSsPSISPNTSFTSDGSPSPLGGIK | 989.7541 | 3 | 8 | 0.731 | 0.77 | 0.484 | 0.522 | 0.669 | Down | LA | [L] Replication, recombination and repair [A] RNA processing and modification |
|  |  | s475 | MQNNSSPSIsPNTSFTSDGSPSPLGGIK | 963.0987 | 3 | 12 | 0.459 | 0.483 | 0.304 | 0.327 | 0.669 | Down | LA | [L] Replication, recombination and repair [A] RNA processing and modification |
| Activating transcription factor 7-interacting protein 1 | ATF7IP | s673 | RHEHPPNPPVsPGK | N/A | 3 | 23 | 1.633 | 1.512 | 1.032 | 0.998 | 0.646 | Down | N/A | N/A |
| Afadin- and alpha-actinin-binding protein | SSX2IP | s312 | VDDSTGTVIsDVEEDAGELSR | 1137.486 | 2 | 8 | 1.344 | 1.343 | 0.894 | 1.004 | 0.706 | Down | N/A | N/A |
| Afadin | AFDN | s1275 | sQEELREDK | N/A | 2 | 27 | 1.042 | 0.759 | 0.725 | 0.57 | 0.7235 | Down | Z | [Z] Cytoskeleton |
| A-kinase anchor protein 11 | AKAP11 | s1171 | SLsEEVESSESGELPEVDVK | 1114.988 | 2 | 7 | 1.742 | 1.729 | 1.167 | 1.361 | 0.729 | Down | N/A | N/A |
| A-kinase anchor protein 12 | AKAP12 | s381 | LSAEYEKVELPsEEQVSGSQGPSEEKPAPLATEVFDEK | 1054.245 | 4 | 13 | 1.239 | 1.174 | 0.933 | 0.734 | 0.689 | Down | N/A | N/A |
|  |  | s483 | LKETCVSGEDPTQGADLsPDEK | 1228.53 | 2 | 9 | 1.291 | 1.169 | 0.874 | 0.748 | 0.6585 | Down | N/A | N/A |
|  |  | s505 | VLSKPPEGVVSEVEMLSsQER | 794.0537 | 3 | 9 | 1.323 | 1.23 | 0.729 | 0.796 | 0.5995 | Down | N/A | N/A |
|  |  | s612 | EGVTPWAsFKK | 665.3156 | 2 | 7 | 1.078 | 1.388 | 0.705 | 0.901 | 0.652 | Down | N/A | N/A |
| A-kinase anchor protein 13 | AKAP13 | t2467 | RAEtFGGFDSHQMNASK | 654.9437 | 3 | 10 | 1.439 | 1.469 | 0.963 | 0.934 | 0.6525 | Down | T | [T] Signal transduction mechanisms |
| Alpha-parvin | PARVA | s8 | ATSPQKsPSVPKSPTPK | 969.9523 | 2 | 19 | 0.935 | 0.739 | 0.348 | 0.276 | 0.374 | Down | Z | [Z] Cytoskeleton |
| Anaphase-promoting complex subunit 1 | ANAPC1 | s1347 | HKsPSYQIK | 584.2815 | 2 | 11 | 1.5 | 1.17 | 1.093 | 0.838 | 0.7225 | Down | DO | [D] Cell cycle control, cell division, chromosome partitioning [O] Posttranslational modification, protein turnover, chaperones |
| Ankyrin repeat and zinc finger domain-containing protein 1 | ANKZF1 | s675 | LAAQLGAPTsPIPDSAIVNTR | 1086.557 | 2 | 7 | 1.508 | 1.31 | 1.034 | 1.03 | 0.736 | Down | R | [R] General function prediction only |
| Annexin A2 | ANXA2 | s12 | LsLEGDHSTPPSAYGSVK | 962.938 | 2 | 17 | 1.776 | 1.755 | 1.294 | 1.4 | 0.763 | Down | U | [U] Intracellular trafficking, secretion, and vesicular transport |
|  |  | s127 | GLGTDEDsLIEIICSR | 929.4186 | 2 | 6 | 1.113 | 1.305 | 0.836 | 0.882 | 0.713 | Down | U | [U] Intracellular trafficking, secretion, and vesicular transport |
| Arf-GAP domain and FG repeat-containing protein 1 | AGFG1 | t177 | SLLGDSAPTLHLNKGtPSQSPVVGR | 897.7683 | 3 | 8 | 1.334 | 1.878 | 1.079 | 1.357 | 0.7655 | Down | T | [T] Signal transduction mechanisms |
|  |  | s181 | SLLGDSAPTLHLNKGTPSQsPVVGR | 897.7683 | 3 | 7 | 1.241 | 1.748 | 1.004 | 1.264 | 0.7655 | Down | T | [T] Signal transduction mechanisms |
| Arf-GAP with coiled-coil, ANK repeat and PH domain-containing protein 2 | ACAP2 | s521 | YSISLsPPEQQK | 728.8396 | 2 | 11 | 1.59 | 1.428 | 1.077 | 1.125 | 0.7325 | Down | T | [T] Signal transduction mechanisms |
| Aryl hydrocarbon receptor nuclear translocator | ARNT | s77 | FARsDDEQSSADKER | 910.876 | 2 | 11 | 1.261 | 1.564 | 0.861 | 1.082 | 0.688 | Down | K | [K] Transcription |
| Ataxin-2-like protein | ATXN2L | s496 | SAAPAPISASCPEPPIGSAVPTSSASIPVTSSVSDPGVGSISPAsPK | 1113.794 | 4 | 7 | 1.801 | 1.309 | 0.994 | 0.87 | 0.6085 | Down | A | [A] RNA processing and modification |
| AT-hook DNA-binding motif-containing protein 1 | AHDC1 | s1507 | TEAACLSAPHLAsPPATPK | 999.9714 | 2 | 9 | 1.31 | 1.297 | 0.967 | 0.844 | 0.6935 | Down | N/A | N/A |
| ATPase family AAA domain-containing protein 1 | ATAD1 | s322 | EYVNSTSEEsHDEDEIRPVQQQDLHR | 805.8508 | 4 | 11 | 1.373 | 1.008 | 0.789 | 0.476 | 0.523 | Down | O | [O] Posttranslational modification, protein turnover, chaperones |
| ATP-binding cassette sub-family F member 1 | ABCF1 | t108 | KLSVPtSDEEDEVPAPKPR | N/A | 2 | 48 | 1.078 | 1.006 | 0.644 | 0.724 | 0.658 | Down | J | [J] Translation, ribosomal structure and biogenesis |
| ATP-dependent RNA helicase DDX1 | DDX1 | s481 | DNTRPGANsPEMWSEAIK | 694.9699 | 3 | 16 | 1.018 | 0.927 | 0.755 | 0.679 | 0.736 | Down | A | [A] RNA processing and modification |
| BAG family molecular chaperone regulator 3 | BAG3 | s386 | VPPAPVPCPPPSPGPSAVPSsPK | 767.0446 | 2 | 28 | 1.419 | 1.261 | 1.02 | 1.02 | 0.7645 | Down | O | [O] Posttranslational modification, protein turnover, chaperones |
| B-cell CLL/lymphoma 9 protein | BCL9 | s278 | PAAPPRPLDREsPGVENK | 670.665 | 3 | 9 | 1.781 | 1.597 | 1.148 | 1.159 | 0.6855 | Down | N/A | N/A |
|  |  | s687 | IPVEGPLsPSR | 616.3078 | 2 | 8 | 1.654 | 1.732 | 1.207 | 1.336 | 0.7515 | Down | N/A | N/A |
|  |  | s917 | SPPVLGSAAAsPVHLK | 805.9187 | 2 | 8 | 1.689 | 1.486 | 1.141 | 0.925 | 0.6495 | Down | N/A | N/A |
| Bcl2-associated agonist of cell death | BAD | s118 | RMsDEFVDSFK | 720.7969 | 2 | 12 | 1.59 | 1.34 | 0.584 | 0.571 | 0.3975 | Down | N/A | N/A |
| Bcl-2-like protein 12 | BCL2L12 | s273 | ACPGPPPPsPEPLAR | 811.8735 | 2 | 7 | 1.462 | 1.132 | 1.003 | 0.928 | 0.753 | Down | N/A | N/A |
| Brefeldin A-inhibited guanine nucleotide-exchange protein 1 | ARFGEF1 | s1569 | sVDIHDSIQPR | 673.8086 | 2 | 8 | 1.187 | 1.058 | 0.807 | 0.701 | 0.671 | Down | U | [U] Intracellular trafficking, secretion, and vesicular transport |
| Calcipressin-3 | RCAN3 | s148 | QFLIsPPASPPVGWK | 892.4146 | 2 | 7 | 0.964 | 2.151 | 0.694 | 1.586 | 0.7295 | Down | TR | [T] Signal transduction mechanisms [R] General function prediction only |
| Canalicular multispecific organic anion transporter 1 | ABCC2 | s283 | NQSQsQDALVLEDVEK | 941.9251 | 2 | 4 | 1.215 | 1.264 | 0.807 | 0.792 | 0.6455 | Down | Q | [Q] Secondary metabolites biosynthesis, transport and catabolism |
| Cation-independent mannose-6-phosphate receptor | IGF2R | s2409 | ALSSLHGDDQDsEDEVLTIPEVK | 859.725 | 2 | 18 | 0.831 | 0.786 | 0.608 | 0.611 | 0.756 | Down | TU | [T] Signal transduction mechanisms [U] Intracellular trafficking, secretion, and vesicular transport |
|  |  | s2479 | LVsFHDDSDEDLLHI | 957.8816 | 2 | 8 | 1.23 | 1.368 | 0.778 | 0.725 | 0.5815 | Down | TU | [T] Signal transduction mechanisms [U] Intracellular trafficking, secretion, and vesicular transport |
| Centrosomal protein of 192 kDa | CEP192 | s1755 | GEVISSGSKPLsPGPCLDIPSILSNK | 911.4574 | 3 | 8 | 1.698 | 0.93 | 1.168 | 0.649 | 0.6925 | Down | Z | [Z] Cytoskeleton |
| Chromatin assembly factor 1 subunit A | CHAF1A | s775 | sPSTTYLHTPTPSEDAAIPSK | 1140.525 | 3 | 22 | 1.594 | 1.315 | 0.974 | 0.883 | 0.6415 | Down | B | [B] Chromatin structure and dynamics |
| Chromosome alignment-maintaining phosphoprotein 1 | CHAMP1 | s214 | LAPVPSPEPQKPAPVsPESVK | 1157.56 | 2 | 24 | 1.831 | 0.524 | 1.253 | 0.371 | 0.697 | Down | N/A | N/A |
|  |  | s297 | KPSPSESPEPWKPFPAVsPEPR | 869.3958 | 3 | 19 | 0.89 | 0.769 | 0.552 | 0.564 | 0.676 | Down | N/A | N/A |
|  |  | s432 | KPGPPLSPEIRsPAGSPELR | 775.6862 | 2 | 23 | 1.35 | 1.124 | 0.895 | 0.788 | 0.6815 | Down | N/A | N/A |
|  |  | s436 | KPGPPLSPEIRSPAGsPELR | 775.6862 | 2 | 20 | 1.352 | 1.125 | 0.896 | 0.789 | 0.6815 | Down | N/A | N/A |
| Claudin-12 | CLDN12 | s231 | SRLsAIEIDIPVVSHTT | 959.4877 | 2 | 14 | 1.475 | 1.695 | 0.988 | 1.357 | 0.735 | Down | N/A | N/A |
| Coiled-coil and C2 domain-containing protein 1A | CC2D1A | s455 | KQNsPVAPTAQPK | 723.361 | 2 | 8 | 1.292 | 0.973 | 0.953 | 0.645 | 0.7 | Down | R | [R] General function prediction only |
| Cyclin-dependent kinase 1 | CDK1 | t161 | VYtHEVVTLWYR | 823.3923 | 2 | 16 | 1.313 | 1.23 | 0.997 | 0.931 | 0.758 | Down | R | [R] General function prediction only |
| Cyclin-dependent kinase 12 | CDK12 | t1244 | RtPTMPQEEAAACPPHILPPEK | 850.7342 | 3 | 11 | 1.068 | 1 | 0.756 | 0.708 | 0.708 | Down | D | [D] Cell cycle control, cell division, chromosome partitioning |
| Cysteine/serine-rich nuclear protein 1 | CSRNP1 | s45 | AWDsEEEGPWDQMPLPDRDFCGPR | 990.7335 | 3 | 4 | 1.046 | 1.292 | 0.839 | 0.895 | 0.7475 | Down | R | [R] General function prediction only |
| Cytoplasmic tRNA 2-thiolation protein 2 | CTU2 | s419 | LSQMQsPIPLTETR | 840.905 | 2 | 10 | 2.222 | 2.37 | 1.335 | 1.366 | 0.5885 | Down | S | [S] Function unknown |
| Death-inducer obliterator 1 | DIDO1 | s805 | QEAIPDLEDsPPVSDSEEQQESAR | 939.3776 | 3 | 8 | 1.069 | 0.952 | 0.726 | 0.697 | 0.706 | Down | R | [R] General function prediction only |
| Disintegrin and metalloproteinase domain-containing protein 17 | ADAM17 | s791 | sFEDLTDHPVTR | 748.8245 | 2 | 8 | 1.203 | 1.288 | 0.758 | 0.844 | 0.643 | Down | W | [W] Extracellular structures |
| Disintegrin and metalloproteinase domain-containing protein 19 | ADAM19 | s802 | GGsPPAPLPAHLSR | 718.8559 | 2 | 4 | 1.252 | 1.234 | 0.786 | 0.803 | 0.6395 | Down | O | [O] Posttranslational modification, protein turnover, chaperones |
| DNA repair protein complementing XP-C cells | XPC | s122 | GATMNEDsNEEEEESENDWEEVEELSEPVLGDVR | 1352.519 | 3 | 8 | 1.24 | 0.861 | 0.987 | 0.604 | 0.749 | Down | L | [L] Replication, recombination and repair |
|  |  | s129 | GATMNEDSNEEEEEsENDWEEVEELSEPVLGDVR | 1352.519 | 3 | 8 | 1.24 | 0.861 | 0.987 | 0.604 | 0.749 | Down | L | [L] Replication, recombination and repair |
| DNA replication licensing factor MCM4 | MCM4 | s131 | GLQVDLQsDGAAAEDIVASEQSLGQK | 903.7587 | 3 | 12 | 1.159 | 1.133 | 0.799 | 0.831 | 0.7125 | Down | L | [L] Replication, recombination and repair |
| DnaJ homolog subfamily C member 9 | DNAJC9 | s109 | KIsLEDIQAFEK | 750.8709 | 2 | 8 | 1.387 | 1.537 | 0.875 | 1.085 | 0.6685 | Down | O | [O] Posttranslational modification, protein turnover, chaperones |
| E3 ubiquitin-protein ligase MYCBP2 | MYCBP2 | s2353 | IPGSPAVTAASSNTDMTYGGLAsPK | 825.0487 | 3 | 4 | 1.269 | 1.076 | 0.882 | 0.851 | 0.7435 | Down | T | [T] Signal transduction mechanisms |
| E3 ubiquitin-protein ligase UBR5 | UBR5 | s327 | WLDGAsFDNER | 695.2772 | 2 | 4 | 1.078 | 1.544 | 0.587 | 0.864 | 0.552 | Down | O | [O] Posttranslational modification, protein turnover, chaperones |
| Enhancer of mRNA-decapping protein 4 | EDC4 | s723 | GPGQVPTATSALSLELQEVEPLGLPQAsPSR | 1070.541 | 2 | 20 | 1.25 | 1.346 | 0.973 | 0.972 | 0.7495 | Down | R | [R] General function prediction only |
| Epidermal growth factor receptor | EGFR | s1166 | GSHQIsLDNPDYQQDFFPK | 1158.502 | 2 | 12 | 1.262 | 1.628 | 0.529 | 0.761 | 0.443 | Down | T | [T] Signal transduction mechanisms |
| ETS domain-containing transcription factor ERF | ERF | s489 | RWsEDCR | 544.7026 | 2 | 4 | 1.273 | 1.083 | 0.966 | 0.759 | 0.729 | Down | K | [K] Transcription |
| Exocyst complex component 2 | EXOC2 | s426 | GNPGLHSPMLDLDNDTRPSVLGHLSQTAsLKR | 877.4357 | 4 | 8 | 1.076 | 1.186 | 0.797 | 0.865 | 0.735 | Down | U | [U] Intracellular trafficking, secretion, and vesicular transport |
| F-actin-uncapping protein LRRC16A | CARMIL1 | t916 | LEDLDTCMMtPK | 767.3054 | 2 | 13 | 1.142 | 1.327 | 0.945 | 0.937 | 0.767 | Down | N | [N] Cell motility |
|  |  | s1101 | GAVRsPPVDCPR | 695.8185 | 2 | 4 | 1.219 | 1.097 | 0.861 | 0.899 | 0.763 | Down | N | [N] Cell motility |
| Focal adhesion kinase 1 | PTK2 | s910 | LQPQEIsPPPTANLDR | 928.4511 | 2 | 8 | 1.946 | 1.921 | 1.403 | 1.469 | 0.743 | Down | T | [T] Signal transduction mechanisms |
| Forkhead box protein C1 | FOXC1 | s320 | GsPQSAAAELSSGLLASAAASSR | 1085.013 | 2 | 8 | 2.398 | 2.562 | 0.658 | 0.67 | 0.2675 | Down | K | [K] Transcription |
| Forkhead box protein K1 | FOXK1 | s416 | sAPASPTHPGLMSPR | 793.3633 | 2 | 19 | 0.978 | 2.003 | 0.622 | 1.517 | 0.6975 | Down | K | [K] Transcription |
| Formin-binding protein 4 | FNBP4 | s116 | ATGGLCLLGAYADsDDDDNDVSEK | 1291.018 | 2 | 8 | 1.14 | 1.145 | 0.798 | 0.791 | 0.696 | Down | N/A | N/A |
| Fos-related antigen 2 | FOSL2 | s120 | RRDEQLsPEEEEK | 862.878 | 2 | 12 | 0.549 | 0.514 | 0.412 | 0.322 | 0.6895 | Down | K | [K] Transcription |
| G protein-regulated inducer of neurite outgrowth 1 | GPRIN1 | t877 | SVATGPMtPQAAAPPAFPEVR | 1088.019 | 2 | 4 | 1.219 | 1.277 | 0.847 | 0.736 | 0.6355 | Down | N/A | N/A |
| General transcription factor 3C polypeptide 2 | GTF3C2 | s901 | RPGFsPTSHR | 611.2799 | 2 | 8 | 1.067 | 0.969 | 0.862 | 0.665 | 0.7475 | Down | N/A | N/A |
| Golgin subfamily A member 4 | GOLGA4 | s71 | VPsVESLFR | 557.2706 | 2 | 8 | 0.947 | 1.058 | 0.525 | 0.613 | 0.567 | Down | N/A | N/A |
| Histone deacetylase 1 | HDAC1 | s421 | IACEEEFsDSEEEGEGGRK | 773.2783 | 2 | 38 | 1.586 | 1.129 | 1.166 | 0.756 | 0.702 | Down | B | [B] Chromatin structure and dynamics |
| Histone-lysine N-methyltransferase 2D | KMT2D | s2274 | ASEPLLsPPPFGESR | 832.39 | 2 | 8 | 0.957 | 0.773 | 0.669 | 0.625 | 0.753 | Down | R | [R] General function prediction only |
| Host cell factor 1 | HCFC1 | s411 | YDIPATAATATsPTPNPVPSVPANPPK | 1377.673 | 2 | 12 | 1.406 | 1.082 | 0.785 | 0.637 | 0.574 | Down | DK | [D] Cell cycle control, cell division, chromosome [K] Transcription partitioning |
| Insulin-like growth factor 2 mRNA-binding protein 2 | IGF2BP2 | s164 | ISYIPDEEVSSPsPPQR | 990.9512 | 2 | 15 | 1.726 | 1.64 | 1.298 | 1.262 | 0.7605 | Down | AR | [A] RNA processing and modification [R] General function prediction only |
| Interferon-inducible double-stranded RNA-dependent protein kinase activator A | PRKRA | s18 | AEAPPLEREDsGTFSLGK | 992.4566 | 2 | 15 | 1.802 | 1.514 | 1.237 | 1.228 | 0.7485 | Down | UK | [U] Intracellular trafficking, secretion, and vesicular [K] Transcription transport |
| Intracellular hyaluronan-binding protein 4 | HABP4 | s7 | GALGsPVAAAGAAMQESFGCVVANR | 1236.064 | 2 | 7 | 1.276 | 1.381 | 0.659 | 0.762 | 0.534 | Down | R | [R] General function prediction only |
| Junctional protein associated with coronary artery disease | JCAD | s255 | VLSPESLSCTEIPIPLNERHsPK | 895.1102 | 3 | 4 | 1.167 | 1.338 | 0.803 | 0.77 | 0.632 | Down | N/A | N/A |
| Kinesin-like protein KIF23 | KIF23 | s912 | RRSsTVAPAQPDGAESEWTDVETR | 935.7341 | 3 | 13 | 1.449 | 1.65 | 1.03 | 0.978 | 0.652 | Down | Z | [Z] Cytoskeleton |
| La-related protein 4B | LARP4B | s524 | FTSSQTQSPTPPKPPsPSFELGLSSFPPLPGAAGNLK | 1282.971 | 3 | 17 | 1.033 | 1.495 | 0.715 | 1.231 | 0.7575 | Down | T | [T] Signal transduction mechanisms |
| Lipolysis-stimulated lipoprotein receptor | LSR | s467 | GPALTPIRDEEWGGHSPRsPR | 799.0483 | 3 | 27 | 0.552 | 0.473 | 0.346 | 0.342 | 0.6755 | Down | N/A | N/A |
| Mediator of RNA polymerase II transcription subunit 13 | MED13 | s395 | KYsASSGGLCEEATAAK | 905.3899 | 2 | 7 | 1.121 | 1.081 | 0.816 | 0.653 | 0.666 | Down | K | [K] Transcription |
| Mediator of RNA polymerase II transcription subunit 13 | MED13 | s890 | IEVDEGFCsPKPSEIK | 957.9314 | 2 | 6 | 1.223 | 1.038 | 0.855 | 0.853 | 0.761 | Down | K | [K] Transcription |
| Membrane-associated phosphatidylinositol transfer protein 1 | PITPNM1 | s373 | WNsNDFIDAFASPVEAEGTPEPGAEAAK | 1000.769 | 3 | 8 | 1.697 | 1.609 | 0.935 | 1.051 | 0.6025 | Down | IT | [I] Lipid transport and metabolism [T] Signal transduction mechanisms |
| Metal transporter CNNM3 | CNNM3 | s700 | PGVPVEGsPGRNPGV | N/A | 2 | 21 | 0.477 | 0.547 | 0.357 | 0.397 | 0.7365 | Down | S | [S] Function unknown |
| Microtubule-associated protein 1B | MAP1B | s1265 | SPSLSPSPPsPLEK | 751.8605 | 2 | 4 | 1.196 | 1.185 | 0.909 | 0.789 | 0.713 | Down | Z | [Z] Cytoskeleton |
| Microtubule-associated protein tau | MAPT | s717 | TDHGAEIVYKSPVVsGDTSPR | 819.0042 | 3 | 9 | 1.387 | 1.496 | 1.076 | 1.064 | 0.7435 | Down | Z | [Z] Cytoskeleton |
| Microtubule-associated serine/threonine-protein kinase 2 | MAST2 | s1256 | SLsSGESGPGSPTHSHSLSPR | 715.3183 | 3 | 6 | 1.103 | 1.624 | 0.792 | 1.293 | 0.757 | Down | TR | [T] Signal transduction mechanisms [R] General function prediction only |
| Microtubule-associated tumor suppressor 1 | MTUS1 | s1268 | NSGSFPSPSIsPR | 706.8139 | 2 | 8 | 1.264 | 1.182 | 0.878 | 0.834 | 0.7 | Down | N/A |  |
| Mothers against decapentaplegic homolog 2 | SMAD2 | t8 | SSILPFtPPVVK | 703.8702 | 2 | 8 | 0.989 | 1.151 | 0.766 | 0.847 | 0.7545 | Down | TK | [T] Signal transduction mechanisms [K] Transcription |
| Multidrug resistance-associated protein 1 | ABCC1 | s930 | HHNsTAELQK | 622.7746 | 2 | 7 | 1.977 | 0.63 | 1.408 | 0.516 | 0.7655 | Down | Q | [Q] Secondary metabolites biosynthesis, transport and catabolism |
| Myosin-10 | MYH10 | s1956 | QLHLEGASLELsDDDTESK | 722.9861 | 3 | 13 | 1.893 | 1.74 | 1.244 | 1.102 | 0.6455 | Down | Z | [Z] Cytoskeleton |
| N-acetyl-D-glucosamine kinase | NAGK | s76 | SLGLSLsGGDQEDAGR | 821.3594 | 2 | 7 | 0.725 | 0.607 | 0.48 | 0.426 | 0.6805 | Down | G | [G] Carbohydrate transport and metabolism |
| NEDD4-binding protein 1 | N4BP1 | s288 | RFsDSEER | 553.2191 | 2 | 11 | 1.534 | 1.864 | 0.823 | 0.841 | 0.494 | Down | S | [S] Function unknown |
| Negative elongation factor B | NELFB | s557 | KPsPAQAAETPALELPLPSVPAPAPL | 881.4662 | 2 | 16 | 1.703 | 1.168 | 1.096 | 0.849 | 0.685 | Down | N/A | N/A |
| Neurabin-2 | PPP1R9B | s100 | ASsLNENVDHSALLK | 839.3958 | 2 | 5 | 1.322 | 1.579 | 0.948 | 1.183 | 0.733 | Down | T | [T] Signal transduction mechanisms |
| Neuroblast differentiation-associated protein AHNAK | AHNAK | t490 | VKtPEMIIQKPK | 746.4039 | 2 | 17 | 0.292 | 0.306 | 0.2 | 0.241 | 0.736 | Down | N/A | N/A |
|  |  | s2397 | ISMPDLDLHLKsPK | 837.4202 | 2 | 23 | 0.801 | 0.466 | 0.468 | 0.275 | 0.5875 | Down | N/A | N/A |
|  |  | s5099 | MYFPDVEFDIKsPK | 898.4043 | 2 | 10 | 0.384 | 0.681 | 0.127 | 0.242 | 0.343 | Down | N/A | N/A |
| Neurosecretory protein VGF | VGF | s420 | QNALLFAEEEDGEAGAEDKRsQEETPGHR | 824.114 | 3 | 8 | 1.377 | 1.159 | 0.795 | 0.75 | 0.6115 | Down | N/A | N/A |
| NGFI-A-binding protein 2 | NAB2 | t434 | LtPPPADLPLALPAHGLWSR | 1101.577 | 3 | 12 | 2.083 | 1.882 | 0.945 | 0.985 | 0.489 | Down | K | [K] Transcription |
| Nuclear mitotic apparatus protein 1 | NUMA1 | s169 | APVPSTCSSTFPEELsPPSHQAK | 845.3805 | 2 | 16 | 1.038 | 0.788 | 0.685 | 0.589 | 0.7035 | Down | N/A | N/A |
| Nuclear pore complex protein Nup153 | NUP153 | s338 | RIPSIVSSPLNsPLDR | N/A | 2 | 11 | 2.821 | 2.823 | 0.259 | 0.245 | 0.0885 | Down | YU | [Y] Nuclear structure [U] Intracellular trafficking, secretion, and vesicular transport |
| Nuclear pore complex protein Nup88 | NUP88 | s50 | NQSPTEAEKPASSSLPSsPPPQLLTR | 933.7902 | 3 | 4 | 1.356 | 0.965 | 0.98 | 0.78 | 0.7655 | Down | YU | [Y] Nuclear structure [U] Intracellular trafficking, secretion, and vesicular transport |
| Nuclear receptor corepressor 2 | NCOR2 | s1539 | QsPLTYEDHGAPFAGHLPR | 725.0003 | 3 | 8 | 1.317 | 1.095 | 1.003 | 0.786 | 0.739 | Down | K | [K] Transcription |
| Paired amphipathic helix protein Sin3a | SIN3A | s832 | GDLsDVEEEEEEEMDVDEATGAVK | 1353.031 | 2 | 19 | 1.39 | 1.142 | 1.03 | 0.897 | 0.763 | Down | B | [B] Chromatin structure and dynamics |
| Palladin | PALLD | s641 | SLPTPAVLLsPTKEPPPLLAK | 1125.131 | 2 | 9 | 1.472 | 1.468 | 0.535 | 0.608 | 0.389 | Down | Z | [Z] Cytoskeleton |
|  |  | s708 | SAPPSPPFPPPPAFPELAACTPPAsPEPMSALASR | 1260.549 | 3 | 4 | 1.595 | 1.283 | 0.692 | 0.518 | 0.4185 | Down | Z | [Z] Cytoskeleton |
|  |  | s1121 | SRSRDsGDENEPIQER | N/A | 3 | 8 | 1.891 | 1.444 | 0.971 | 0.642 | 0.479 | Down | Z | [Z] Cytoskeleton |
| Paralemmin-3 | PALM3 | s303 | VPEVVQTSsPR | 639.8081 | 2 | 8 | 1.948 | 1.571 | 0.798 | 0.735 | 0.4385 | Down | N/A | N/A |
| PC4 and SFRS1-interacting protein | PSIP1 | s106 | QSNASsDVEVEEKETSVSK | 779.832 | 2 | 17 | 1.361 | 1.255 | 0.98 | 0.916 | 0.726 | Down | K | [K] Transcription |
| Pericentriolar material 1 protein | PCM1 | s1776 | NVRSDISDQEEDEEsEGCPVSINLSK | 1059.407 | 3 | 8 | 0.906 | 0.973 | 0.602 | 0.756 | 0.7205 | Down | N/A | N/A |
| Perilipin-3 | PLIN3 | s130 | VsGAQEMVSSAK | 637.2783 | 2 | 7 | 1.333 | 1.279 | 0.752 | 0.718 | 0.5625 | Down | N/A | N/A |
| PHD and RING finger domain-containing protein 1 | PHRF1 | s1229 | LPALGEAHVsPEVATADK | 942.9588 | 2 | 16 | 1.053 | 0.843 | 0.67 | 0.577 | 0.6605 | Down | R | [R] General function prediction only |
| PH-interacting protein | PHIP | s1281 | KVLsDSEDEEKDADVPGTSTR | 813.3384 | 3 | 15 | 1.168 | 0.831 | 0.778 | 0.6 | 0.6935 | Down | R | [R] General function prediction only |
|  |  | s1283 | KVLSDsEDEEKDADVPGTSTR | 813.3384 | 3 | 15 | 1.168 | 0.831 | 0.778 | 0.6 | 0.6935 | Down | R | [R] General function prediction only |
| Phosphatidylinositol 4-kinase alpha | PI4KA | s265 | KTSSVSSISQVsPER | 836.4011 | 2 | 7 | 1.437 | 1.368 | 1.141 | 0.998 | 0.762 | Down | T | [T] Signal transduction mechanisms |
| Plasminogen activator inhibitor 1 RNA-binding protein | SERBP1 | s330 | sEEAHAEDSVMDHHFR | N/A | 3 | 33 | 1.776 | 1.796 | 1.239 | 1.486 | 0.763 | Down | R | [R] General function prediction only |
| Plastin-2 | LCP1 | s5 | GsVSDEEMMELR | 731.7831 | 2 | 4 | 1.473 | 1.322 | 0.659 | 0.629 | 0.4615 | Down | Z | [Z] Cytoskeleton |
| Plectin | PLEC | s1732 | sLQEEHVAVAQLR | 780.3825 | 2 | 5 | 1.419 | 1.344 | 0.94 | 1.073 | 0.7305 | Down | J | [J] Translation, ribosomal structure and biogenesis |
| Pre-B-cell leukemia transcription factor 2 | PBX2 | s330 | TAVSVTQGGHsR | 640.2932 | 2 | 8 | 1.294 | 1.025 | 0.946 | 0.824 | 0.768 | Down | K | [K] Transcription |
| Protein EFR3 homolog A | EFR3A | s694 | LSVPYVPQVTDEDRLsR | 1027.501 | 2 | 15 | 1.17 | 1.302 | 0.94 | 0.938 | 0.7625 | Down | R | [R] General function prediction only |
| Protein HEXIM2 | HEXIM2 | s53 | MESHsEDEDLAGAVGGLGWNSR | 826.3207 | 3 | 8 | 1.453 | 2.055 | 0.672 | 0.911 | 0.4525 | Down | N/A | N/A |
| Protein ITPRID2 | ITPRID2 | s111 | NGGsFEDDLSLGAEANHLHESDAQIENCNNILAK | 941.4139 | 4 | 7 | 1.346 | 1.164 | 0.822 | 0.748 | 0.626 | Down | N/A | N/A |
|  |  | s593 | KSGsQDFPQCNTIENTGTK | 1096.47 | 2 | 8 | 1.245 | 1.327 | 0.786 | 0.803 | 0.6185 | Down | N/A | N/A |
|  |  | s737 | sQSLPTTLLSPVR | 739.8844 | 2 | 15 | 1.618 | 2.685 | 0.797 | 1.217 | 0.4725 | Down | N/A | N/A |
| Protein IWS1 homolog | IWS1 | s363 | FHSsDSEEEEHKK | N/A | 2 | 27 | 1.404 | 0.942 | 1.096 | 0.681 | 0.7515 | Down | S | [S] Function unknown |
| Protein kinase C and casein kinase substrate in neurons protein 3 | PACSIN3 | s319 | sPDEVTLTSIVPTR | 797.8898 | 2 | 8 | 1.51 | 1.091 | 0.79 | 0.693 | 0.579 | Down | TUZ | [T] Signal transduction mechanisms [U] Intracellular trafficking, secretion, and vesicular transport [Z] Cytoskeleton |
| Protein NDRG1 | NDRG1 | s2 | sREMQDVDLAEVKPLVEK | 736.6921 | 2 | 9 | 1.323 | 1.754 | 0.807 | 0.924 | 0.5685 | Down | S | [S] Function unknown |
| Protein O-GlcNAcase | OGA | t709 | LLPIDGANDLFFQPPPLtPTSK | 1231.124 | 2 | 5 | 1.391 | 1.01 | 0.971 | 0.711 | 0.7015 | Down | O | [O] Posttranslational modification, protein turnover, chaperones |
| Protein phosphatase Slingshot homolog 3 | SSH3 | s37 | RQsFAVLR | 528.7711 | 2 | 4 | 0.849 | 1.588 | 0.584 | 1.044 | 0.673 | Down | V | [V] Defense mechanisms |
| Protein SCAF11 | SCAF11 | s405 | SSSNDsVDEETAESDTSPVLEK | 1203.489 | 2 | 7 | 0.806 | 0.394 | 0.623 | 0.251 | 0.7045 | Down | R | [R] General function prediction only |
| Protein scribble homolog | SCRIB | s853 | LPLLPPEsPGPLR | 733.3944 | 2 | 7 | 1.23 | 1.217 | 0.304 | 0.292 | 0.243 | Down | R | [R] General function prediction only |
|  |  | s1448 | QSPAsPPPLGGGAPVR | 784.3851 | 2 | 8 | 1.96 | 1.752 | 0.663 | 0.597 | 0.3395 | Down | R | [R] General function prediction only |
| Protein Shroom2 | SHROOM2 | s456 | HPPLYSDHSPLCADSLGQEPGAASFQNDsPPQVR | 939.4203 | 4 | 4 | 1.189 | 1.132 | 0.923 | 0.834 | 0.757 | Down | N/A | N/A |
| Protein spire homolog 1 | SPIRE1 | t509 | FLPISStPQPER | 726.3501 | 2 | 7 | 1.15 | 1.127 | 0.687 | 0.727 | 0.6205 | Down | N/A | N/A |
|  |  | s678 | SKsMDKSDEELQFPK | 616.941 | 3 | 8 | 1.1 | 1.549 | 0.889 | 1.127 | 0.768 | Down | N/A | N/A |
| Protein transport protein Sec16A | SEC16A | s1069 | AQQELVPPQQQAsPPQLPK | 1082.544 | 2 | 14 | 0.738 | 0.572 | 0.438 | 0.391 | 0.6385 | Down | K | [K] Transcription |
| Protein YIPF2 | YIPF2 | s3 | AsADELTFHEFEEATNLLADTPDAATTSR | 1082.477 | 3 | 8 | 1.143 | 1.228 | 0.726 | 0.696 | 0.601 | Down | S | [S] Function unknown |
| Protocadherin Fat 1 | FAT1 | s4272 | NNLDRNsFEGSAIPEHPEFSTFNPESVHGHR | 901.1525 | 4 | 9 | 0.406 | 0.387 | 0.202 | 0.195 | 0.4995 | Down | T | [T] Signal transduction mechanisms |
| Putative ATP-dependent RNA helicase DDX11-like protein 8 | DDX11L8 | s204 | LEQLESGEEELVLAEYEsDEEKK | 926.0827 | 3 | 10 | 1.376 | 1.385 | 0.983 | 1.089 | 0.7505 | Down | L | [L] Replication, recombination and repair |
| Putative RNA-binding protein 15B | RBM15B | s609 | TTHsPYEER | 600.2401 | 2 | 8 | 0.638 | 0.401 | 0.423 | 0.262 | 0.658 | Down | R | [R] General function prediction only |
| R3H domain-containing protein 1 | R3HDM1 | s142 | DSsQEYTDSTGIDLHEFLVNTLK | 898.0724 | 3 | 6 | 1.308 | 1.34 | 0.951 | 0.962 | 0.7225 | Down | A | [A] RNA processing and modification |
| R3H domain-containing protein 2 | R3HDM2 | s855 | SAsTDLGTADVVLGR | 771.364 | 2 | 5 | 1.371 | 1.919 | 0.527 | 0.845 | 0.4125 | Down | A | [A] RNA processing and modification |
| Radiation-inducible immediate-early gene IEX-1 | IER3 | s31 | RGsGPEIFTFDPLPEPAAAPAGR | 811.7248 | 3 | 4 | 1.369 | 1.173 | 0.761 | 0.777 | 0.6095 | Down | N/A | N/A |
| Ras-related protein Rab-6A | RAB6A | s184 | VAAALPGMESTQDRsREDMIDIK | 871.7398 | 3 | 6 | 1.329 | 1.302 | 0.793 | 0.943 | 0.66 | Down | U | [U] Intracellular trafficking, secretion, and vesicular transport |
| Ras-responsive element-binding protein 1 | RREB1 | s1320 | QVAGDAPVEQATAETAsPVHR | 738.6777 | 2 | 14 | 1.026 | 0.869 | 0.795 | 0.645 | 0.758 | Down | R | [R] General function prediction only |
| Receptor-binding cancer antigen expressed on SiSo cells | EBAG9 | s36 | KLsGDQITLPTTVDYSSVPK | 1115.056 | 2 | 16 | 1.196 | 1.04 | 0.889 | 0.739 | 0.7265 | Down | N/A | N/A |
| RelA-associated inhibitor | PPP1R13L | s113 | SESAPTLHPYSPLsPK | 895.9217 | 2 | 12 | 1.776 | 1.933 | 1.396 | 1.418 | 0.76 | Down | D | [D] Cell cycle control, cell division, chromosome partitioning |
| Rho GTPase-activating protein 39 | ARHGAP39 | s604 | AFsEDEALAQQENR | 844.3516 | 2 | 6 | 1.261 | 1.596 | 0.933 | 1.07 | 0.7055 | Down | T | [T] Signal transduction mechanisms |
| Rho guanine nucleotide exchange factor 16 | ARHGEF16 | s107 | HQsFGAAVLSR | 626.7953 | 2 | 8 | 0.806 | 2.279 | 0.457 | 1.486 | 0.61 | Down | T | [T] Signal transduction mechanisms |
| Rho guanine nucleotide exchange factor 2 | ARHGEF2 | s174 | ILSQsTDSLNMR | 722.8287 | 2 | 6 | 1.104 | 1.702 | 0.774 | 1.278 | 0.726 | Down | T | [T] Signal transduction mechanisms |
| Ribonuclease inhibitor | RNH1 | s2 | sLDIQSLDIQCEELSDAR | 1107.485 | 2 | 7 | 1.418 | 1.17 | 0.8 | 0.634 | 0.553 | Down | S | [S] Function unknown |
| Ribosomal protein S6 kinase alpha-3 | RPS6KA3 | s227 | AYsFCGTVEYMAPEVVNR | 1086.96 | 2 | 38 | 1.174 | 1.256 | 0.932 | 0.907 | 0.758 | Down | T | [T] Signal transduction mechanisms |
| Ribosome biogenesis protein WDR12 | WDR12 | t221 | IWSTVPtDEEDEMEESTNRPR | 867.6985 | 3 | 6 | 1.344 | 1.245 | 0.962 | 0.908 | 0.723 | Down | Z | [Z] Cytoskeleton |
| RNA-binding protein 12 | RBM12 | s420 | sRSRSPHEAGFCVYLK | 711.9508 | 3 | 12 | 1.34 | 1.306 | 1.088 | 0.914 | 0.756 | Down | R | [R] General function prediction only |
| SAFB-like transcription modulator | SLTM | s289 | DGQDAIAQsPEKESK | 841.8671 | 2 | 9 | 1.115 | 0.851 | 0.783 | 0.507 | 0.6495 | Down | K | [K] Transcription |
|  |  | s553 | sPGHMVILDQTK | 703.3309 | 2 | 73 | 1.097 | 0.893 | 0.77 | 0.709 | 0.7475 | Down | K | [K] Transcription |
| SAP30-binding protein | SAP30BP | s18 | NVLSSLAVYAEDsEPESDGEAGIEAVGSAAEEK | 1161.825 | 3 | 14 | 1.496 | 1.26 | 1.007 | 0.877 | 0.6845 | Down | K | [K] Transcription |
| Serine/arginine repetitive matrix protein 2 | SRRM2 | t1003 | AQtPPGPSLSGSKSPCPQEK | 1106.971 | 3 | 37 | 1.02 | 0.797 | 0.793 | 0.585 | 0.755 | Down | A | [A] RNA processing and modification |
|  |  | s2102 | NHSGsRTPPVALNSSR | 587.2794 | 2 | 26 | 1.503 | 0.778 | 1.113 | 0.615 | 0.765 | Down | A | [A] RNA processing and modification |
|  |  | s2123 | PSMsPTPLDR | 590.757 | 2 | 54 | 1.269 | 1.287 | 0.833 | 0.751 | 0.62 | Down | A | [A] RNA processing and modification |
| Serine/threonine-protein kinase MARK2 | MARK2 | s456 | VPAsPLPGLER | 608.3103 | 2 | 8 | 0.949 | 1.129 | 0.686 | 0.79 | 0.7105 | Down | R | [R] General function prediction only |
| Serine/threonine-protein phosphatase PP1-alpha catalytic subunit | PPP1CA | t320 | YGQFSGLNPGGRPItPPR | 665.3263 | 3 | 7 | 1.42 | 1.64 | 1.117 | 1.217 | 0.764 | Down | TR | [T] Signal transduction mechanisms [R] General function prediction only |
| SH2 domain-containing protein 3C | SH2D3C | s440 | VHAAPAAPSATALPAsPVAR | 967.996 | 2 | 4 | 1.365 | 1.347 | 0.633 | 0.735 | 0.5045 | Down | T | [T] Signal transduction mechanisms |
| Sister chromatid cohesion protein PDS5 homolog A | PDS5A | s1097 | SALCNADsPKDPVLPMK | 961.9412 | 2 | 8 | 1.683 | 1.477 | 1.24 | 0.984 | 0.701 | Down | D | [D] Cell cycle control, cell division, chromosome partitioning |
| SLIT-ROBO Rho GTPase-activating protein 2 | SRGAP2 | s427 | STVsETFMSKPSIAK | 846.8993 | 2 | 11 | 1.547 | 1.384 | 1.042 | 1.054 | 0.717 | Down | Z | [Z] Cytoskeleton |
| Son of sevenless homolog 1 | SOS1 | s401 | RLsESACR | 529.7261 | 2 | 4 | 1.294 | 1.088 | 0.901 | 0.797 | 0.715 | Down | T | [T] Signal transduction mechanisms |
| Splicing factor, arginine/serine-rich 19 | SCAF1 | t989 | AAPPPPALtPDSQTVDSSCK | 1035.965 | 2 | 14 | 1.973 | 1.006 | 0.951 | 0.555 | 0.5165 | Down | R | [R] General function prediction only |
| Sprouty-related, EVH1 domain-containing protein 1 | SPRED1 | s238 | HVsFQDEDEIVR | 777.3352 | 2 | 4 | 1.011 | 1.477 | 0.683 | 0.9 | 0.642 | Down | T | [T] Signal transduction mechanisms |
| SR-related and CTD-associated factor 4 | SCAF4 | s154 | IEIIQPLLDMAAGTSNAAPVAENVTNNEGsPPPPVK | 1245.616 | 3 | 18 | 0.935 | 0.766 | 0.7 | 0.603 | 0.7675 | Down | AK | [A] RNA processing and modification [K] Transcription |
| TCF3 fusion partner | TFPT | s249 | LLPYPTLAsPASD | 752.8222 | 2 | 8 | 0.807 | 0.75 | 0.572 | 0.593 | 0.75 | Down | N/A | N/A |
| Tetratricopeptide repeat protein 7A | TTC7A | s697 | LEEAMSELTMPSsVLK | 922.9247 | 2 | 5 | 1.205 | 1.133 | 0.892 | 0.738 | 0.6955 | Down | T | [T] Signal transduction mechanisms |
| Tight junction protein ZO-1 | TJP1 | s297 | DDISEIQSLAsDHSGR | 905.3862 | 2 | 15 | 1.412 | 0.436 | 0.978 | 0.287 | 0.676 | Down | T | [T] Signal transduction mechanisms |
| TOM1-like protein 1 | TOM1L1 | s323 | EATNTTSEPSAPSQDLLDLSPsPR | 865.0604 | 3 | 16 | 0.891 | 0.998 | 0.55 | 0.749 | 0.6835 | Down | U | [U] Intracellular trafficking, secretion, and vesicular transport |
| TP53-binding protein 1 | TP53BP1 | s398 | QDKPMDTSVLsEEGGEPFQK | 1151.501 | 3 | 10 | 1.195 | 1.076 | 0.853 | 0.827 | 0.7415 | Down | L | [L] Replication, recombination and repair |
|  |  | s727 | ECSEAMEVETSVISIDsPQK | 1159.992 | 2 | 10 | 1.283 | 1.178 | 0.975 | 0.915 | 0.7685 | Down | L | [L] Replication, recombination and repair |
| TRAF2 and NCK-interacting protein kinase | TNIK | t581 | tPPMLRPVDPQIPHLVAVK | 884.2139 | 3 | 7 | 1.219 | 1.208 | 0.8 | 0.849 | 0.6795 | Down | T | [T] Signal transduction mechanisms |
| Transcription factor AP-1 | JUN | s63 | NSDLLTsPDVGLLK | 776.3869 | 2 | 8 | 1.283 | 1.404 | 0.909 | 0.85 | 0.6565 | Down | K | [K] Transcription |
| Transcriptional repressor p66-alpha | GATAD2A | s100 | RPPsPDVIVLSDNEQPSSPR | 810.6762 | 3 | 40 | 1.281 | 1.029 | 0.892 | 0.827 | 0.7505 | Down | S | [S] Function unknown |
| Transducin-like enhancer protein 3 | TLE3 | s240 | YDsDGDKSDDLVVDVSNEDPATPR | 897.0431 | 3 | 8 | 1.757 | 1.699 | 0.935 | 0.791 | 0.499 | Down | B | [B] Chromatin structure and dynamics |
| Trinucleotide repeat-containing gene 18 protein | TNRC18 | s1136 | LALSPEDKPIRLsPSK | 610.9957 | 2 | 21 | 0.738 | 0.882 | 0.401 | 0.556 | 0.587 | Down | K | [K] Transcription |
|  |  | s1857 | ALsPGLEESGLGLLAR | 831.9268 | 2 | 8 | 0.683 | 0.753 | 0.508 | 0.516 | 0.714 | Down | K | [K] Transcription |
| TRMT1-like protein | TRMT1L | s66 | DSAGVPAPAPDSALDSAPTPASAPAPAPALAQAPALsPSLASAPEEAK | 1121.049 | 4 | 7 | 1.519 | 1.405 | 1.056 | 1.155 | 0.759 | Down | J | [J] Translation, ribosomal structure and biogenesis |
| Tropomodulin-3 GN=TMOD3 | TMOD3 | s25 | YKDLDEDELLGNLsETELK | N/A | 3 | 17 | 1.397 | 1.223 | 1.009 | 0.995 | 0.7675 | Down | Z | [Z] Cytoskeleton |
| Tumor necrosis factor receptor superfamily member 16 | NGFR | s313 | LHSDSGISVDSQsLHDQQPHTQTASGQALK | 813.8784 | 4 | 8 | 1.435 | 1.126 | 0.876 | 0.647 | 0.592 | Down | N/A | N/A |
| Tumor protein D52 | TPD52 | s176 | NSPTFKsFEEKVENLK | 988.9719 | 2 | 17 | 1.397 | 1.457 | 0.993 | 1.061 | 0.7195 | Down | R | [R] General function prediction only |
| WD repeat-containing protein 43 | WDR43 | s77 | LQAKEsPQRK | 632.8241 | 2 | 26 | 1.498 | 1.055 | 1.014 | 0.602 | 0.6235 | Down | R | [R] General function prediction only |
| Zinc finger and BTB domain-containing protein 17 | ZBTB17 | s120 | SLAEPATsPGGNAEALATEGGDKR | 793.699 | 3 | 12 | 1.344 | 1.186 | 0.741 | 0.676 | 0.5605 | Down | R | [R] General function prediction only |
| Zinc finger CCCH domain-containing protein 8 | ZC3H8 | s77 | SKDYDVYsDNDICSQESEDNFAK | 937.0321 | 3 | 13 | 1.179 | 1.054 | 0.709 | 0.701 | 0.633 | Down | A | [A] RNA processing and modification |
| Zinc finger protein 316 | ZNF316 | s112 | GGDAKsPVLQEK | 654.8134 | 2 | 5 | 1.413 | 1.197 | 0.947 | 0.733 | 0.6415 | Down | R | [R] General function prediction only |
| Zinc finger protein 7 | ZNF7 | s138 | ISPQDFPQNPGFGDVSDSEVWLDSHLGsPGLK | 1169.203 | 3 | 5 | 2.008 | 1.822 | 0.478 | 0.392 | 0.2275 | Down | R | [R] General function prediction only |
| (E3-independent) E2 ubiquitin-conjugating enzyme | UBE2O | s515 | KKsIPLSIK | 547.3227 | 2 | 4 | 0.722 | 0.95 | 1.034 | 1.355 | 1.428 | Up | O | [O] Posttranslational modification, protein turnover, chaperones |
| 182 kDa tankyrase-1-binding protein | TNKS1BP1 | s601 | YESQEPLAGQEsPLPLATR | 1083.509 | 2 | 8 | 0.534 | 0.604 | 0.7 | 0.801 | 1.3175 | Up | N/A | N/A |
| 26S proteasome regulatory subunit 6A | PSMC3 | s9 | MNLLPNIEsPVTR | 803.3889 | 2 | 8 | 0.963 | 0.933 | 1.36 | 1.574 | 1.5495 | Up | O | [O] Posttranslational modification, protein turnover, chaperones |
| Actin-binding LIM protein 1 | ABLIM1 | s587 | RSsGREEDDEELLR | 590.9267 | 3 | 13 | 1.22 | 1.209 | 1.93 | 1.614 | 1.458 | Up | TZ | [T] Signal transduction mechanisms [Z] Cytoskeleton |
| Actin-binding LIM protein 3 | ABLIM3 | s373 | ASsPGYIDSPTYSR | 790.8351 | 2 | 4 | 0.916 | 0.776 | 1.355 | 1.026 | 1.399 | Up | TZ | [T] Signal transduction mechanisms [Z] Cytoskeleton |
| Activity-dependent neuroprotector homeobox protein | ADNP | s1071 | LSNPQIEWQNSTIDsEDGEQFDNMTDGVAEPMHGSLAGVK | 1107.483 | 4 | 8 | 0.902 | 0.886 | 1.455 | 1.44 | 1.619 | Up | N/A | N/A |
| Afadin | AFDN | s1779 | SQDADsPGSSGAPENLTFK | 994.4177 | 2 | 18 | 0.706 | 0.815 | 1.005 | 1.315 | 1.5185 | Up | Z | [Z] Cytoskeleton |
| Amyloid-beta A4 precursor protein-binding family B member 2 | APBB2 | s123 | NLsPTAVINITSEK | 783.8924 | 2 | 8 | 0.873 | 0.855 | 1.596 | 1.335 | 1.6955 | Up | N/A | N/A |
| Annexin A1 | ANXA1 | s37 | GGPGSAVsPYPTFNPSSDVAALHK | 812.7126 | 3 | 6 | 1.087 | 1.229 | 1.509 | 1.68 | 1.3765 | Up | U | [U] Intracellular trafficking, secretion, and vesicular transport |
| Anoctamin-6 | ANO6 | s909 | MIEAVDNNLRPKsE | 848.3922 | 2 | 4 | 0.931 | 0.806 | 1.148 | 1.184 | 1.351 | Up | S | [S] Function unknown |
| AP-1 complex-associated regulatory protein | AP1AR | t228 | SKtEEDILR | 585.7737 | 2 | 4 | 0.726 | 0.687 | 1.443 | 1.212 | 1.876 | Up | N/A | N/A |
| Arf-GAP with SH3 domain, ANK repeat and PH domain-containing protein 2 | ASAP2 | s701 | LLHEDLDEsDDDMDEK | 999.8797 | 2 | 8 | 0.852 | 0.789 | 1.404 | 1.077 | 1.507 | Up | T | [T] Signal transduction mechanisms |
| Arginine/serine-rich coiled-coil protein 2 | RSRC2 | s32 | KKEQSEVSVsPR | 727.356 | 2 | 31 | 1.056 | 0.809 | 1.561 | 1.064 | 1.3955 | Up | N/A | N/A |
| Aryl hydrocarbon receptor nuclear translocator-like protein 1 | ARNTL | s42 | KGsSTDYQESMDTDKDDPHGR | 816.9918 | 3 | 6 | 1.029 | 0.924 | 1.25 | 1.313 | 1.3185 | Up | K | [K] Transcription |
| Ataxin-2-like protein | ATXN2L | s339 | QGSGREsPSLASR | 706.3199 | 2 | 7 | 0.457 | 0.994 | 0.677 | 1.372 | 1.4305 | Up | A | [A] RNA processing and modification |
|  |  | s391 | GPHHLDNSsPGPGSEAR | 897.8814 | 2 | 6 | 0.589 | 0.818 | 1.083 | 1.577 | 1.882 | Up | A | [A] RNA processing and modification |
| ATP-dependent RNA helicase A | DHX9 | s688 | YQILPLHsQIPR | 772.9029 | 2 | 7 | 0.933 | 0.697 | 1.322 | 1.112 | 1.505 | Up | K | [K] Transcription |
| AT-rich interactive domain-containing protein 1A | ARID1A | s382 | TPQPSsPMDQMGK | 742.3015 | 2 | 8 | 0.561 | 0.489 | 0.749 | 0.645 | 1.327 | Up | B | [B] Chromatin structure and dynamics |
| AT-rich interactive domain-containing protein 4B | ARID4B | s778 | VHADLVISKPVSKsPER | 648.0101 | 3 | 14 | 0.925 | 0.652 | 1.341 | 0.876 | 1.3965 | Up | BK | [B] Chromatin structure and dynamics [K] Transcription |
| Autophagy-related protein 9A | ATG9A | s735 | REsDESGESAPDEGGEGAR | 1007.885 | 2 | 7 | 1.413 | 1.166 | 1.902 | 1.517 | 1.3235 | Up | R | [R] General function prediction only |
| B-cell CLL/lymphoma 9-like protein | BCL9L | s915 | RPsDLTISINQMGSPGMGHLK | 773.7044 | 3 | 8 | 0.969 | 1.382 | 1.482 | 1.856 | 1.4365 | Up | N/A | N/A |
| BCL2/adenovirus E1B 19 kDa protein-interacting protein 2 | BNIP2 | s114 | KGsITEYTAAEEK | 753.8398 | 2 | 6 | 0.735 | 0.903 | 1.044 | 1.162 | 1.354 | Up | TZ | [T] Signal transduction mechanisms [Z] Cytoskeleton |
| Bcl-2-associated transcription factor 1 | BCLAF1 | s496 | KETQsPEQVK | 627.2923 | 2 | 10 | 0.548 | 0.427 | 1.217 | 1.118 | 2.4185 | Up | N/A | N/A |
| BET1-like protein | BET1L | s37 | LKsLALDIDRDAEDQNR | 684.6634 | 3 | 4 | 0.613 | 0.689 | 1.356 | 1.404 | 2.1245 | Up | U | [U] Intracellular trafficking, secretion, and vesicular transport |
| Beta/gamma crystallin domain-containing protein 1 | CRYBG1 | s81 | AALDGGVASAASPESKPsPGTK | 1079.477 | 2 | 14 | 0.184 | 0.374 | 0.267 | 0.619 | 1.555 | Up | N/A | N/A |
| BMP-2-inducible protein kinase | BMP2K | s689 | NVDSLSAPHNHPPEDPFGSVPFISHSGsPEK | 873.407 | 4 | 12 | 0.813 | 0.801 | 1.239 | 1.067 | 1.428 | Up | T | [T] Signal transduction mechanisms |
| cAMP-dependent protein kinase catalytic subunit PRKX | PRKX | t203 | TWtLCGTPEYLAPEVIQSK | 1137.032 | 2 | 6 | 0.966 | 1.208 | 1.461 | 1.517 | 1.3855 | Up | T | [T] Signal transduction mechanisms |
| CAP-Gly domain-containing linker protein 1 | CLIP1 | s348 | KIsGTTALQEALK | 720.3789 | 2 | 8 | 0.946 | 0.972 | 1.671 | 1.97 | 1.897 | Up | ZR | [Z] Cytoskeleton [R] General function prediction only |
| Carbohydrate-responsive element-binding protein | MLXIPL | s614 | RLsGDLSSMPGPGTLSVR | 955.4637 | 2 | 4 | 0.829 | 0.826 | 1.093 | 1.317 | 1.4555 | Up | K | [K] Transcription |
| Catenin delta-1 | CTNND1 | s230 | HYEDGYPGGSDNYGsLSR | 1027.4 | 2 | 14 | 0.492 | 0.735 | 0.664 | 0.963 | 1.33 | Up | TW | [T] Signal transduction mechanisms [W] Extracellular structures |
|  |  | s864 | SQSSHsYDDSTLPLIDR | 1000.933 | 2 | 20 | 0.605 | 0.66 | 0.862 | 0.97 | 1.4475 | Up | TW | [T] Signal transduction mechanisms [W] Extracellular structures |
| Cdc42 effector protein 1 | CDC42EP1 | s101 | MAsPPAPSPAPPAISPIIK | 961.4965 | 2 | 7 | 0.949 | 0.964 | 1.398 | 1.47 | 1.499 | Up | N/A | N/A |
|  |  | s350 | AsWESLDEEWR | 744.2956 | 2 | 10 | 0.874 | 1.319 | 1.129 | 1.911 | 1.371 | Up | N/A | N/A |
| Cell division cycle-associated protein 3 | CDCA3 | s68 | HAQDSDPRsPTLGIAR | 600.9511 | 2 | 13 | 0.68 | 0.896 | 0.813 | 1.282 | 1.3125 | Up | N/A | N/A |
| Centrin-2 | CETN2 | s20 | MsPKPELTEEQKQEIR | N/A | 3 | 43 | 0.91 | 0.81 | 1.208 | 1.134 | 1.3635 | Up | ZD | [Z] Cytoskeleton [D] Cell cycle control, cell division, chromosome partitioning |
| Charged multivesicular body protein 7 | CHMP7 | s417 | IsDAELEAELEK | 713.8211 | 2 | 8 | 0.771 | 0.781 | 1.087 | 1.047 | 1.374 | Up | S | [S] Function unknown |
| Chromatin assembly factor 1 subunit A | CHAF1A | t722 | VLQQFAACFLETLPAQEEQtPK | 876.752 | 3 | 14 | 0.986 | 1.006 | 1.394 | 1.313 | 1.3585 | Up | B | [B] Chromatin structure and dynamics |
| Chromobox protein homolog 3 | CBX3 | s93 | RKsLSDSESDDSK | 767.3251 | 2 | 11 | 0.948 | 0.759 | 1.307 | 1.202 | 1.482 | Up | B | [B] Chromatin structure and dynamics |
| Chromosome-associated kinesin KIF4A | KIF4A | t1181 | KtPPAPSPFDLPELK | 858.934 | 2 | 10 | 0.811 | 0.941 | 1.201 | 1.337 | 1.4505 | Up | Z | [Z] Cytoskeleton |
| Clathrin heavy chain 1 | CLTC | s1494 | TSIDAYDNFDNIsLAQR | 1011.944 | 2 | 8 | 1.165 | 0.896 | 1.401 | 1.306 | 1.33 | Up | U | [U] Intracellular trafficking, secretion, and vesicular transport |
| CLIP-associating protein 2 | CLASP2 | s370 | SRsDIDVNAAAGAK | 727.8354 | 2 | 8 | 0.775 | 1.057 | 1.229 | 1.604 | 1.552 | Up | R | [R] General function prediction only |
| Coiled-coil domain-containing protein 97 | CCDC97 | s212 | TPTHQPPKPGsPGRPACPLSNLLLQSYEER | 852.6696 | 4 | 8 | 0.974 | 0.922 | 1.389 | 1.251 | 1.392 | Up | S | [S] Function unknown |
| Constitutive coactivator of PPAR-gamma-like protein 1 | FAM120A | t655 | SPQtPELVEALAFR | 819.4004 | 2 | 8 | 1.145 | 1.145 | 1.462 | 1.615 | 1.344 | Up | N/A | N/A |
| Copper-transporting ATPase 1 | ATP7A | s1466 | ASINsLLSDKR | 642.3214 | 2 | 9 | 0.425 | 0.429 | 0.715 | 0.632 | 1.577 | Up | P | [P] Inorganic ion transport and metabolism |
| CREB/ATF bZIP transcription factor | CREBZF | s50 | AAAGEEETAAAGsPGRK | 826.8674 | 2 | 8 | 1.02 | 0.707 | 1.778 | 1.237 | 1.7455 | Up | N/A | N/A |
| CTD small phosphatase-like protein 2 | CTDSPL2 | s104 | RKsQVNGEAGSYEMTNQHVK | 781.6898 | 3 | 7 | 0.798 | 1.214 | 1.176 | 1.479 | 1.3465 | Up | K | [K] Transcription |
| CTD small phosphatase-like protein | CTDSPL | s268 | EDDVYsMLHR | 672.7681 | 2 | 8 | 0.434 | 0.409 | 0.894 | 0.704 | 1.8885 | Up | K | [K] Transcription |
| Cyclin-dependent kinase 16 | CDK16 | s119 | RLsLPADIR | 560.7974 | 2 | 8 | 0.77 | 1.161 | 1.701 | 2.311 | 2.0995 | Up | R | [R] General function prediction only |
| Cyclin-dependent kinase 18 | CDK18 | s98 | RLsLPMDIR | 590.799 | 2 | 5 | 0.751 | 1.35 | 1.152 | 1.753 | 1.416 | Up | R | [R] General function prediction only |
| Cysteine and histidine-rich domain-containing protein 1 | CHORDC1 | t47 | RRtTDFSDFLSIVGCTK | 694.9941 | 3 | 7 | 1 | 1.034 | 1.369 | 1.345 | 1.3355 | Up | R | [R] General function prediction only |
| Dedicator of cytokinesis protein 5 | DOCK5 | s1834 | NsTELAPPLPVR | 687.3449 | 2 | 8 | 0.948 | 0.992 | 1.722 | 1.714 | 1.7715 | Up | T | [T] Signal transduction mechanisms |
| Deoxynucleotidyltransferase terminal-interacting protein 2 | DNTTIP2 | s381 | WNNNKKsPIK | 654.8266 | 2 | 8 | 0.434 | 0.515 | 1.111 | 1.344 | 2.584 | Up | S | [S] Function unknown |
| Dihydropyrimidinase-related protein 3 | DPYSL3 | t509 | GMYDGPVFDLTTtPK | 861.3782 | 2 | 9 | 0.692 | 0.806 | 1.088 | 1.109 | 1.4735 | Up | F | [F] Nucleotide transport and metabolism |
| DIS3-like exonuclease 2 | DIS3L2 | s139 | VVKPESNDKETEAAYEsDIPEELCGHHLPQQSLK | 990.2108 | 4 | 8 | 0.887 | 0.831 | 1.611 | 1.762 | 1.9675 | Up | J | [J] Translation, ribosomal structure and biogenesis |
| Discoidin, CUB and LCCL domain-containing protein 2 | DCBLD2 | s606 | YSSSEVNHLsPR | 728.3168 | 2 | 4 | 0.963 | 0.708 | 1.276 | 1.125 | 1.458 | Up | O | [O] Posttranslational modification, protein turnover, chaperones |
| Disks large homolog 5 | DLG5 | s1666 | RLsMSEVK | N/A | 2 | 20 | 0.918 | 0.882 | 1.89 | 1.575 | 1.921 | Up | F | [F] Nucleotide transport and metabolism |
| DNA (cytosine-5)-methyltransferase 1 | DNMT1 | s732 | NRIsWVGEAVK | 669.8319 | 2 | 8 | 0.831 | 0.892 | 1.24 | 1.195 | 1.416 | Up | B | [B] Chromatin structure and dynamics |
| DNA mismatch repair protein Msh6 | MSH6 | s830 | IHNVGsPLK | 522.7655 | 2 | 4 | 0.945 | 0.672 | 1.408 | 1.049 | 1.5255 | Up | L | [L] Replication, recombination and repair |
| DNA topoisomerase 2-alpha | TOP2A | s1106 | VPDEEENEEsDNEKETEK | 1115.431 | 2 | 13 | 0.717 | 0.524 | 0.945 | 0.793 | 1.4165 | Up | B | [B] Chromatin structure and dynamics |
| DNA-dependent protein kinase catalytic subunit | PRKDC | s893 | RLsFAVPFR | 586.8024 | 2 | 8 | 0.757 | 0.801 | 1.08 | 1.037 | 1.361 | Up | L | [L] Replication, recombination and repair |
| DNA-directed RNA polymerase II subunit GRINL1A | POLR2M | s179 | VSsQAEDTSSSFDNLFIDR | 1099.468 | 2 | 5 | 1.17 | 0.917 | 1.419 | 1.317 | 1.324 | Up | N/A | N/A |
| Dual specificity mitogen-activated protein kinase kinase 2 | MAP2K2 | s306 | PRPPGRPVsGHGMDSR | 594.9454 | 3 | 11 | 1.047 | 0.954 | 1.458 | 1.53 | 1.498 | Up | T | [T] Signal transduction mechanisms |
| E3 SUMO-protein ligase RanBP2 | RANBP2 | s1993 | ANTsGDFEKDDDAYK | 963.9081 | 2 | 12 | 0.753 | 0.739 | 1.109 | 0.992 | 1.4095 | Up | U | [U] Intracellular trafficking, secretion, and vesicular transport |
| E3 ubiquitin-protein ligase DTX3L | DTX3L | s9 | ASHLRPPsPLLVR | 782.9216 | 2 | 9 | 0.351 | 0.367 | 0.556 | 0.504 | 1.4775 | Up | N/A | N/A |
| E3 ubiquitin-protein ligase HACE1 | HACE1 | s385 | NKRDsTEITSILLK | N/A | 2 | 8 | 1.132 | 1.135 | 1.526 | 1.476 | 1.325 | Up | M | [M] Cell wall/membrane/envelope biogenesis |
| E3 ubiquitin-protein ligase HECTD1 | HECTD1 | s1772 | RQFsALVPAFDPRPGR | N/A | 3 | 17 | 1.295 | 1.471 | 1.867 | 1.863 | 1.354 | Up | R | [R] General function prediction only |
| E3 ubiquitin-protein ligase MGRN1 | MGRN1 | s524 | AASIENVLQDSsPEHCGR | 1025.438 | 2 | 15 | 0.862 | 0.835 | 1.139 | 1.27 | 1.4205 | Up | O | [O] Posttranslational modification, protein turnover, chaperones |
| E3 ubiquitin-protein ligase RAD18 | RAD18 | s99 | NHLLQFALEsPAK | 787.0292 | 2 | 24 | 0.745 | 0.832 | 1.05 | 1.003 | 1.3065 | Up | L | [L] Replication, recombination and repair |
| E3 ubiquitin-protein ligase RNF213 | RNF213 | s2273 | DFATPSLHTSDQsPGK | 884.3829 | 2 | 7 | 0.821 | 0.684 | 1.107 | 0.935 | 1.358 | Up | N/A | N/A |
| E3 ubiquitin-protein ligase Topors | TOPORS | s585 | VYsPYNHR | 558.2371 | 2 | 6 | 0.866 | 0.504 | 1.428 | 0.945 | 1.7625 | Up | K | [K] Transcription |
| E3 ubiquitin-protein ligase ZNRF2 | ZNRF2 | s82 | sLGGAVGSVASGAR | 634.8034 | 2 | 7 | 1.269 | 1.105 | 1.772 | 1.559 | 1.4035 | Up | O | [O] Posttranslational modification, protein turnover, chaperones |
| EH domain-containing protein 2 | EHD2 | s468 | SKYDEIFYNLAPADGKLsGSK | 795.0454 | 3 | 14 | 0.764 | 0.934 | 1.103 | 1.263 | 1.397 | Up | TU | [T] Signal transduction mechanisms [U] Intracellular trafficking, secretion, and vesicular transport |
| Emerin | EMD | s171 | DSAYQSITHYRPVsASR | 1009.46 | 2 | 9 | 1.044 | 1.287 | 1.392 | 2.075 | 1.473 | Up | N/A | N/A |
| Endonuclease/exonuclease/phosphatase family domain-containing protein 1 | EEPD1 | s173 | REHGPFRsVEDLVR | N/A | 3 | 24 | 0.572 | 0.55 | 1.256 | 1.189 | 2.178 | Up | K | [K] Transcription |
| Epidermal growth factor receptor kinase substrate 8 | EPS8 | s476 | RLsTEHSSVSEYHPADGYAFSSNIYTR | 789.353 | 4 | 14 | 0.821 | 0.925 | 1.799 | 1.967 | 2.159 | Up | T | [T] Signal transduction mechanisms |
| Equilibrative nucleoside transporter 1 | SLC29A1 | s254 | LDLIsKGEEPR | 668.8291 | 2 | 8 | 0.868 | 1.081 | 1.522 | 1.574 | 1.6055 | Up | F | [F] Nucleotide transport and metabolism |
| ETS-related transcription factor Elf-1 | ELF1 | s187 | PPRPDsPATTPNISVK | 878.9351 | 2 | 22 | 0.642 | 0.583 | 0.933 | 0.723 | 1.347 | Up | K | [K] Transcription |
| Eukaryotic translation initiation factor 2-alpha kinase 3 | EIF2AK3 | s715 | EHIEIIAPsPQR | 735.361 | 2 | 8 | 0.908 | 0.61 | 1.142 | 0.845 | 1.3225 | Up | J | [J] Translation, ribosomal structure and biogenesis |
| Eukaryotic translation initiation factor 4B | EIF4B | s192 | NRDsDKTDTDWR | 794.823 | 2 | 8 | 1.023 | 1.073 | 1.581 | 1.526 | 1.484 | Up | R | [R] General function prediction only |
| Eukaryotic translation initiation factor 4E-binding protein 1 | EIF4EBP1 | s65 | NsPVTKTPPR | 997.2155 | 2 | 19 | 0.838 | 0.753 | 1.49 | 1.541 | 1.912 | Up | N/A | N/A |
| Exosome complex exonuclease RRP44 | DIS3 | s215 | LACLsEEGNEIESGK | 858.3633 | 2 | 7 | 0.668 | 0.581 | 1.164 | 1.217 | 1.918 | Up | J | [J] Translation, ribosomal structure and biogenesis |
| Forkhead box protein K1 | FOXK1 | s223 | EEAPASPLRPLYPQIsPLK | 1133.55 | 2 | 26 | 0.396 | 0.35 | 1.862 | 1.894 | 5.06 | Up | K | [K] Transcription |
| GAS2-like protein 3 | GAS2L3 | s570 | SALNLNQPVSVSSVsPVK | 953.4877 | 2 | 8 | 0.956 | 0.945 | 1.389 | 1.61 | 1.578 | Up | Z | [Z] Cytoskeleton |
| General transcription factor 3C polypeptide 2 | GTF3C2 | s132 | RPQQPNPPSAPLVPGLLDQsNPLSTPMPK | 1052.869 | 3 | 8 | 0.873 | 1.289 | 1.337 | 1.766 | 1.451 | Up | N/A | N/A |
| Glycogen [starch] synthase, muscle | GYS1 | s653 | HSsPHQSEDEEDPR | 905.3093 | 2 | 8 | 0.988 | 0.872 | 1.831 | 1.373 | 1.714 | Up | G | [G] Carbohydrate transport and metabolism |
|  |  | s657 | HSSPHQsEDEEDPR | 905.3093 | 2 | 8 | 0.988 | 0.872 | 1.831 | 1.373 | 1.714 | Up | G | [G] Carbohydrate transport and metabolism |
|  |  | s710 | RNsVDTATSSSLSTPSEPLSPTSSLGEER | 1024.805 | 3 | 4 | 0.608 | 0.901 | 1.048 | 1.502 | 1.696 | Up | G | [G] Carbohydrate transport and metabolism |
| Glutamate-rich WD repeat-containing protein 1 | GRWD1 | s119 | MHNLHGTKPPPsEGSDEEEEEEDEEDEEER | 1223.784 | 3 | 7 | 0.812 | 0.728 | 1.184 | 1.155 | 1.523 | Up | R | [R] General function prediction only |
|  |  | s122 | MHNLHGTKPPPSEGsDEEEEEEDEEDEEER | 898.0981 | 3 | 11 | 0.76 | 0.728 | 1.109 | 1.082 | 1.4725 | Up | R | [R] General function prediction only |
| Golgi resident protein GCP60 | ACBD3 | s43 | LEVSVDGLTLSPDPEERPGAEGAPLLPPPLPPPsPPGSGR | 1359.019 | 3 | 9 | 0.876 | 0.849 | 1.304 | 1.145 | 1.4185 | Up | U | [U] Intracellular trafficking, secretion, and vesicular transport |
| GON-4-like protein | GON4L | s346 | GVVIPTWNIsPIKK | 816.4497 | 2 | 11 | 1.02 | 1.023 | 1.461 | 1.326 | 1.365 | Up | N/A | N/A |
| G-patch domain and KOW motifs-containing protein | GPKOW | t216 | GLGLGANLTEAQALtPTGPSR | 1052.525 | 2 | 5 | 0.767 | 1.038 | 1.028 | 1.371 | 1.332 | Up | R | [R] General function prediction only |
| GPN-loop GTPase 1 | GPN1 | s314 | DSLsPVLHPSDLILTR | 921.9717 | 2 | 8 | 1.295 | 1.439 | 1.75 | 1.902 | 1.3365 | Up | L | [L] Replication, recombination and repair |
| G-protein-signaling modulator 2 | GPSM2 | s408 | RHsMENMELMKLTPEK | N/A | 3 | 14 | 0.608 | 0.336 | 1.03 | 0.581 | 1.7115 | Up | T | [T] Signal transduction mechanisms |
| GTPase-activating protein and VPS9 domain-containing protein 1 | GAPVD1 | t762 | EVSSRPStPGLSVVSGISATSEDIPNK | 958.7772 | 3 | 20 | 1.008 | 0.922 | 1.472 | 1.457 | 1.5205 | Up | U | [U] Intracellular trafficking, secretion, and vesicular transport |
|  |  | s1019 | FSTLTDDPsPR | 658.2819 | 2 | 7 | 1.019 | 1.026 | 1.515 | 1.652 | 1.5485 | Up | U | [U] Intracellular trafficking, secretion, and vesicular transport |
| H/ACA ribonucleoprotein complex non-core subunit NAF1 | NAF1 | s315 | NDQEPPPEALDFsDDEKEK | 1141.97 | 2 | 15 | 0.684 | 0.705 | 1.351 | 1.242 | 1.869 | Up | S | [S] Function unknown |
| Heat shock protein HSP 90-alpha | HSP90AA1 | s263 | PEIEDVGsDEEEEKKDGDK | N/A | 2 | 95 | 1.234 | 0.962 | 1.645 | 1.339 | 1.363 | Up | O | [O] Posttranslational modification, protein turnover, chaperones |
| Heterogeneous nuclear ribonucleoprotein A1 | HNRNPA1 | s95 | EDsQRPGAHLTVK | 759.359 | 2 | 9 | 0.893 | 0.861 | 1.254 | 1.394 | 1.511 | Up | R | [R] General function prediction only |
| Heterogeneous nuclear ribonucleoproteins C1/C2 | HNRNPC | s260 | MESEGGADDsAEEGDLLDDDDNEDRGDDQLELIKDDEK | 1070.176 | 4 | 36 | 0.736 | 0.629 | 1.293 | 1.125 | 1.772 | Up | R | [R] General function prediction only |
| Histone acetyltransferase KAT7 | KAT7 | s164 | DSGsDLSHRPK | 639.7773 | 2 | 10 | 0.957 | 0.5 | 1.671 | 0.728 | 1.601 | Up | B | [B] Chromatin structure and dynamics |
| Histone H1.3 | H1-3 | s37 | KAsGPPVSELITK | 703.8682 | 2 | 8 | 1.163 | 0.799 | 1.462 | 1.219 | 1.391 | Up | B | [B] Chromatin structure and dynamics |
| Histone H1.4 | H1-4 | s2 | sETAPAAPAAPAPAEKTPVKK | 1053.481 | 2 | 10 | 0.902 | 0.903 | 1.296 | 1.128 | 1.344 | Up | B | [B] Chromatin structure and dynamics |
| Histone H4 | H4C1 | s48 | RIsGLIYEETR | 708.8478 | 2 | 8 | 0.672 | 0.64 | 1.338 | 1.296 | 2.0095 | Up | B | [B] Chromatin structure and dynamics |
| Histone-lysine N-methyltransferase 2D | KMT2D | s4738 | ALsPVIPLIPR | 628.3623 | 2 | 8 | 0.506 | 0.459 | 0.629 | 0.639 | 1.318 | Up | R | [R] General function prediction only |
| Histone-lysine N-methyltransferase SETD2 | SETD2 | s744 | KSEsPFRETEPLVSPHQDK | N/A | 3 | 27 | 0.759 | 0.713 | 1.076 | 0.863 | 1.3135 | Up | U | [U] Intracellular trafficking, secretion, and vesicular transport |
| HIV Tat-specific factor 1 | HTATSF1 | s452 | TEDGGEFEEGASENNAKEsSPEKEAEEGCPEK | 1210.792 | 3 | 5 | 1.013 | 0.765 | 1.584 | 1.024 | 1.4515 | Up | K | [K] Transcription |
|  |  | s481 | RGFEGSCsQKESEEGNPVR | 744.9828 | 3 | 9 | 1.047 | 0.828 | 1.422 | 1.312 | 1.4705 | Up | K | [K] Transcription |
| InaD-like protein | PATJ | t335 | DPAGDISVtPPAPAALPVALPTVASK | 1268.159 | 2 | 12 | 0.589 | 0.611 | 0.872 | 0.834 | 1.4225 | Up | S | [S] Function unknown |
| Inhibitor of nuclear factor kappa-B kinase subunit beta | IKBKB | s672 | GPVSGsPDSMNASR | 721.2925 | 2 | 7 | 0.808 | 0.555 | 1.064 | 0.891 | 1.4635 | Up | T | [T] Signal transduction mechanisms |
| Inosine-5'-monophosphate dehydrogenase 2 | IMPDH2 | s122 | KYEQGFITDPVVLsPK | 950.9764 | 2 | 27 | 1.125 | 1.124 | 1.596 | 1.492 | 1.373 | Up | F | [F] Nucleotide transport and metabolism |
| Insulin receptor substrate 2 | IRS2 | s391 | PVSVAGSPLsPGPVR | 750.3845 | 2 | 6 | 1.087 | 1.324 | 1.609 | 1.783 | 1.4125 | Up | N/A | N/A |
|  |  | s1162 | HSSETFSSTTTVTPVsPSFAHNPK | 876.0656 | 3 | 6 | 1.055 | 0.704 | 1.336 | 0.98 | 1.328 | Up | N/A | N/A |
| IQ motif and SEC7 domain-containing protein 1 | IQSEC1 | s512 | NsWDSPAFSNDVIR | 844.3592 | 2 | 9 | 0.897 | 0.863 | 1.303 | 1.433 | 1.557 | Up | U | [U] Intracellular trafficking, secretion, and vesicular transport |
| Keratin, type I cytoskeletal 18 | KRT18 | s42 | PVSSAASVYAGAGGsGSR | 830.87 | 2 | 12 | 0.308 | 0.339 | 0.438 | 0.561 | 1.5365 | Up | R | [R] General function prediction only |
|  |  | s60 | GGMGsGGLATGIAGGLAGMGGIQNEK | 1171.037 | 2 | 21 | 0.493 | 0.536 | 0.724 | 0.799 | 1.479 | Up | R | [R] General function prediction only |
| Kinesin light chain 4 | KLC4 | s565 | RsSELLVR | 520.2684 | 2 | 4 | 0.714 | 0.864 | 1.229 | 1.258 | 1.589 | Up | Z | [Z] Cytoskeleton |
| KRR1 small subunit processome component homolog | KRR1 | s3 | AsPSLERPEK | 618.287 | 2 | 11 | 0.775 | 0.67 | 1.132 | 1 | 1.478 | Up | JD | [J] Translation, ribosomal structure and biogenesis [D] Cell cycle control, cell division, chromosome partitioning |
| Krueppel-like factor 13 | KLF13 | s162 | ADLEsPQRK | 562.2608 | 2 | 7 | 0.978 | 0.808 | 1.212 | 1.183 | 1.3515 | Up | R | [R] General function prediction only |
| Ladinin-1 | LAD1 | s38 | NLsSTTDDEAPR | 693.2827 | 2 | 6 | 0.901 | 1.204 | 1.26 | 1.485 | 1.3165 | Up | N/A | N/A |
|  |  | s121 | LEAEEGRNsLSPVQATQKPLVSK | N/A | 3 | 9 | 0.346 | 0.379 | 0.488 | 0.519 | 1.3895 | Up | N/A | N/A |
| LIM and SH3 domain protein 1 | LASP1 | s146 | MGPSGGEGMEPERRDsQDGSSYR | 855.6759 | 3 | 13 | 1.007 | 1.02 | 1.97 | 1.704 | 1.8135 | Up | Z | [Z] Cytoskeleton |
| LIM domain only protein 7 | LMO7 | s805 | MYsFDDVLEEGK | 756.8018 | 2 | 18 | 0.42 | 0.524 | 0.771 | 0.924 | 1.7995 | Up | TZR | [T] Signal transduction mechanisms [Z] Cytoskeleton [R] General function prediction only |
|  |  | s1026 | ATLSSTSGLDLMSESGEGEIsPQR | 1266.562 | 2 | 14 | 0.163 | 0.18 | 0.281 | 0.317 | 1.739 | Up | TZR | [T] Signal transduction mechanisms [Z] Cytoskeleton [R] General function prediction only |
|  |  | s1593 | SHsPSASQSGSQLR | 754.8281 | 2 | 6 | 0.26 | 0.168 | 0.39 | 0.25 | 1.4915 | Up | TZR | [T] Signal transduction mechanisms [Z] Cytoskeleton [R] General function prediction only |
| LIM domain-containing protein ajuba | AJUBA | s137 | SSFASSSASDASKPSsPR | 918.394 | 2 | 6 | 0.486 | 0.474 | 0.752 | 0.632 | 1.4405 | Up | T | [T] Signal transduction mechanisms |
|  |  | s230 | HsYPPALGSPGALAGAGVGAAGPLER | 818.404 | 3 | 21 | 0.471 | 0.428 | 0.737 | 0.717 | 1.618 | Up | T | [T] Signal transduction mechanisms |
| Liprin-alpha-1 | PPFIA1 | s239 | RSsDGSLSHEEDLAK | 855.8702 | 2 | 30 | 0.93 | 0.892 | 1.131 | 1.287 | 1.33 | Up | R | [R] General function prediction only |
| Lysine-specific demethylase PHF2 | PHF2 | s705 | RDLsFLLDK | 593.797 | 2 | 5 | 0.764 | 1.129 | 1.074 | 1.542 | 1.3845 | Up | B | [B] Chromatin structure and dynamics |
| MAP7 domain-containing protein 1 | MAP7D1 | s70 | QLPLEPEsPSGQVGPR | 885.9247 | 2 | 7 | 0.753 | 0.964 | 1.272 | 1.579 | 1.663 | Up | N/A | N/A |
| MARVEL domain-containing protein 2 | MARVELD2 | s116 | YISDGVECsPPASPARPNHR | 790.6626 | 3 | 13 | 0.634 | 0.612 | 1.227 | 1.075 | 1.8465 | Up | K | [K] Transcription |
| Methionine--tRNA ligase, cytoplasmic | MARS | s825 | TsPKPAVVETVTTAK | 1030.865 | 2 | 21 | 0.558 | 0.393 | 1.26 | 0.973 | 2.369 | Up | J | [J] Translation, ribosomal structure and biogenesis |
| Microtubule cross-linking factor 1 | MTCL1 | s549 | SLYGDVDsPLPTGEAGGPPSTR | 1127.007 | 2 | 8 | 0.994 | 1.178 | 1.27 | 1.759 | 1.3855 | Up | S | [S] Function unknown |
| Microtubule-actin cross-linking factor 1, isoforms 1/2/3/5 | MACF1 | s1376 | MLsSSDAITQEFMDLR | 962.415 | 2 | 12 | 0.957 | 0.859 | 1.213 | 1.221 | 1.344 | Up | Z | [Z] Cytoskeleton |
| Microtubule-associated protein tau | MAPT | t498 | TPPAPKtPPSSGEPPK | 834.4057 | 2 | 7 | 0.881 | 0.838 | 1.228 | 1.122 | 1.367 | Up | Z | [Z] Cytoskeleton |
| Midasin | MDN1 | s4538 | AEENTDQAsPQEDYAGFER | 1118.937 | 2 | 8 | 0.688 | 0.291 | 1.039 | 0.44 | 1.512 | Up | R | [R] General function prediction only |
| Mitochondrial import receptor subunit TOM22 homolog | TOMM22 | s15 | AAAVAAAGAGEPQsPDELLPK | 1042.999 | 2 | 9 | 0.808 | 0.834 | 1.185 | 1.036 | 1.354 | Up | U | [U] Intracellular trafficking, secretion, and vesicular transport |
| Mitogen-activated protein kinase 14 | MAPK14 | s2 | sQERPTFYR | 653.2848 | 2 | 8 | 0.847 | 0.943 | 1.079 | 1.31 | 1.3305 | Up | T | [T] Signal transduction mechanisms |
| Mitogen-activated protein kinase kinase kinase 7 | MAP3K7 | s389 | MsADMSEIEAR | 660.254 | 2 | 14 | 1.044 | 1.306 | 1.624 | 1.763 | 1.453 | Up | T | [T] Signal transduction mechanisms |
| Mitotic checkpoint serine/threonine-protein kinase BUB1 | BUB1 | s593 | TLAPsPKSPGDFTSAAQLASTPFHK | 906.0855 | 3 | 7 | 0.825 | 0.892 | 1.444 | 1.322 | 1.6155 | Up | D | [D] Cell cycle control, cell division, chromosome partitioning |
|  |  | s596 | sPGDFTSAAQLASTPFHK | 971.4408 | 2 | 16 | 1.109 | 1.153 | 1.589 | 1.441 | 1.3415 | Up | D | [D] Cell cycle control, cell division, chromosome partitioning |
| Mitotic interactor and substrate of PLK1 | MISP | s394 | ALsSDSILSPAPDAR | 790.3718 | 2 | 28 | 0.553 | 0.663 | 0.86 | 0.938 | 1.484 | Up | N/A | N/A |
| Monocarboxylate transporter 4 | SLC16A3 | s436 | LHKPPADsGVDLR | 742.8665 | 2 | 14 | 1.159 | 0.928 | 1.69 | 1.456 | 1.5135 | Up | G | [G] Carbohydrate transport and metabolism |
| Monofunctional C1-tetrahydrofolate synthase, mitochondrial | MTHFD1L | s357 | LQPLsPVPSDIEISR | 865.9399 | 2 | 4 | 0.796 | 0.894 | 1.23 | 1.147 | 1.413 | Up | H | [H] Coenzyme transport and metabolism |
| M-phase inducer phosphatase 2 | CDC25B | s249 | MEVEELsPLALGR | N/A | 2 | 10 | 1.042 | 1.107 | 1.625 | 1.628 | 1.5145 | Up | D | [D] Cell cycle control, cell division, chromosome partitioning |
|  |  | s375 | sLCHDEIENLLDSDHR | 749.9949 | 3 | 18 | 0.605 | 0.8 | 2.293 | 2.761 | 3.625 | Up | D | [D] Cell cycle control, cell division, chromosome partitioning |
| Myc proto-oncogene protein | MYC | s293 | SESGSPSAGGHSKPPHsPLVLK | 746.0301 | 3 | 20 | 0.811 | 0.798 | 1.064 | 1.035 | 1.305 | Up | K | [K] Transcription |
| Myelin protein zero-like protein 1 | MPZL1 | s210 | DYTGCSTSESLsPVK | 855.8501 | 2 | 10 | 0.702 | 0.689 | 0.956 | 0.947 | 1.3695 | Up | N/A | N/A |
| Myocyte-specific enhancer factor 2A | MEF2A | s98 | GCDsPDPDTSYVLTPHTEEK | 1164.472 | 2 | 9 | 1.135 | 0.845 | 1.479 | 1.121 | 1.3145 | Up | K | [K] Transcription |
| Myoferlin | MYOF | s174 | GPVGTVsEAQLAR | 682.8321 | 2 | 6 | 1.067 | 0.651 | 1.582 | 1.176 | 1.6445 | Up | M | [M] Cell wall/membrane/envelope biogenesis |
| Myosin phosphatase Rho-interacting protein | MPRIP | s365 | DFTNEAPPAPLPDASASPLsPHR | 823.0478 | 2 | 19 | 0.961 | 0.907 | 1.204 | 1.224 | 1.3005 | Up | Z | [Z] Cytoskeleton |
| Myristoylated alanine-rich C-kinase substrate | MARCKS | s27 | GEAAAERPGEAAVASsPSK | 932.9255 | 2 | 8 | 0.627 | 0.634 | 0.785 | 0.95 | 1.376 | Up | N/A | N/A |
| Nectin-3 | NECTIN3 | s465 | ESQIDVLQQDELDsYPDSVK | 1194.528 | 2 | 5 | 0.878 | 0.881 | 1.121 | 1.187 | 1.3115 | Up | T | [T] Signal transduction mechanisms |
| Negative elongation factor E | NELFE | s115 | SIsADDDLQESSR | 751.804 | 2 | 6 | 0.964 | 0.726 | 1.768 | 1.203 | 1.7465 | Up | N/A | N/A |
| Nestin | NES | s680 | ENQEPLRsPEVGDEEALRPLTK | 863.0848 | 3 | 4 | 0.665 | 0.821 | 1.233 | 1.342 | 1.743 | Up | N/A | N/A |
| Neurabin-1 | PPP1R9A | s160 | SVHESGQNNRYsPK | 841.8678 | 2 | 8 | 0.642 | 0.571 | 0.923 | 0.839 | 1.4545 | Up | T | [T] Signal transduction mechanisms |
| Neutral amino acid transporter B(0) | SLC1A5 | s493 | TESRsTEPELIQVK | 848.9113 | 2 | 7 | 0.751 | 0.787 | 1.091 | 0.957 | 1.3345 | Up | E | [E] Amino acid transport and metabolism |
| Nipped-B-like protein | NIPBL | s318 | DVPPDILLDsPERK | 837.4109 | 2 | 17 | 0.952 | 0.755 | 1.261 | 1.093 | 1.3865 | Up | BDL | [B] Chromatin structure and dynamics [D] Cell cycle control, cell division, chromosome partitioning [L] Replication, recombination and repair partitioning |
| Nuclear export mediator factor NEMF | NEMF | s831 | KLPsDSGDLEALEGK | 819.8848 | 2 | 10 | 0.908 | 1.125 | 1.548 | 1.601 | 1.564 | Up | R | [R] General function prediction only |
| Nuclear factor 1 C-type | NFIC | s333 | TEMDKsPFNSPSPQDSPR | 1050.441 | 2 | 21 | 0.783 | 0.9 | 1.365 | 1.382 | 1.6405 | Up | K | [K] Transcription |
| Nuclear pore complex protein Nup107 | NUP107 | s86 | QPDISCILGTGGKsPR | 883.4188 | 2 | 12 | 0.701 | 1.023 | 1.011 | 1.272 | 1.3425 | Up | YU | [Y] Nuclear structure [U] Intracellular trafficking, secretion, and vesicular transport |
| Nuclear pore complex protein Nup160 | NUP160 | s1157 | LIRPEYAWIVQPVSGAVYDRPGAsPK | 983.8389 | 3 | 15 | 0.851 | 0.836 | 1.196 | 1.217 | 1.43 | Up | YU | [Y] Nuclear structure [U] Intracellular trafficking, secretion, and vesicular transport |
| Nuclear receptor subfamily 1 group D member 1 | NR1D1 | s280 | SPsPEPTVEDVISQVAR | 945.9459 | 2 | 4 | 0.801 | 0.799 | 1.336 | 1.134 | 1.544 | Up | T | [T] Signal transduction mechanisms |
| Nucleolar GTP-binding protein 1 | GTPBP4 | s468 | TAAGEYDsVSESEDEEMLEIR | 1300.457 | 2 | 10 | 0.8 | 0.679 | 1.236 | 1.059 | 1.553 | Up | R | [R] General function prediction only |
|  |  | s470 | TAAGEYDSVsESEDEEMLEIR | 1300.457 | 2 | 11 | 0.912 | 0.669 | 1.217 | 1.043 | 1.4475 | Up | R | [R] General function prediction only |
|  |  | s472 | TAAGEYDSVSEsEDEEMLEIR | 1300.457 | 2 | 11 | 0.912 | 0.669 | 1.217 | 1.043 | 1.4475 | Up | R | [R] General function prediction only |
| Nucleolar protein 14 | NOP14 | s96 | FGEYNSNMsPEEK | 806.3053 | 2 | 11 | 0.747 | 0.69 | 1.1 | 0.896 | 1.386 | Up | J | [J] Translation, ribosomal structure and biogenesis |
| Nucleolar protein 56 | NOP56 | s570 | KFSKEEPVSsGPEEAVGK | N/A | 2 | 35 | 0.823 | 0.627 | 1.161 | 0.852 | 1.3845 | Up | AJ | [A] RNA processing and modification [J] Translation, ribosomal structure and biogenesis |
| Nucleolar RNA helicase 2 | DDX21 | s71 | KAEPSEVDMNsPK | N/A | 2 | 35 | 0.713 | 0.649 | 1.183 | 1.141 | 1.7085 | Up | A | [A] RNA processing and modification |
| Nucleophosmin | NPM1 | s4 | MEDsMDMDMSPLRPQNYLFGCELK | 1010.413 | 3 | 60 | 0.714 | 0.745 | 1.069 | 0.986 | 1.411 | Up | N/A | N/A |
| Nucleosome assembly protein 1-like 1 | NAP1L1 | s10 | ADIDNKEQsELDQDLDDVEEVEEEETGEETK | 1234.841 | 3 | 8 | 0.418 | 0.537 | 0.653 | 0.847 | 1.569 | Up | BD | [B] Chromatin structure and dynamics [D] Cell cycle control, cell division, chromosome partitioning |
| Opioid growth factor receptor | OGFR | s378 | SQGDEAGGHGEDRPEPLsPK | 714.9744 | 2 | 16 | 1.32 | 1 | 1.722 | 1.553 | 1.429 | Up | N/A | N/A |
| Origin recognition complex subunit 2 | ORC2 | t116 | LASELAKtPQK | 633.3287 | 2 | 8 | 0.389 | 0.588 | 0.547 | 0.772 | 1.3565 | Up | L | [L] Replication, recombination and repair |
| OTU domain-containing protein 4 | OTUD4 | s1024 | VQRPKEESsEDENEVSNILR | 840.0335 | 3 | 19 | 0.592 | 0.567 | 1.401 | 1.409 | 2.4275 | Up | TO | [T] Signal transduction mechanisms [O] Posttranslational modification, protein turnover, chaperones |
| Palmitoyltransferase ZDHHC5 | ZDHHC5 | s380 | GDsLKEPTSIAESSR | 828.8775 | 2 | 13 | 0.763 | 0.843 | 1.163 | 1.16 | 1.4505 | Up | R | [R] General function prediction only |
| PC4 and SFRS1-interacting protein | PSIP1 | t122 | ETSVSKEDtDHEEKASNEDVTK | 853.3641 | 3 | 25 | 0.719 | 0.77 | 1.216 | 1.214 | 1.633 | Up | K | [K] Transcription |
| Periphilin-1 | PPHLN1 | s121 | ERsPYKR | 508.2397 | 2 | 8 | 0.701 | 0.606 | 1.026 | 0.761 | 1.36 | Up | N/A | N/A |
| Phosphatidate phosphatase LPIN3 | LPIN3 | s411 | RWsEPSSQK | 592.7584 | 2 | 4 | 0.723 | 0.783 | 1.197 | 1.36 | 1.696 | Up | NI | [N] Cell motility [I] Lipid transport and metabolism |
| Pinin | PNN | s381 | QQDsQPEEVMDVLEMVENVK | 1214.026 | 2 | 28 | 0.436 | 0.431 | 0.715 | 0.796 | 1.741 | Up | Z | [Z] Cytoskeleton |
|  |  | s552 | LTEVPVEPVLTVHPEsK | 977.5003 | 2 | 8 | 0.69 | 0.9 | 1.045 | 1.237 | 1.445 | Up | Z | [Z] Cytoskeleton |
| Pleckstrin homology domain-containing family G member 3 | PLEKHG3 | s1040 | SPLsPTETFSWPDVR | 939.8892 | 2 | 13 | 0.94 | 0.731 | 1.431 | 0.973 | 1.4275 | Up | R | [R] General function prediction only |
| Pleckstrin homology-like domain family B member 2 | PHLDB2 | s334 | TSASEGNPYVSSTLSVPAsPR | 1094.002 | 2 | 8 | 0.897 | 1.027 | 1.174 | 1.398 | 1.3355 | Up | Z | [Z] Cytoskeleton |
| Prelamin-A/C | LMNA | s301 | IDsLSAQLSQLQK | 755.8793 | 2 | 7 | 1.1 | 1.088 | 1.584 | 1.444 | 1.3845 | Up | DY | [D] Cell cycle control, cell division, chromosome partitioning [Y] Nuclear structure |
|  |  | s458 | NKsNEDQSMGNWQIK | 929.8931 | 2 | 8 | 0.823 | 0.662 | 1.409 | 1.108 | 1.692 | Up | DY | [D] Cell cycle control, cell division, chromosome partitioning [Y] Nuclear structure |
|  |  | s628 | sVGGSGGGSFGDNLVTR | 823.8621 | 2 | 8 | 1.014 | 1.135 | 1.605 | 1.975 | 1.662 | Up | DY | [D] Cell cycle control, cell division, chromosome partitioning [Y] Nuclear structure |
| Probable cation-transporting ATPase 13A3 | ATP13A3 | s817 | LVHDsLEDLQMTR | 818.8737 | 2 | 16 | 0.97 | 0.926 | 1.471 | 1.462 | 1.548 | Up | P | [P] Inorganic ion transport and metabolism |
| Probable helicase senataxin | SETX | s911 | KSsPFKDLMTVPESR | 901.4313 | 2 | 13 | 1.048 | 1.013 | 1.377 | 1.318 | 1.307 | Up | A | [A] RNA processing and modification |
| Proliferation marker protein Ki-67 | MKI67 | s308 | SGGSGHAVAEPAsPEQELDQNK | 1144.495 | 2 | 18 | 1.133 | 0.707 | 1.397 | 0.988 | 1.315 | Up | N/A | N/A |
|  |  | t1355 | ELFQtPGHTEEAVAAGK | 932.9275 | 2 | 8 | 0.526 | 0.966 | 0.815 | 1.33 | 1.463 | Up | N/A | N/A |
|  |  | t1503 | SQPDPVDtPTSSKPQSK | 939.9277 | 2 | 8 | 0.673 | 0.781 | 0.906 | 1.105 | 1.3815 | Up | N/A | N/A |
| Proliferation-associated protein 2G4 | PA2G4 | s2 | sGEDEQQEQTIAEDLVVTK | 1120.994 | 2 | 19 | 0.536 | 0.564 | 1.062 | 1.098 | 1.9635 | Up | R | [R] General function prediction only |
| Proline and serine-rich protein 2 | PROSER2 | s179 | APsPPVEHPR | 583.7713 | 2 | 12 | 0.936 | 0.923 | 1.35 | 1.535 | 1.5535 | Up | N/A | N/A |
|  |  | s312 | AQVLATIHGHAGAFPAAGDAGEGAPGGGSsPER | 1031.811 | 3 | 12 | 0.985 | 0.954 | 1.335 | 1.404 | 1.413 | Up | N/A | N/A |
| Proteasome subunit alpha type-3 | PSMA3 | s250 | EEDEsDDDNM | N/A | 1 | 16 | 0.553 | 0.503 | 0.739 | 0.781 | 1.445 | Up | O | [O] Posttranslational modification, protein turnover, chaperones |
| Protein arginine N-methyltransferase 3 | PRMT3 | s25 | GAVENEEDLPELsDSGDEAAWEDEDDADLPHGK | 1238.81 | 3 | 12 | 0.798 | 0.892 | 1.188 | 1.239 | 1.4395 | Up | OKT | [O] Posttranslational modification, protein turnover, chaperones [K] Transcription [T] Signal transduction mechanisms |
|  |  | s27 | GAVENEEDLPELSDsGDEAAWEDEDDADLPHGK | 1238.81 | 3 | 12 | 0.798 | 0.892 | 1.188 | 1.239 | 1.4395 | Up | OKT | [O] Posttranslational modification, protein turnover, chaperones [K] Transcription [T] Signal transduction mechanisms |
| Protein DEK | DEK | t13 | SASAPAAEGEGtPTQPASEK | 1004.431 | 2 | 8 | 0.648 | 0.421 | 0.813 | 0.584 | 1.32 | Up | B | [B] Chromatin structure and dynamics |
| Protein ELYS | AHCTF1 | s1541 | NLsFNELYPSGTLK | 831.8924 | 2 | 9 | 0.678 | 0.806 | 1.055 | 1.211 | 1.529 | Up | N/A | N/A |
| Protein FAM122B | FAM122B | s58 | HsLEEGLDMVNR | 740.3185 | 2 | 9 | 1.301 | 1.349 | 1.878 | 1.707 | 1.3535 | Up | N/A | N/A |
| Protein FAM171A1 | FAM171A1 | s849 | TADAPSEPAAsPHQR | 807.849 | 2 | 8 | 1.475 | 1.279 | 1.908 | 1.903 | 1.3915 | Up | N/A | N/A |
| Protein FAM83H | FAM83H | s411 | RHsFATEGAGAVENFAAAR | 681.3128 | 3 | 8 | 0.561 | 0.531 | 0.674 | 0.767 | 1.3225 | Up | N/A | N/A |
| Protein ITPRID2 | ITPRID2 | s1161 | ASVALTPTAPsR | 625.8107 | 2 | 6 | 1.039 | 0.965 | 1.295 | 1.386 | 1.3415 | Up | N/A | N/A |
| Protein IWS1 homolog | IWS1 | s511 | EAEDsDSDDNIKR | 827.2875 | 2 | 6 | 0.655 | 0.72 | 0.946 | 0.941 | 1.375 | Up | S | [S] Function unknown |
|  |  | s513 | EAEDSDsDDNIKR | 827.2875 | 2 | 7 | 0.736 | 0.809 | 1.063 | 1.056 | 1.375 | Up | S | [S] Function unknown |
| Protein phosphatase inhibitor 2 | PPP1R2 | s121 | IQEQEsSGEEDSDLSPEER | 1162.428 | 2 | 9 | 0.972 | 0.473 | 1.648 | 0.715 | 1.6035 | Up | OT | [O] Posttranslational modification, protein turnover, chaperones [T] Signal transduction mechanisms |
| Protein PRRC2A | PRRC2A | s456 | KQsSSEISLAVER | 757.3665 | 2 | 8 | 0.945 | 1.124 | 1.313 | 1.51 | 1.3665 | Up | X | none |
| Protein Shroom3 | SHROOM3 | s910 | DRPGsPESPLLDAPFSR | 960.9462 | 2 | 19 | 0.183 | 0.209 | 0.49 | 0.523 | 2.5965 | Up | N/A | N/A |
| Protein timeless homolog | TIMELESS | s1173 | QLLDsDEEQEEDEGR | 936.3626 | 2 | 8 | 0.9 | 0.544 | 1.396 | 0.808 | 1.518 | Up | L | [L] Replication, recombination and repair |
| Protein transport protein Sec31A | SEC31A | s1163 | EQTLsPTITSGLHNIAR | 959.4751 | 2 | 5 | 0.811 | 0.961 | 1.135 | 1.161 | 1.3035 | Up | U | [U] Intracellular trafficking, secretion, and vesicular transport |
| Protein unc-93 homolog B1 | UNC93B1 | s550 | YLEEDNSDEsDAEGEHGDGAEEEAPPAGPR | 1111.407 | 3 | 11 | 0.856 | 0.685 | 1.264 | 1.127 | 1.561 | Up | S | [S] Function unknown |
| Protocadherin-1 | PCDH1 | s962 | IHLPLNYPPGsPDLGR | 913.4535 | 2 | 20 | 1.043 | 1.045 | 1.669 | 1.76 | 1.6425 | Up | S | [S] Function unknown |
| Putative RNA-binding protein Luc7-like 2 | LUC7L2 | s347 | FRDQDLAsCDRDR | 578.5736 | 2 | 11 | 0.848 | 0.801 | 1.21 | 1.001 | 1.3395 | Up | A | [A] RNA processing and modification |
| Rac GTPase-activating protein 1 | RACGAP1 | s203 | sIGSAVDQGNESIVAK | 827.8878 | 2 | 8 | 0.754 | 0.658 | 1.405 | 1.337 | 1.948 | Up | R | [R] General function prediction only |
| Ral GTPase-activating protein subunit beta | RALGAPB | t734 | TNSGISSASGGSTEPTtPDSERPAQALLR | 989.7946 | 3 | 4 | 0.804 | 0.897 | 1.218 | 1.15 | 1.398 | Up | S | [S] Function unknown |
| RANBP2-like and GRIP domain-containing protein 5/6 | RGPD5 | s796 | SYKYsPETPPR | 702.8134 | 2 | 8 | 0.892 | 0.658 | 1.268 | 0.91 | 1.402 | Up | U | [U] Intracellular trafficking, secretion, and vesicular transport |
| Ras GTPase-activating protein-binding protein 1 | G3BP1 | s149 | YQDEVFGGFVTEPQEEsEEEVEEPEER | 1099.449 | 3 | 18 | 0.766 | 0.765 | 1.098 | 1.076 | 1.4205 | Up | T | [T] Signal transduction mechanisms |
| Ras-related GTP-binding protein C | RRAGC | s15 | SLQYGAEETPLAGsYGAADSFPK | 1241.047 | 2 | 17 | 0.575 | 0.798 | 0.807 | 1.002 | 1.3305 | Up | U | [U] Intracellular trafficking, secretion, and vesicular transport |
| Regulation of nuclear pre-mRNA domain-containing protein 2 | RPRD2 | s665 | LESESTsPSLEMK | 759.3257 | 2 | 9 | 0.854 | 0.758 | 1.279 | 0.952 | 1.3765 | Up | A | [A] RNA processing and modification |
| RelA-associated inhibitor | PPP1R13L | s280 | PAsPSLQLLPWR | 722.8711 | 2 | 7 | 0.911 | 1.163 | 1.169 | 1.57 | 1.317 | Up | D | [D] Cell cycle control, cell division, chromosome partitioning |
| Reticulon-3 | RTN3 | s229 | VEGIYTYSLsPSK | 762.3551 | 2 | 6 | 0.794 | 0.722 | 1.221 | 1.207 | 1.6035 | Up | U | [U] Intracellular trafficking, secretion, and vesicular transport |
| Reticulophagy regulator 3 | RETREG3 | t440 | SPSSDLDtDAEGDDFELLDQSELSQLDPASSR | 1200.487 | 3 | 8 | 0.387 | 0.428 | 0.864 | 0.942 | 2.218 | Up | N/A | N/A |
| Retinoblastoma-associated protein | RB1 | t826 | ISEGLPTPTKMtPR | 804.3968 | 2 | 15 | 0.564 | 0.675 | 1.77 | 2.286 | 3.262 | Up | D | [D] Cell cycle control, cell division, chromosome partitioning |
| Retinoblastoma-like protein 1 | RBL1 | s640 | DMQPLsPISVHER | 794.8631 | 2 | 9 | 0.982 | 1.015 | 1.282 | 1.415 | 1.3505 | Up | D | [D] Cell cycle control, cell division, chromosome partitioning |
|  |  | s1041 | VIAIDSDAEsPAKR | 776.3744 | 2 | 14 | 1.036 | 1.173 | 1.404 | 1.619 | 1.368 | Up | D | [D] Cell cycle control, cell division, chromosome partitioning |
| Rho GTPase-activating protein 29 | ARHGAP29 | s357 | AEEEHLSSsGGLAK | 747.8272 | 2 | 5 | 1.54 | 0.599 | 1.968 | 0.933 | 1.417 | Up | T | [T] Signal transduction mechanisms |
| Ribonuclease H1 | RNASEH1 | s76 | SAsPEVSEGHENQHGQESEAK | 815.682 | 3 | 16 | 0.963 | 0.711 | 1.33 | 0.928 | 1.344 | Up | L | [L] Replication, recombination and repair |
| RNA cytidine acetyltransferase | NAT10 | s934 | TLsDDLDEAAK | 629.2659 | 2 | 7 | 0.662 | 0.6 | 1.338 | 1.338 | 2.126 | Up | R | [R] General function prediction only |
| RNA polymerase II elongation factor ELL | ELL | s442 | LGLPLLTDCAQPSRPHGsPSR | 780.3824 | 3 | 8 | 0.909 | 0.874 | 1.238 | 1.155 | 1.341 | Up | K | [K] Transcription |
| RNA-binding motif protein, X chromosome | RBMX | s277 | DRDYSDHPsGGSYR | 846.3259 | 2 | 14 | 0.796 | 0.805 | 1.175 | 0.973 | 1.342 | Up | R | [R] General function prediction only |
| RNA-binding protein 10 | RBM10 | s89 | HsPTGPPGFPR | 615.2768 | 2 | 31 | 0.725 | 0.767 | 0.987 | 1.067 | 1.376 | Up | R | [R] General function prediction only |
|  |  | s797 | RAHLsENELEALEK | N/A | 3 | 12 | 0.731 | 0.706 | 1.399 | 1.32 | 1.8915 | Up | R | [R] General function prediction only |
| RNA-binding protein 12B | RBM12B | s98 | GRPGsGTSGVDSLSNFIESVK | 1087.51 | 3 | 15 | 1.107 | 1.14 | 1.576 | 1.794 | 1.499 | Up | R | [R] General function prediction only |
|  |  | s254 | EGDVLRRSEEHsPPR | 641.9418 | 3 | 12 | 0.866 | 0.788 | 1.833 | 1.643 | 2.102 | Up | R | [R] General function prediction only |
| RNA-binding protein 5 | RBM5 | s78 | SEDGYHsDGDYGEHDYR | 694.5764 | 3 | 31 | 0.657 | 0.528 | 0.88 | 0.7 | 1.332 | Up | R | [R] General function prediction only |
| RUS1 family protein C16orf58 | C16orf58 | s462 | ATWLLsPEKK | 626.8205 | 2 | 4 | 0.848 | 0.842 | 1.139 | 1.238 | 1.408 | Up | S | [S] Function unknown |
| Sarcolemmal membrane-associated protein | SLMAP | s452 | ESDFSDTLsPSKEK | 825.3507 | 2 | 5 | 0.829 | 0.793 | 1.174 | 0.968 | 1.318 | Up | T | [T] Signal transduction mechanisms |
| Sentrin-specific protease 3 | SENP3 | s169 | NHLsPQQGGATPQVPSPCCR | 757.6647 | 3 | 24 | 0.629 | 0.378 | 1.244 | 0.716 | 1.9375 | Up | O | [O] Posttranslational modification, protein turnover, chaperones |
| Serine/arginine repetitive matrix protein 1 | SRRM1 | s242 | VKEPSVQEATSTsDILK | 956.4692 | 2 | 5 | 0.675 | 0.68 | 1.223 | 1.126 | 1.7335 | Up | AR | [A] RNA processing and modification [R] General function prediction only |
| Serine/arginine repetitive matrix protein 2 | SRRM2 | s333 | QPSsPYEDKDKDK | 808.8456 | 2 | 11 | 0.945 | 0.654 | 1.351 | 1.022 | 1.497 | Up | A | [A] RNA processing and modification |
|  |  | s950 | RsRSVSPCSNVESR | 930.8467 | 2 | 13 | 0.819 | 0.591 | 1.585 | 1.125 | 1.9185 | Up | A | [A] RNA processing and modification |
|  |  | s954 | SVsPCSNVESR | 930.8467 | 2 | 21 | 0.888 | 0.653 | 1.591 | 1.12 | 1.753 | Up | A | [A] RNA processing and modification |
| Serine/threonine-protein kinase MRCK gamma | CDC42BPG | s1482 | GSGPQRPHsFSEALR | 569.2651 | 2 | 14 | 0.662 | 0.517 | 1.107 | 0.749 | 1.5615 | Up | T | [T] Signal transduction mechanisms |
| Serine/threonine-protein kinase Mtor | MTOR | s2448 | TDsYSAGQSVEILDGVELGEPAHK | 861.3934 | 3 | 11 | 0.877 | 0.948 | 1.331 | 1.21 | 1.397 | Up | L | [L] Replication, recombination and repair |
| Serine/threonine-protein phosphatase 2A 56 kDa regulatory subunit delta isoform | PPP2R5D | s89 | RQSsSRFNLSK | 735.3185 | 2 | 9 | 0.768 | 1.103 | 1.146 | 1.514 | 1.433 | Up | T | [T] Signal transduction mechanisms |
|  |  | s90 | RQSSsRFNLSK | 735.3185 | 2 | 8 | 0.768 | 1.103 | 1.146 | 1.514 | 1.433 | Up | T | [T] Signal transduction mechanisms |
| Sex comb on midleg-like protein 2 | SCML2 | s267 | TESSPSEASQHSMQsPQK | 1013.415 | 2 | 4 | 0.781 | 0.784 | 1.309 | 1.194 | 1.6005 | Up | K | [K] Transcription |
|  |  | s511 | RSPQQTVPYVVPLsPK | N/A | 2 | 25 | 0.525 | 0.492 | 1.523 | 1.415 | 2.8855 | Up | K | [K] Transcription |
| SH3 domain-containing kinase-binding protein 1 | SH3KBP1 | s230 | sIEVENDFLPVEK | 799.8711 | 2 | 8 | 0.867 | 0.839 | 1.354 | 1.598 | 1.7325 | Up | T | [T] Signal transduction mechanisms |
|  |  | s587 | ANsPSLFGTEGKPK | 756.8583 | 2 | 8 | 0.757 | 0.779 | 1.027 | 1.122 | 1.3985 | Up | T | [T] Signal transduction mechanisms |
| Signal-induced proliferation-associated 1-like protein 3 | SIPA1L3 | s1544 | TLsDESLCSGR | 652.763 | 2 | 9 | 0.945 | 0.492 | 1.242 | 0.635 | 1.3015 | Up | T | [T] Signal transduction mechanisms |
| Signal-induced proliferation-associated protein 1 | SIPA1 | s839 | TEFLHSQNSLsPR | 798.3643 | 2 | 7 | 1.085 | 0.969 | 1.41 | 1.495 | 1.421 | Up | T | [T] Signal transduction mechanisms |
| Sister chromatid cohesion protein PDS5 homolog B | PDS5B | s1283 | LKEDILENEDEQNsPPKK | 1039.47 | 3 | 37 | 0.493 | 0.432 | 0.754 | 0.735 | 1.6145 | Up | D | [D] Cell cycle control, cell division, chromosome partitioning |
| Sodium-dependent phosphate transporter 2 | SLC20A2 | s256 | EGALSRVsDESLSK | 918.0875 | 2 | 15 | 0.935 | 0.564 | 1.165 | 0.784 | 1.319 | Up | P | [P] Inorganic ion transport and metabolism |
|  |  | s259 | EGALSRVSDEsLSKVQEAESPVFK | 918.0875 | 3 | 14 | 0.963 | 0.58 | 1.201 | 0.807 | 1.319 | Up | P | [P] Inorganic ion transport and metabolism |
| Soluble lamin-associated protein of 75 kDa | FAM169A | s526 | AHLGsSDNVATMSNEER | 949.3909 | 2 | 7 | 0.726 | 0.773 | 1.156 | 1.01 | 1.4495 | Up | N/A | N/A |
| Sororin | CDCA5 | s209 | VCAKPWAPDMTLPGIsPPPEK | 791.0457 | 3 | 7 | 0.888 | 0.928 | 1.131 | 1.328 | 1.3515 | Up | N/A | N/A |
| Spindlin-1 | SPIN1 | s124 | IsDAHLADTMIGK | 726.3336 | 2 | 7 | 1.004 | 1.013 | 1.658 | 1.667 | 1.648 | Up | N/A | N/A |
| Splicing factor 45 | RBM17 | s169 | sMGGAAIAPPTSLVEK | 804.8888 | 2 | 7 | 0.946 | 1.206 | 1.492 | 1.822 | 1.544 | Up | A | [A] RNA processing and modification |
| SR-related and CTD-associated factor 8 | SCAF8 | s617 | SSEPVKETVQTTQsPTPVEK | 1126.538 | 3 | 22 | 0.715 | 0.721 | 1.28 | 1.318 | 1.808 | Up | AK | [A] RNA processing and modification [K] Transcription |
| Stathmin | STMN1 | s46 | KKDLsLEEIQK | N/A | 2 | 12 | 1.194 | 1.241 | 1.854 | 1.97 | 1.57 | Up | N/A | N/A |
| Stress-70 protein, mitochondrial | HSPA9 | s162 | LYsPSQIGAFVLMK | 817.4066 | 2 | 8 | 1.04 | 0.958 | 1.248 | 1.374 | 1.317 | Up | O | [O] Posttranslational modification, protein turnover, chaperones |
| SUN domain-containing ossification factor | SUCO | s1103 | EVDPNDLYIVEPLKFsPEK | 814.0731 | 2 | 19 | 0.421 | 0.438 | 0.566 | 0.583 | 1.339 | Up | S | [S] Function unknown |
| SWI/SNF-related matrix-associated actin-dependent regulator of chromatin subfamily A containing DEAD/H box 1 | SMARCAD1 | s152 | RNDDISELEDLsELEDLKDAK | 843.0531 | 3 | 13 | 0.933 | 1.125 | 1.652 | 1.825 | 1.696 | Up | B | [B] Chromatin structure and dynamics |
| Symplekin | SYMPK | t1257 | SPQTLAPVGEDAMKtPSPAAEDAREPEAK | 1051.8 | 4 | 11 | 1.047 | 0.889 | 1.402 | 1.347 | 1.4265 | Up | A | [A] RNA processing and modification |
|  |  | s1259 | SPQTLAPVGEDAMKTPsPAAEDAREPEAK | N/A | 3 | 17 | 0.831 | 0.716 | 1.113 | 1.159 | 1.4785 | Up | A | [A] RNA processing and modification |
| Synaptosomal-associated protein 23 | SNAP23 | s110 | TTWGDGGENsPCNVVSK | 944.3826 | 2 | 8 | 0.312 | 0.173 | 0.522 | 0.286 | 1.6675 | Up | U | [U] Intracellular trafficking, secretion, and vesicular transport |
| Syntabulin GN=SYBU | SYBU | s50 | VSPASESPFsEEESR | 859.3513 | 2 | 8 | 0.528 | 0.51 | 0.761 | 0.761 | 1.466 | Up | N/A | N/A |
| TATA element modulatory factor | TMF1 | s344 | SVSEINsDDELSGK | 780.3273 | 2 | 7 | 0.577 | 0.562 | 0.793 | 0.754 | 1.3585 | Up | K | [K] Transcription |
| T-complex protein 1 subunit theta | CCT8 | s23 | HFsGLEEAVYR | 694.3057 | 2 | 7 | 0.995 | 1.022 | 1.307 | 1.335 | 1.3115 | Up | O | [O] Posttranslational modification, protein turnover, chaperones |
| Telomerase Cajal body protein 1 | WRAP53 | s26 | TLETQPLAPDCCPSDQDPAPAHPsPHASPMNK | 1182.842 | 3 | 15 | 1.094 | 1.09 | 1.461 | 1.489 | 1.352 | Up | R | [R] General function prediction only |
|  |  | s30 | TLETQPLAPDCCPSDQDPAPAHPSPHAsPMNK | 1209.498 | 3 | 7 | 0.764 | 0.907 | 1.33 | 1.437 | 1.663 | Up | R | [R] General function prediction only |
| THUMP domain-containing protein 1 | THUMPD1 | s86 | FTDKDQQPsGSEGEDDDAEAALKK | 887.7115 | 3 | 47 | 0.78 | 0.51 | 1.276 | 0.826 | 1.6285 | Up | R | [R] General function prediction only |
| Tight junction protein ZO-2 | TJP2 | s130 | KVQVAALQAsPPLDQDDR | 1015.999 | 2 | 16 | 0.574 | 0.515 | 0.783 | 0.79 | 1.4505 | Up | T | [T] Signal transduction mechanisms |
|  |  | s430 | HQYSDYDYHsSSEKLK | 716.2767 | 3 | 48 | 0.467 | 0.387 | 0.656 | 0.513 | 1.3655 | Up | T | [T] Signal transduction mechanisms |
| TP53-binding protein 1 | TP53BP1 | s1068 | GNLLHFPSsQGEEEKEKLEGDHTIR | 733.3475 | 4 | 23 | 0.345 | 0.28 | 0.579 | 0.412 | 1.576 | Up | L | [L] Replication, recombination and repair |
| Transcription factor Sp2 | SP2 | s78 | LVPIKPAPLPLsPGK | 803.9702 | 2 | 20 | 0.68 | 0.656 | 1.028 | 0.968 | 1.4935 | Up | R | [R] General function prediction only |
| Transcription factor Sp3 | SP3 | s73 | IGPPsPGDDEEEAAAAAGAPAAAGATGDLASAQLGGAPNR | 1217.89 | 3 | 16 | 0.803 | 0.722 | 1.038 | 0.963 | 1.3125 | Up | R | [R] General function prediction only |
| Transcription intermediary factor 1-beta | TRIM28 | s50 | STAPSAAASASASAAASsPAGGGAEALELLEHCGVCR | 1174.859 | 3 | 28 | 0.871 | 0.986 | 1.229 | 1.263 | 1.3455 | Up | O | [O] Posttranslational modification, protein turnover, chaperones |
| Transcriptional repressor CTCF | CTCF | s402 | THsGEKPYECYICHAR | 696.6203 | 3 | 6 | 0.688 | 0.886 | 1.083 | 1.476 | 1.6195 | Up | R | [R] General function prediction only |
| Transcriptional repressor p66-beta | GATAD2B | s129 | GRLTPSPDIIVLsDNEASSPR | N/A | 3 | 28 | 0.849 | 1.297 | 1.184 | 1.595 | 1.312 | Up | S | [S] Function unknown |
|  |  | s135 | LTPSPDIIVLSDNEASsPR | 1046.004 | 2 | 21 | 0.66 | 0.855 | 0.937 | 1.283 | 1.4595 | Up | S | [S] Function unknown |
| Transformer-2 protein homolog alpha | TRA2A | s236 | RDsYYDR | N/A | 2 | 12 | 0.591 | 0.721 | 1.102 | 1.22 | 1.778 | Up | R | [R] General function prediction only |
| Transforming acidic coiled-coil-containing protein 2 | TACC2 | s2569 | MSEsPTPCSGSSFEETEALVNTAAK | 904.0468 | 2 | 14 | 0.549 | 0.667 | 0.759 | 1.052 | 1.48 | Up | N/A | N/A |
| Transmembrane protein 201 | TMEM201 | s454 | ALsLGTIPSLTR | 654.8498 | 2 | 7 | 0.719 | 1.147 | 1.146 | 1.54 | 1.469 | Up | S | [S] Function unknown |
| Transmembrane protein 40 | TMEM40 | s137 | RGsDPASGEVEASQLR | N/A | 2 | 11 | 0.777 | 0.771 | 1.204 | 1.313 | 1.625 | Up | N/A | N/A |
| Tyrosine-protein kinase Lyn | LYN | s13 | GKDSLsDDGVDLK | 714.8163 | 2 | 21 | 0.803 | 0.494 | 1.17 | 0.719 | 1.4565 | Up | T | [T] Signal transduction mechanisms |
| Tyrosine-protein phosphatase non-receptor type 14 | PTPN14 | s578 | PATsTPDLASHR | N/A | 2 | 14 | 0.786 | 0.721 | 1.174 | 1.123 | 1.5245 | Up | T | [T] Signal transduction mechanisms |
| Tyrosine-protein phosphatase non-receptor type 2 | PTPN2 | s304 | ELSKEDLSPAFDHsPNK | 997.4488 | 3 | 30 | 0.58 | 0.529 | 1.089 | 1.208 | 2.0805 | Up | T | [T] Signal transduction mechanisms |
| U3 small nucleolar RNA-associated protein 14 homolog A | UTP14A | s453 | DSGsQEVLSELR | 700.3087 | 2 | 8 | 0.581 | 0.6 | 0.78 | 0.761 | 1.3045 | Up | S | [S] Function unknown |
| Ubiquitin carboxyl-terminal hydrolase 36 | USP36 | s682 | SPVLSNTTTEPASTMsPPPAK | 1097.011 | 2 | 12 | 0.983 | 0.845 | 1.435 | 1.06 | 1.3575 | Up | O | [O] Posttranslational modification, protein turnover, chaperones |
|  |  | s952 | HSCSPMGDGDPEAMEEsPR | 1084.89 | 2 | 18 | 0.831 | 0.738 | 1.331 | 1.133 | 1.5685 | Up | O | [O] Posttranslational modification, protein turnover, chaperones |
| Ubiquitin recognition factor in ER-associated degradation protein 1 | UFD1 | s247 | GVEPSPsPIKPGDIK | 800.9027 | 2 | 10 | 0.878 | 0.826 | 1.256 | 0.998 | 1.319 | Up | O | [O] Posttranslational modification, protein turnover, chaperones |
| Ubiquitin-associated protein 2-like | UBAP2L | s467 | STSAPQMsPGSSDNQSSSPQPAQQK | 1306.55 | 2 | 16 | 0.923 | 0.734 | 1.185 | 0.977 | 1.3075 | Up | N/A | N/A |
| Uncharacterized protein C1orf226 | C1orf226 | s249 | LSLSPISLAESWEDGsPPPQAR | 1209.073 | 2 | 18 | 0.838 | 0.743 | 1.638 | 1.483 | 1.9755 | Up | N/A | N/A |
| Uncharacterized protein KIAA1671 | KIAA1671 | s465 | EDSTLALAVGSESPLATPAsPSAAPEPEK | 968.125 | 3 | 6 | 1.046 | 0.857 | 1.461 | 1.233 | 1.417 | Up | N/A | N/A |
|  |  | s1063 | KITPPSsPHSLTSTLVSLGHEEALEMAGSK | 816.8869 | 4 | 8 | 1.084 | 0.825 | 1.465 | 1.302 | 1.464 | Up | N/A | N/A |
| Unconventional myosin-XVIIIa | MYO18A | s2043 | YSHSYLSDsDTEAK | 841.8327 | 2 | 16 | 0.39 | 0.311 | 0.576 | 0.512 | 1.561 | Up | Z | [Z] Cytoskeleton |
| Vacuolar protein sorting-associated protein 13C | VPS13C | s3641 | YHCAIPGsKK | 620.7808 | 2 | 8 | 0.61 | 0.773 | 0.722 | 1.099 | 1.302 | Up | U | [U] Intracellular trafficking, secretion, and vesicular transport |
| Vascular endothelial zinc finger 1 | VEZF1 | s197 | LSHsDEKPFECPICNQR | 732.9783 | 3 | 6 | 0.718 | 0.826 | 1.065 | 1.272 | 1.51 | Up | R | [R] General function prediction only |
| WD repeat-containing protein 26 | WDR26 | s121 | RLsQSDEDVIR | 699.3247 | 2 | 18 | 0.843 | 0.822 | 1.459 | 1.179 | 1.5825 | Up | S | [S] Function unknown |
| WD repeat-containing protein 43 | WDR43 | s431 | RKsGGNEVSIEER | 770.8594 | 2 | 16 | 0.533 | 0.742 | 0.967 | 1.196 | 1.712 | Up | R | [R] General function prediction only |
| WD repeat-containing protein 55 | WDR55 | s14 | TCEERPAEDGsDEEDPDSMEAPTR | 935.0163 | 3 | 14 | 0.593 | 0.613 | 0.85 | 0.754 | 1.3325 | Up | R | [R] General function prediction only |
| WD repeat-containing protein 74 | WDR74 | s361 | KLsGLEQPQGALQTR | 853.4353 | 2 | 6 | 0.924 | 1.153 | 1.605 | 1.678 | 1.5955 | Up | S | [S] Function unknown |
| Zinc finger and BTB domain-containing protein 20 | ZBTB20 | t305 | YLSTtPETTHCR | 773.3238 | 2 | 4 | 0.893 | 0.847 | 1.275 | 1.058 | 1.3385 | Up | R | [R] General function prediction only |
| Zinc finger C3H1 domain-containing protein | ZFC3H1 | s1046 | sFLESNYFTKPNLK | 884.4213 | 2 | 8 | 0.997 | 0.915 | 1.479 | 1.444 | 1.5315 | Up | S | [S] Function unknown |
|  |  | s1304 | KPISDNSFSsDEEQSTGPIK | 1163.48 | 2 | 9 | 0.913 | 0.818 | 1.233 | 1.29 | 1.463 | Up | S | [S] Function unknown |
| Zinc finger CCCH domain-containing protein 13 | ZC3H13 | s993 | GNIETTSEDGQVFsPK | 894.888 | 2 | 22 | 0.76 | 0.778 | 1.092 | 1.014 | 1.3695 | Up | K | [K] Transcription |
| Zinc finger CCCH domain-containing protein 4 | ZC3H4 | s908 | ALPTSKPEGSLHSsPVGPSSSK | 1115.541 | 2 | 15 | 0.486 | 0.429 | 0.912 | 0.813 | 1.8835 | Up | A | [A] RNA processing and modification |
| Zinc finger CCCH-type antiviral protein 1 | ZC3HAV1 | t273 | SCtPSPDQISHR | 772.7837 | 2 | 8 | 0.78 | 0.552 | 1.022 | 0.765 | 1.3475 | Up | N/A | N/A |
| Zinc finger protein 106 | ZNF106 | s937 | RHsAQLSSDHIIPLMHLAK | 745.3803 | 4 | 9 | 0.464 | 0.532 | 1.676 | 1.641 | 3.345 | Up | R | [R] General function prediction only |
| Zinc finger protein 185 | ZNF185 | t447 | GGQGDPAVPAQQPADPStPER | 1077.976 | 2 | 4 | 0.807 | 0.7 | 1.35 | 1.212 | 1.7025 | Up | TZR | [T] Signal transduction mechanisms [Z] Cytoskeleton [R] General function prediction only |
| Zinc finger protein 638 | ZNF638 | s508 | sRSPMHYMYR | 744.2717 | 2 | 36 | 0.504 | 0.505 | 0.791 | 0.73 | 1.505 | Up | N/A | N/A |
|  |  | s510 | SRsPMHYMYR | 744.2717 | 2 | 38 | 0.523 | 0.46 | 0.701 | 0.708 | 1.4395 | Up | N/A | N/A |
| Zinc finger protein with KRAB and SCAN domains 1 | ZKSCAN1 | s13 | EATGLsPQAAQEK | 705.319 | 2 | 7 | 0.893 | 0.606 | 1.505 | 1.135 | 1.7785 | Up | R | [R] General function prediction only |
| Zinc transporter ZIP10 | SLC39A10 | s591 | LNETELTDLEGQQEsPPK | 1096.162 | 2 | 10 | 0.9 | 0.853 | 1.195 | 1.239 | 1.3905 | Up | P | [P] Inorganic ion transport and metabolism |

**Table S3. List of primers**

| **Primer name** | **Primer sequences（5’ to 3’）** | |
| --- | --- | --- |
| **Primers for** **endogenously PIK3CA/p110α C-terminal epitope tag knock-in (pAAV backbone)** | | |
|  | Left arm Forward  Left arm Reverse | GGGAAAG/ideoxyU/tttgaggaaagtcagtcaacc  GGAGACA/ideoxyU/GCGTTCAATGCATGCTGTTTAATTG |
| PIK3CA locus  C-terminal epitope tag KI | Right arm Forward  Right arm Reverse | GGTCCCA/ideoxyU/AAAGATAACTGAGAAAATGAAAG  GGCATAG/ideoxyU/TTCAGTTCAATTGCAGAAGGAG |
|  | Screening LA Forward  Screening RA Reverse | Ttggactccacctctatattgac  GCTGACCATGCTGCTATGAAC |
| **Primers for** **endogenously PIK3CA/p110α Y317F knock-in (pAAV backbone)** | | |
|  | Left arm Forward  Left arm Reverse | GGGAAAG/ideoxyU/ccatttcaaaattcagaccag  GGAGACA/ideoxyU/tcctaaccatctgaaaacatactac |
| PIK3CA locus  Y317F mutation KI | Right arm Forward  Right arm Reverse  Y317F mut Forward  Y317F mut Reverse | GGTCCCA/ideoxyU/gaacatccaaatctccgaatg  GGCATAG/ideoxyU/ccttacCTGGGATTGGAACAAG  CAGCTACACCATTTATGAATGGAG  CTCCATTCATAAATGGTGTAGCTG |
|  | Screening LA Forward  Screening RA Reverse | ttatcccctccaaccagtgg aatgtggggaatgattagca |
| **Primers for** **endogenously PIK3CA/p110α Y508F knock-in (pAAV backbone)** | | |
|  | Left arm Forward  Left arm Reverse | GGGAAAG/ideoxyU/tacatagGTGGAATGAATGGCTG  GGAGACA/ideoxyU/gcaatattggtcctagagttcatag |
| PIK3CA locus  Y508F mutation KI | Right arm Forward  Right arm Reverse  Y508F mut Forward  Y508F mut Reverse | GGTCCCA/ideoxyU/atagaattaagttctctatatgc  GGCATAG/ideoxyU/cccgagtagctgggattacag  AGGATTTAGCTTTTCCCACGCAG  CTGCGTGGGAAAAGCTAAATCCT |
|  | Screening LA Forward  Screening RA Reverse | tttgatgaagacttttcttgatgtatt aggtgtgatccacctaccccatctc |
| **sgRNA oligos surrounding PIK3CA/p110α Y317 or Y508 sites** | | |
| Y317F  sgRNA-1 | Forward  Reverse | caccGTAGTTCTACCCTTAACTGG  aaacCCAGTTAAGGGTAGAACTAC |
| Y317F  sgRNA-2 | Forward  Reverse | caccgACTGTAGTTCTACCCTTAAC  aaacGTTAAGGGTAGAACTACAGTc |
| Y317F  sgRNA-3 | Forward  Reverse | caccGACTACATATTAATCTGCAA  aaacTTGCAGATTAATATGTAGTC |
| Y317F  sgRNA-4 | Forward  Reverse | caccgTGTATAATAGCTAAGGTAGA  aaacTCTACCTTAGCTATTATACAc |
| Y508F  sgRNA-1 | Forward  Reverse | caccgTTATATATACATAAGAGAGA  aaacTCTCTCTTATGTATATATAAc |
| Y508F  sgRNA-2 | Forward  Reverse | caccgTAAGTATCAATTATAATCTG  aaacCAGATTATAATTGATACTTAc |
| Y508F  sgRNA-3 | Forward  Reverse | caccgCAATTATAATCTGTGGATTT  aaacAAATCCACAGATTATAATTGc |
| Y508F  sgRNA-4 | Forward  Reverse | caccGCCGACTATGTGTAAAGCAC  aaacGTGCTTTACACATAGTCGGC |
| **Subcloning primers** | | |
| SRC-pCMV | Forward (EcoRI)  Reverse (XhoI) | ccgGAATTCatgggtagcaacaagagcaag  ccgCTCGAGctagaggttctccccgggctg |
| Lyn-pCMV | Forward (EcoRI)  Reverse (XhoI) | ccgGAATTCatgggatgtataaaatcaaaag  ccgCTCGAGctaaggctgctgctggtattg |
| Fyn-pCMV | Forward (EcoRI)  Reverse (SalI) | ccgGAATTCatgggctgtgtgcaatgtaag  acgcGTCGACttacaggttttcaccaggttg |
| SRC-pET28a | Forward (EcoRI)  Reverse (XhoI) | ccgGAATTCatgggtagcaacaagagcaag  ccgCTCGAGctagaggttctccccgggctg |
| p110α-pCMV | Forward (BamHI)  Reverse (HindIII) | cgcGGATCCatgcctccacgaccatcatc  cccAAGCTTtcagttcaatgcatgctgtt |
| p110α Y317F-pCMV | Quickchange Forward  Quickchange Reverse | CAGCTACACCATTTATGAATGGAG  CTCCATTCATAAATGGTGTAGCTG |
| p110α Y508F-pCMV | Quickchange Forward  Quickchange Reverse | AGGATTTAGCTTTTCCCACGCAG  CTGCGTGGGAAAAGCTAAATCCT |
| p110α-pfastbac | Forward (BamHI)  Reverse (HindIII) | cgcGGATCCatgcctccacgaccatcatc  cccAAGCTTtcagttcaatgcatgctgtt |
| p85α-pfastbac | Forward (EcoRI)  Reverse (SalI) | ccgGAATTCatgagtgctgaggggtaccag  acgcGTCGACtcatcgcctctgctgtgcata |
| **qRT-PCR primers** | | |
| PIK3CA | Forward  Reverse | GAGGATGCCCAATTTGATGTTG  gaacagcaaaacctcgaacc |
| β-actin | Forward  Reverse | CATGTACGTTGCTATCCAGGC  CTCCTTAATGTCACGCACGAT |

**Table S4. List of antibodies**

| **Antibodies and Reagents** | **Source** | **Identifier** |
| --- | --- | --- |
| **Antibodies** | | |
| Mouse monoclonal antibody anti-FLAG | Sigma-Aldrich | Cat# F1804, RRID:AB_262044 |
| Mouse monoclonal antibody anti-Myc | Santa Cruz | Cat# sc-40, RRID:AB_627268 |
| Rabbit monoclonal antibody anti-p110α | Cell Signaling Technology | Cat# 4249, RRID:AB_2165248 |
| GAPDH | Cell Signaling Technology | Cat# 5174, RRID:AB_10622025 |
| p-Src Y416 | Cell Signaling Technology | Cat# 2101, RRID:AB_331697 |
| Src | Cell Signaling Technology | Cat# 2123, RRID:AB_2106047 |
| p-FAK Y397 | Cell Signaling Technology | Cat# 8556, RRID:AB_10891442 |
| FAK | Cell Signaling Technology | Cat# 3285, RRID:AB_2269034 |
| p-MLC2 T18/S19 | Cell Signaling Technology | Cat# 3674, RRID:AB_2147464 |
| MLC2 | Cell Signaling Technology | Cat# 8505, RRID:AB_2728760 |
| Rabbit monoclonal antibody anti-AKT pT308 | Cell Signaling Technology | Cat# 13038, RRID:AB_2629447 |
| Rabbit monoclonal antibody anti-AKT pS473 | Cell Signaling Technology | Cat# 4060, RRID:AB_2315049 |
| Rabbit monoclonal antibody anti-AKT | Cell Signaling Technology | Cat# 9272, RRID:AB_329827 |
| Rabbit polyclonal antibody anti-GSK-3β pS9 | Cell Signaling Technology | Cat# 9336, RRID:AB_331405 |
| Rabbit monoclonal antibody anti-GSK-3β | Cell Signaling Technology | Cat# 9315, RRID:AB_490890 |
| Rabbit monoclonal antibody anti-mTOR pS2448 | Cell Signaling Technology | Cat# 5536, RRID:AB_10691552 |
| Rabbit monoclonal antibody anti-mTOR | Cell Signaling Technology | Cat# 2983, RRID:AB_2105622 |
| Rabbit monoclonal antibody anti-p70 S6 kinase pS371 | Cell Signaling Technology | Cat# 9208, RRID:AB_330990 |
| Rabbit monoclonal antibody anti-anti-p70 S6 kinase | Cell Signaling Technology | Cat# 2708, RRID:AB_390722 |
| β-actin | Abcam | Cat# ab8226, RRID:AB_306371 |
| E-cadherin | Cell Signaling Technology | Cat# 3195, RRID:AB_2291471 |
| β-catenin | Cell Signaling Technology | Cat# 8480, RRID:AB_11127855 |
| EGFR | Proteintech | Cat# 18986-1-AP, RRID:AB_10596476 |
| Insulin Receptor β | Cell Signaling Technology | Cat# 3025, RRID:AB_2280448 |
| Csk | Cell Signaling Technology | Cat# 4980, RRID:AB_2276592 |
| Anti-Flag Affinity Gel | Bimake | Cat# B23101 |
| Anti-Myc tag Mouse mAb conjugated Agarose Beads | Engibody Biotechnology | Cat# AT0080 |
